# Cyclic peptides target the aromatic cage of a PHD-finger reader domain to modulate epigenetic protein function†

**DOI:** 10.1039/d2sc05944d

**Published:** 2023-04-17

**Authors:** Oliver D. Coleman, Jessica Macdonald, Ben Thomson, Jennifer A. Ward, Christopher J. Stubbs, Tom E. McAllister, Shane Clark, Siddique Amin, Yimang Cao, Martine I. Abboud, Yijia Zhang, Hitesh Sanganee, Kilian V. M. Huber, Tim D. W. Claridge, Akane Kawamura

**Affiliations:** a School of Natural and Environmental Sciences - Chemistry, Newcastle University Newcastle NE1 7RU UK akane.kawamura@ncl.ac.uk; b Department of Chemistry, University of Oxford, Chemistry Research Laboratory Mansfield Road Oxford OX1 3TA UK tim.claridge@chem.ox.ac.uk; c Radcliffe Department of Medicine, Wellcome Centre for Human Genetics, University of Oxford Roosevelt Drive Old Road Campus Oxford OX3 7BN UK; d Target Discovery Institute, Nuffield Department of Medicine, University of Oxford Old Road Campus Oxford OX3 7FZ UK; e Centre for Medicines Discovery, Nuffield Department of Medicine, University of Oxford Oxford OX3 7FZ UK; f Mechanistic and Structural Biology, Discovery Sciences, R&D, AstraZeneca Cambridge CB4 0WG UK; g Emerging Innovations Unit, Discovery Sciences, R&D, AstraZeneca Cambridge UK

## Abstract

Plant homeodomain fingers (PHD-fingers) are a family of reader domains that can recruit epigenetic proteins to specific histone modification sites. Many PHD-fingers recognise methylated lysines on histone tails and play crucial roles in transcriptional regulation, with their dysregulation linked to various human diseases. Despite their biological importance, chemical inhibitors for targeting PHD-fingers are very limited. Here we report a potent and selective *de novo* cyclic peptide inhibitor (OC9) targeting the *N*^ε^-trimethyllysine-binding PHD-fingers of the KDM7 histone demethylases, developed using mRNA display. OC9 disrupts PHD-finger interaction with histone H3K4me3 by engaging the *N*^ε^-methyllysine-binding aromatic cage through a valine, revealing a new non-lysine recognition motif for the PHD-fingers that does not require cation-π interaction. PHD-finger inhibition by OC9 impacted JmjC-domain mediated demethylase activity at H3K9me2, leading to inhibition of KDM7B (PHF8) but stimulation of KDM7A (KIAA1718), representing a new approach for selective allosteric modulation of demethylase activity. Chemoproteomic analysis showed selective engagement of OC9 with KDM7s in T cell lymphoblastic lymphoma SUP T1 cells. Our results highlight the utility of mRNA-display derived cyclic peptides for targeting challenging epigenetic reader proteins to probe their biology, and the broader potential of this approach for targeting protein–protein interactions.

## Abstract 2

We report the development of a potent and selective *de novo* cyclic peptide ligand for epigenetic methyllysine reader domain. The cyclic peptide binds at the aromatic cage of PHD-finger and modulates the JmjC lysine demethylase activity of KDM7.

## Graphical abstract

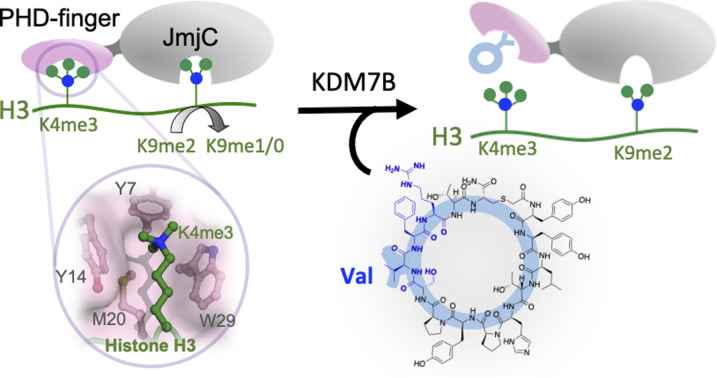

## Introduction

Post-translational modifications (PTMs) on histone proteins are an important part of overall epigenetic control.^1^ Histone marks such as lysine acetylation and methylation and the proteins that install, recognise, and remove these marks (‘writers’, ‘readers’ and ‘erasers’) contribute to transcriptional regulation.^2^ Maintenance of the dynamic histone PTM environment influences chromatin organisation,^3^ which can impact cellular function in health and disease. *N*^ε^-methylations of lysines on histones are PTMs recognised by a wide variety of chromatin reader domain families,^4^ including the plant homeodomain (PHD) fingers.^5^ PHD-fingers are a major family of epigenetic reader domains which are typically 50–80 amino acids in size and contain two zinc-coordinating Cys4–His–Cys3 motifs. At least 165 PHD-fingers have been found across 100 human proteins^6^ involved in a variety of biological processes.^7–11^ Many remain uncharacterised, but a substantial number of PHD-fingers act as ancillary domains that recognise histone lysine methylation states, commonly at histone H3 on lysine K4 (H3K4),^4,5^ to target their protein towards specific PTMs and exert influence over their primary function.^12^

PHD-fingers are prevalent among chromatin associated proteins, including >50 found in lysine methyltransferases (KMTs) and Jumonji-C (JmjC) histone demethylases (KDMs),^13,14^ which dynamically alter histone lysine methylation as part of epigenetic regulation in health and disease.^2,3,15–18^ The importance of PHD-fingers in such context is exemplified by KDM7B (PHF8, JHDM1F) which reads *N*^ε^-trimethyllysine at H3K4 (H3K4me3), generally considered an active histone mark at transcriptional start sites, to influence the linked JmjC-domain catalysed demethylation of H3K9me2.^19^ KDM7B is upregulated in melanoma to transcriptionally promote the TGFβ pathway through its demethylase activity and increase cell invasiveness.^20^ Inactivating mutations to either the catalytic JmjC-domain or PHD-finger greatly reduces metastasis in cell and animal studies, demonstrating that the PHD-finger function is crucial to its oncogenic activity.^20^ KDM7B is also involved in other cancer progressions, as well as neural and developmental processes,^21–26^ with other sub-family members KDM7A (KIAA1718) and KDM7C (PHF2) linked to similar conditions.^27–30^

Despite the biological importance, there are currently no potent and selective inhibitors for the PHD-fingers,^14,31,32^ which is in stark contrast to other families of chromatin reader domains (*e.g.* bromodomains, MBT, PWWP).^33–35^ The low druggability of the PHD-fingers with small molecules has been attributed to their shallow and open lysine binding pocket,^36^ and their extended histone binding interactions across the protein surface. H3K4me3 binding PHD-fingers are believed to interact with positively charged *N*^ε^-trimethyllysine primarily through cation–π interaction with their aromatic cage, typically consisted of two to four aromatic and hydrophobic amino acids, as well as *via* additional salt-bridging interactions.^37^ These features can allow some PHD-fingers to bind histones with remarkable methylation-state selectivity (*K*_D_ can differ 10–1000 fold).

Some histone peptide-derived PHD-finger inhibitors containing K4me3 as a targeting motif have been reported which show promise,^38,39^ however, achieving selectivity over other epigenetic proteins remains a major challenge.

Here we report the development of a *de novo* natural product-like cyclic peptide (CP) ligand OC9 targeting the PHD-finger of histone demethylase KDM7 sub-family. We used cyclic peptide mRNA-display to screen a library with >10^12^ diversity of structurally constrained 3D scaffolds to identify OC9, which exhibits nanomolar affinity and high selectivity against isolated proteins and in cellular lysate context. OC9 disrupts the PHD-finger–H3K4me3 interaction through binding at the aromatic cage *via* an unprecedented valine motif. Moreover, PHD-finger inhibition gives allosteric control of JmjC demethylase activity at H3K9me2, inhibiting KDM7B but stimulating KDM7A, representing specificity of functional modulation within the KDM sub-family members.

Our work provides new molecular insight into PHD-finger recognition and their role in fine-tuned epigenetic control, and a new PHD-finger targeting scaffold for further inhibitor development.

## Results and discussion

### PHD-finger targeted cyclic peptide discovery and profiling against KDM7s

**Fig. 1:**
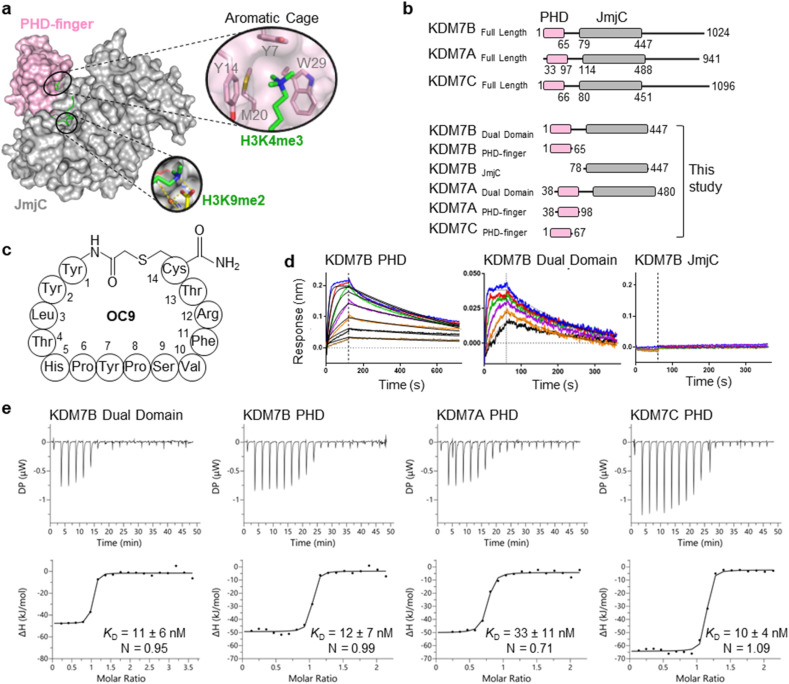
mRNA-display identifies high affinity cyclic peptides for KDM7B. (a) Crystal structure of KDM7B PHD finger (pink) and JmjC (grey) domains in complex with H3_1–24_K4me3K9me2 (green) and 2-oxoglutarate co-factor mimic *N*-oxalyl glycine (NOG, yellow) (PDB ID 3KV4). The ‘YYMW’ aromatic cage of the PHD-finger binds K4me3 and the JmjC demethylates K9me2. (b) KDM7 sub-family domain architectures, and the main KDM7 constructs used in this study. (c) Structure of OC9, discovered by mRNA-display. (d) BLI traces for association and dissociation (separated by vertical dotted line) of OC9 to KDM7B domains. Each peptide concentration (2-fold serial dilution) response is a different colour, with curve fittings (1 : 1 binding model) overlaid in black. See Fig. S5† for further info. (e) ITC binding curves of OC9 with KDM7 domains, fitted with a 1 : 1 binding model. See Fig. S10† for further info.

The challenge in developing inhibitors for PHD-fingers has, in part, been attributed to the shallow pocket and surface grooves for histone binding. In KDM7s, the PHD-finger is found in tandem to the JmjC demethylase domain to sandwich the histone H3 tail for positioning and catalysis, which creates a more enclosed ‘druggable pocket’^40^ (Fig. 1a). We therefore sought to generate *de novo* cyclic peptides (CPs) targeting the PHD-finger of the dual domain (PHD-JmjC) of KDM7B using an affinity-based RaPID mRNA-display screening.^41–43^ In brief, a 12–14mer CP-mRNA library with >10^12^ diversity was constructed using a DNA library, encoding for peptides with 10–12mer variable region (NNK) flanked by initiator methionine and *C*-terminal cysteine, followed by a (Gly-Ser)_3_ linker and an amber stop codon. Following *in vitro* transcription, puromycin ligation and cell-free *in vitro* translation, a CP libray conjugated to its encoding mRNA (CP-mRNA library) was generated. Flexizyme coupled with codon reprogramming allowed the replacement of initiator methionine with chloro-acetyl-l-tyrosine to spontaneously cyclise the translated peptide by thioether linkage with a fixed *C*-terminal cysteine.^44^ Reverse transcription further generated a stabilised heteroduplex mRNA/cDNA conjugated CP-library, which was applied to bead-immobilised KDM7B. Binding CP sequences were enriched iteratively over five rounds (Fig. S1†). Candidate binding sequences were identified by next-generation sequencing (NGS), and some of the most enriched sequences were synthesised using solid-phase peptide synthesis (SPPS) without the linker (designated OC#).

Six CPs (Fig. S1†) were initially characterised by bio-layer interferometry (BLI) to examine binding to KDM7B. Different CPs exhibited distinctive binding profiles to KDM7B protein constructs (dual domain, PHD-finger, JmjC-domain, Fig. 1b and S2†). CP hits were clustered into those that are: (i) specific to the dual domain only (OC3), (ii) specific to a single domain (OC4 – JmjC, OC9 – PHD) or (iii) non-binders. OC9 (Fig. 1c) exhibited near-equivalent nanomolar affinities and dissociative half-lives in the order of minutes for both the dual domain (*K*_D_^BLI^ = 6 nM, *t*_1/2_ = 2.8 min) and PHD-finger (*K*_D_^BLI^ = 4 nM, *t*_1/2_ = 8.1 min) of KDM7B, with no affinity for the isolated JmjC, indicating that it likely binds the PHD-finger alone (Fig. 1d). Thus, OC9 was prioritised for further development. Isothermal titration calorimetry (ITC) confirmed OC9 affinity for KDM7B with *K*_D_ values of 11 nM (dual domain) and 12 nM (PHD-finger) (Fig. 1e). OC9 also showed similar affinity and binding profiles against the related KDM7A and KDM7C dual domains and PHD-fingers, consistent with high KDM7 PHD-finger sequence homology (>80% identity to KDM7B, Fig. S3†). The affinities of OC9 against the KDM7 PHD-fingers were at least 10-fold greater than the natural histone peptide ligand (H3_1–21_K4me3, ITC *K*_D_ = 115 nM to KDM7B PHD-finger). For further BLI and ITC data, see Fig. S4–S10.†

**Fig. 2:**
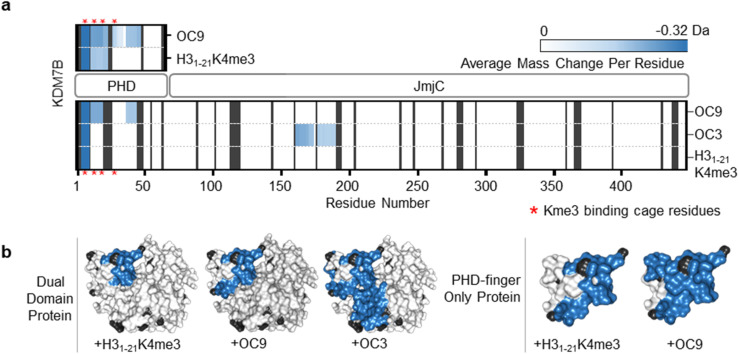
OC9 specifically targets the PHD-finger. (a) Regions of KDM7B dual domain and PHD-finger proteins affected in HDX-MS after 5 minutes of incubation with OC9, OC3 or H3_1–21_K4me3, assessed in triplicate relative to DMSO. The residue level view was generated by averaging mass changes across overlapping peptide fragments. Supporting data available in ESI.† K4me3 binding aromatic cage residues (‘YYMW’ Fig. 1a) are indicated by red asterix. (b) Combined HDX-MS data for all time points (1, 5, 15, 30, 60 minutes) for dual domain and PHD-finger protein mapped to a structure (PDB ID 3KV4) as protected (blue), not significantly affected (white) and without sufficient coverage (black).

To further evaluate the interactions of OC9 with KDM7B we used hydrogen–deuterium exchange mass spectrometry (HDX-MS), which informs on protein dynamics by following deuterium incorporation into the protein amide backbone (Fig. 2). Briefly, protein was incubated with or without ligand (CP or H3_1–21_K4me3) in deuterated buffer over time and quenched by acidification and cooling. Proteolysis and LC-MS analysis of the nascent peptides and comparison with a DMSO-control identified protein regions affected by ligand binding. For the dual domain, OC9 and H3_1–21_K4me3 only reduced deuterium uptake in peptide fragments within the PHD-finger (Fig. 2, S11–S14, HDX-MS ESI†). The lack of observable effect on the JmjC by H3_1–21_K4me3 could be due to a very weak or non-substantial interaction of its unmethylated H3K9 with the JmjC. However, the dual domain specific CP OC3 (ITC *K*_D_ = 163 nM, Fig. S9†) affected deuterium uptake in both domains, confirming that dual domain binding can be mapped using HDX-MS.

Further HDX-MS on KDM7B PHD-finger alone showed that H3_1–21_K4me3 reduced deuterium uptake across a substantial proportion of the domain over a 60 minute time-course, whilst OC9 affected an even greater proportion including the regions that contain the function-critical aromatic cage residues ‘YYMW’ (Fig. 2a and S11A†). Thus, OC9 exclusively binds at the PHD-finger in agreement with BLI and ITC.

### Cyclic peptide OC9 potently inhibits KDM7 PHD-finger binding to H3K4me3

**Fig. 3:**
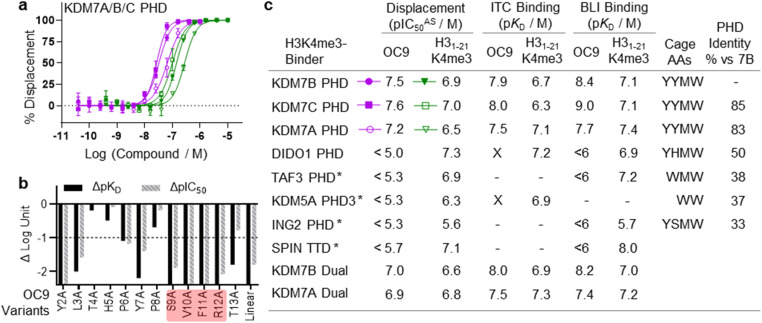
OC9 selectively inhibits protein–peptide interactions of KDM7 PHD-fingers and histone H3K4me3. (a) Competitive displacement of H3K4me3 peptide from KDM7 PHD-fingers is observed for OC9 – a representative plot is shown (key in table, c). (b) SAR analysis of OC9 sequence by alanine scanning and linearisation, using BLI and AlphaScreen assays. An important SVFR motif is highlighted (red). (c) Comparison of OC9 and H3_1–21_K4me3 displacement efficacies (pIC_50_ values from two independent experiments each in technical triplicate, or * once at least in duplicate) and binding affinities against several H3K4me3 reader proteins (X = no binding detected, — = not tested). Residues comprising the aromatic cage are noted, and PHD-finger identities are from canonical sequence alignment by BLASTP.

The functional effect of OC9 on the PHD-finger was further assessed using an AlphaScreen (AS) based proximity assay. OC9 inhibited the protein-peptide interactions of His-tagged KDM7B PHD-finger and biotinylated H3_1–21_K4me3 ^14^ at a displacement IC_50_^AS^ of 32 nM, at a higher potency than unlabelled H3_1–21_K4me3 (135 nM), demonstrating OC9 to be a potent competitive inhibitor of histone H3K4me3 peptide. Similar trends were observed for OC9 against other KDM7 sub-family PHD-finger and dual domain proteins (Fig. 3a, c and S15†).

### Evaluating OC9 structure–activity relationships identified key motifs for PHD-finger engagement

To gain further molecular understanding of the OC9–KDM7B PHD-finger interaction, the structure activity relationship (SAR) of OC9 was investigated using affinity (BLI) and displacement (AS) assays (Fig. 3b and S16–S19†). In general, there was good agreement between the affinity and functional activity of OC9 derivatives. Linear OC9 showed substantially reduced potency (>100-fold), indicating conformational constraint induced by cyclisation was important. Alanine scanning and other peptide substitutions revealed that V10A and F11A variants have significantly reduced potency (>100-fold) and the adjacent S9A and R12A variants were also much weaker, highlighting ‘SVFR’ as a key binding motif in OC9.

Other positions (L3, Y7) were also important as they showed 10 to 100-fold reductions in potency when replaced with alanine.^45–47^ Residues T4 and H5 tolerated l-alanine and d-alanine substitutions, whilst P8 only tolerated l-alanine. All tyrosines could be exchanged for phenylalanine, individually and in combination, with minimal impact. Bioinformatic analysis of NGS data from mRNA-display supported these empirical SAR findings, with a Logo plot for the CP family cluster related to OC9 (902 distinct sequences in total) highlighting the enrichment of sequences with the ‘SVXR’ motif (where X = F/Y/W), aromatic residues favoured at positions 2, 7, 11 and greater variability found at positions 4 and 5 (Fig. S20†). Notably, V10 was the most highly conserved residue. Characterisation of an OC9 variant with the second-most abundant residue (leucine) at position 10 (OC9 V10L) resulted in >80-fold reduction in *K*_D_ and >75-fold reduction in *t*_1/2_ in BLI, and 10-fold reduction in IC_50_^AS^ compared to OC9 (Fig. S16, S18B and S19†), in line with its significantly lower enrichment relative to OC9 by NGS (0.003% *vs.* 3.2% abundance). Thus, future SAR work may be accelerated by further exploiting such NGS-based peptide family analysis.

### Protein NMR revealed OC9 valine contacts the aromatic cage of the PHD-finger and is crucial for binding

**Fig. 4:**
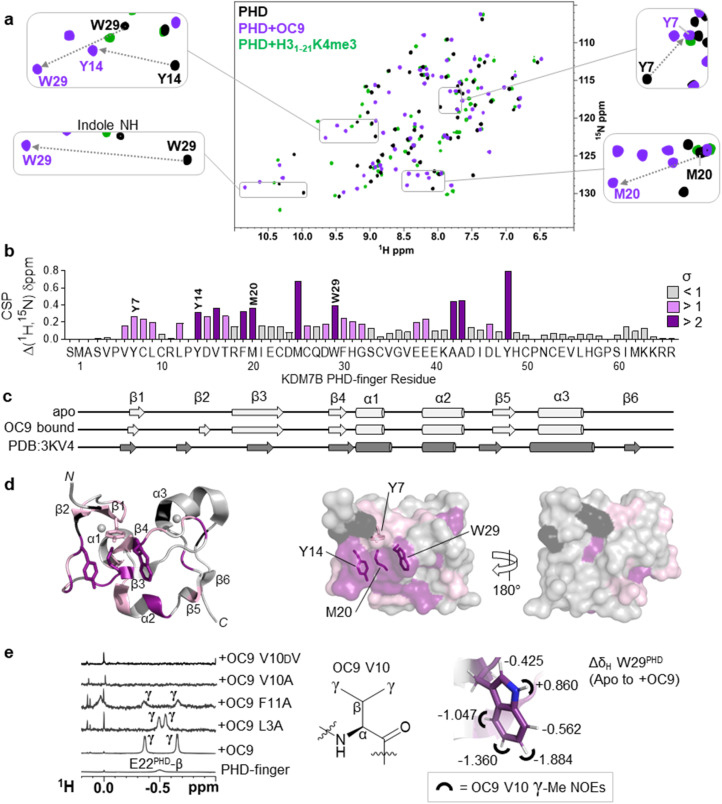
Protein NMR reveals valine contacting of the aromatic cage. (a) ^1^H–^15^N HSQC of KDM7B PHD-finger in the apo (black) or bound forms with OC9 (purple, 1 : 1) or H3_1–21_K4me3 (green, 3 : 1). (b) ^1^H and ^15^N chemical shift changes for apo *vs.* + OC9 were combined and weighted to give chemical shift perturbation (CSP) (Δ*δ*) values. Standard deviation (*σ*) of Δ*δ* are indicated (>1 pink, >2 purple). (c) TALOS+ predicts similar secondary structure elements in apo and OC9-bound solution protein complexes (light grey) to those found in PDB ID 3KV4 (dark grey). (d) Mapping of CSP *σ* from (b) onto PDB ID 3KV4 shows OC9 binding surfaces primarily along one face, which presents the cage (annotated residues). (e) Shielded ^1^H-NMR signals centred around −0.5 ppm belonging to the OC9 V10 γ-methyl groups are observed in some OC9–PHD complexes, but not in apo protein, or complexes with OC9 V10A and OC9 V10dV. The V10^OC9^ γ-methyl signals have NOE interactions (black semi-circles) with the W29^PHD^ indole, which itself is shielded upon binding of OC9 (indicated by Δ*δ*_H_ values).

To better elucidate OC9 interactions at a single residue level, we conducted protein NMR studies using isotopically labelled PHD-finger.^48^ Comparison of ^1^H–^15^N HSQC spectra for isotopically labelled apo and OC9-bound protein (Fig. 4a), independently assigned using ^13^C,^15^N 3D experiments (Fig. S21, NMR ESI†), identified 24 out of 66 protein residues with significant (standard deviation, *s* > 1) chemical shift perturbation (CSP, Δ*δ*) of their backbone amides (Fig. 4b).

OC9 affected the PHD-finger differently to H3_1–21_K4me3 based on spectra overlay, and was in slow exchange consistent with a slow off-rate on the NMR timescale and high affinity. Among the residues affected by OC9 binding, all four aromatic cage residues (Y7^PHD^, Y14^PHD^, M20^PHD^, W29^PHD^) were shifted, with changes in the W29^PHD^ indole NH also observed (Δ*δ*_H_ = +0.86 ppm). The Y14^PHD^, M20^PHD^ and W29^PHD^ cage residues also exhibited changes in their ^1^H amide temperature coefficients upon OC9 complexation (Fig. S22†) to further suggest the aromatic cage is directly affected, despite absence of any lysine residue in OC9. Other affected residues included F19^PHD^ and Y48^PHD^ which, alongside I21^PHD^, E22^PHD^ and I45^PHD^, have been found to interact with H3 residues A1, R2 and T3 in crystallographic studies^19^ and so their disruption could also contribute to observed OC9 histone displacement.

The PHD-finger remained well folded despite the extensive number of affected residues, as overall ^1^H–^15^N HSQC signals were well dispersed with similar secondary structure elements in solution predicted by TALOS+ ^49^ for apo and bound protein using chemical shift data (Fig. 4c and S23†). These elements were in broad agreement with previous X-ray crystallography (PDB ID 3KV4),^19^ likely due to the stabilising influence of two zinc coordination centres. Mapping of all affected residues showed they cumulatively formed a probable binding interface involving the β1, β2, β3, and β4 strands and α2 region, with negligible effect on α1 and α3 on the reverse face of the PHD-finger (Fig. 4d), in reasonable agreement with HDX-MS affected regions.

^1^H-NMR data also revealed a pair of distinct signals in the OC9 complex around −0.5 ppm (Fig. 4e, S24A and S25†). Titration of OC9 L3A and F11A saw these signals progressively increase, with their overall ^1^H–^15^N HSQC shift patterns indicating binding surfaces similar to OC9 (Fig. S26–S31†). However, the −0.5 ppm signals were not observed with OC9 V10A, lacking γ-methyl groups (Fig. 4e). Subsequent TOCSY and NOESY experiments did attribute the −0.5 ppm resonances to the two V10^OC9^ side chain γ-methyl groups, and also, *via* NOEs, found them to be in close proximity to the W29^PHD^ indole ring (Fig. 4e and S32†). A 2D-TOCSY investigation showed cage residue W29^PHD^ exhibited shielded indole proton ^1^H chemical shifts (5–6 ppm) in the OC9 complex relative to the apo PHD-finger (Fig. 4e and S33†), which could result from shielding induced by V10^OC9^ or adjacent residues.

Notably, inversion of the valine stereocentre in OC9 V10dV severely weakened affinity (NMR *K*_D_ = 503 ± 1 μM, p*K*_D_ < 3.3, supported by BLI *K*_D_ > 10 μM, Fig. S34†) and did not present the shielded methyl signals. Protein ^1^H-NMR also showed clear changes in the aromatic region (∼6 ppm) upon OC9 complexation with new signals appearing, but weak-binding OC9 V10dV instead elicited broadening of peaks (Fig. S24A†). Together, this suggests the presence and spatial orientation of the OC9 valine, imposed by conformational constraint of cyclisation, is critical to OC9 potency by binding at the aromatic cage to disrupt the methyllysine host site. This contrasts with the ability of H3K4me3 reader domains (PHD-fingers and tandem-Tudor) to accommodate d-K4me3 H3 derivatives (albeit with weaker affinity than l-K4me3).^50^

Interestingly, when V10 was replaced with Kme3 in OC9 V10Kme3, PHD-finger binding was moderately retained (*K*_D_^BLI^ = 533 nM, IC_50_^AS^ = 316 nM) whereas no binding was observed when replaced with K (OC9 V10K) (*K*_D_^BLI^, IC_50_^AS^ > 10 μM) (Fig. S16, S18B and S19†). The higher affinity for the methylated over unmethylated lysine variant of OC9 reflects the pattern of histone PTM recognition for KDM7 PHD-fingers (Fig. S8†) and provides further evidence for the engagement of OC9 position 10 with the aromatic cage. The binding of Kme3 in KDM7 and other PHD-fingers is proposed to be driven by a combination of favourable cation–π interaction between the positively charged quaternary ammonium group and the electron-rich aromatic residues, and the displacement of high-energy water molecules within the cage.^52^ However, binding to an aromatic cage without a cation–π interaction, as proposed for OC9 valine insertion, is precedented by the all-carbon, neutral, non-natural *tert*-butyl norleucine (*t*-BuNle) substituted H3K4 derived peptides which retain affinity against Kme3-binding PHD-fingers.^52,53^ In such cases, dispersion forces and hydrophobic effects are proposed to drive the aromatic cage engagement, as exemplified for CBX5 chromodomain, where affinity increases with increasing size of alkyl substituent on lysine ε-*N*.^54^

Further characterisation of OC9 binding was carried out from partial ^1^H chemical shift assignments derived for bound-OC9 obtained from isotope filtered 2D experiments (Fig. S24B†). While multiple interconverting conformers were observed for free OC9 (Fig. S24A†), only a single conformer population was observed when OC9 was bound to PHD-finger (Fig. S24B†). The conformer switching in the free form is likely driven by isomerism of the two prolines present in OC9. Substitution of OC9 with 4-fluoroproline at P6 gave higher affinity (>10-fold) for the *trans*-fluoro *exo*-puckered ring favouring the *trans*-amide bond^45–47^ over the *cis*-fluoro (Fig. S18 and S19†), suggesting *trans*-to be the more active PHD-finger binding conformer. In addition to the very substantially shielded ^1^H resonances of the OC9 V10 sidechain, the bound-OC9 NMR data also showed that the ortho aromatic protons of F11^OC9^ were similarly shielded by ∼1.3 ppm (relative to random coil values^51^), appearing at 6.0 ppm. Although no clear NOEs could be observed between the shielded F11^OC9^ ortho protons and W29^PHD^, NOEs could be identified to the spatially nearby I21^PHD^ methyl groups. The beta protons of R12^OC9^ also experienced appreciable shielding (∼1 ppm *versus* random coil). The H3R2 residue is known to anchor to a cage-adjacent polar/acidic patch of the KDM7 PHD-finger through a guanidinium-carboxylate salt bridge to E22^PHD^ to assist binding,^45–47^ with similar concomitant recognition of unmodified H3R2 and H3K4me3 also characteristic of many other PHD-fingers including BPTF, ING2 and KDM5A PHD3.^6^ Thus, since variant OC9 R12A also exhibited a considerably faster off-rate (∼100×) by BLI, R12^OC9^ could plausibly mimic the anchoring role of the histone H3R2 residue to control OC9 residence time. Collectively, these data suggest the OC9 V10-F11-R12 residues to be interacting significantly with the β2–β3–α2 face of the PHD finger, although there is insufficient data to definitively position OC9.

### Cyclic peptide OC9 is a highly KDM7 selective PHD-finger inhibitor

As a specific valine insertion at the aromatic cage is itself nonetheless unprecedented, we evaluated the selectivity of OC9 across a panel of H3K4me3 chromatin reader modules. The KDM7 PHD-fingers are most closely related to the DIDO1 PHD-finger,^55,56^ with its aromatic cage (YHMW) very similar to KDM7 (YYMW) (Fig. S35†). Despite nanomolar affinity of DIDO1 for H3K4me3 peptide, OC9 had no measurable affinity for DIDO1 by BLI, ITC or AS (Fig. 3a, S36 and S37†). Additionally, OC9 did not show binding or displacement of H3K4me3 from other H3K4me3-binders, including PHD-fingers KDM5A PHD3, ING2, TAF3 and the SPIN1 triple tudor domain (Fig. 3a and S37†). Of the 24 residues in KDM7B that experience significant CSP by NMR, only three and five residues differ in identity for KDM7C and KDM7A respectively, but 13 differ for DIDO1 which also has an additional four residue insertion (Fig. S38†). Despite sharing the same histone H3K4me3 ligand, the proposed extended OC9-PHD binding surface of KDM7 may explain the high degree of selectivity over other reader domains.

### PHD-finger inhibition by OC9 differentially regulates KDM7 JmjC demethylase activity

We next investigated whether inhibition of KDM7 PHD-fingers by OC9 would impact demethylase activity on histones (Fig. S39 and S40†). H3K9me2 demethylation *via* the JmjC domain is ordinarily enhanced (>10 fold) in the presence of K4me3 for KDM7B, but substantially reduced for KDM7A.^19^ The inter-domain spacing between the PHD-finger and JmjC domain is proposed to contribute to this, where the KDM7B linker length is optimal for H3K4me3K9me2 dual mark binding but sub-optimal for KDM7A.^57^ Thus, we hypothesised PHD-finger inhibition is likely to affect demethylase activity.

**Fig. 5:**
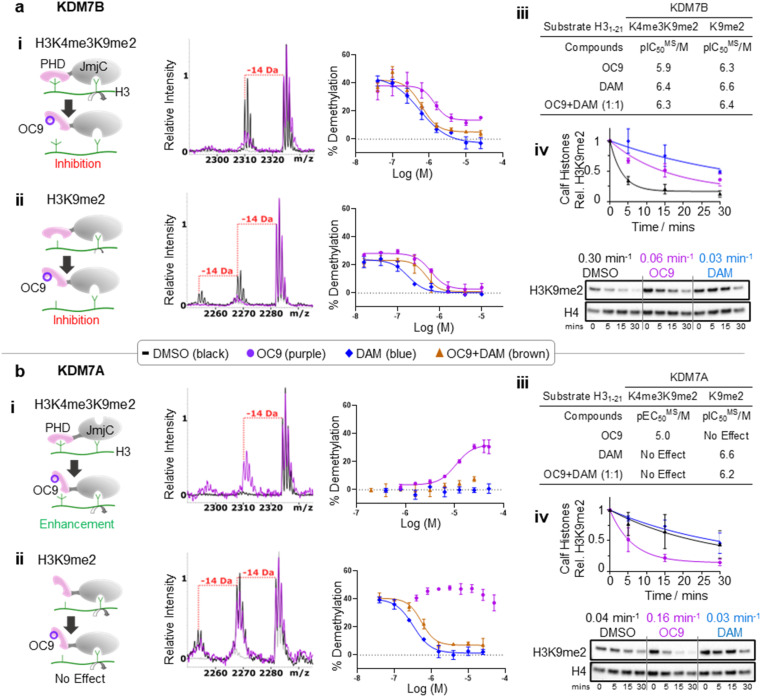
KDM7 PHD-finger inhibition by OC9 modulates dual domain JmjC activity. (a) (i–iii) OC9 inhibits demethylation of H3K9me2, with or without K4me3, by KDM7B (0.4 μM) on H3 peptide (2.5 μM). (iv) OC9 (20 μM) also inhibits KDM7B (1 μM) demethylation of calf histones (50 μg). (b) (i–iii) OC9 enhances demethylation of H3K4me3K9me2 by KDM7A (0.55 μM) on H3 peptide (2.5 μM), and (iv) enhances KDM7A (0.5 μM) demethylation of calf histone extracts (50 μg). Peptide demethylation assays analysed by MALDI-TOF-MS, with potency comparisons of *N* = 2 experiments in technical triplicate. Inset MALDI-TOF-MS overlays are from respective highest OC9 concentration points compared to DMSO alone. KDM7 PHD-finger only proteins had no demethylase activity (Fig. S40†). Calf histone H3K9me2 was quantitated relative to H4 band intensities at each time point, normalised to DMSO at *t* = 0. Full Western blots available in Fig. S42.† Relative rates (*K*_rel_ min^−1^) from a one-phase decay model, average from *N* = 3 independent experiments.

We tested the effect of OC9 on KDM7B demethylation of H3_1–21_K4me3K9me2 and the less-efficient H3_1–21_K9me2 substrate by MALDI-TOF MS based assay.^14,19^ OC9 inhibited KDM7B demethylation of both substrates with similar potencies, with IC_50_ values of 1.19 μM and 0.51 μM respectively (Fig. 5a, S39 and S40†). Given the lack of affinity of unmethylated H3K4 for the PHD-finger (Fig. S8†),^19^ the inhibitory effect of OC9 on H3_1–21_K9me2 demethylation was unexpected, but highlights the likely important allosteric role of the PHD-finger beyond PTM recognition. The potencies of OC9 were comparable to that of the KDM2/7 JmjC-active site small molecule inhibitor daminozide (DAM),^58^ and an equimolar mix of OC9 and DAM showed an additive inhibitory effect. Inhibition by OC9 was further confirmed by an orthogonal AS activity assay^58^ (Fig. S41†). OC9 also reduced the demethylation activity of KDM7B on H3K9me2 in calf histone extracts, which possess naturally occurring combinatorial PTMs (Fig. 5a and S42†). This suggests KDM7B demethylase activity against H3K9me2 is largely dependent on PHD-finger driven positioning of the *N*-terminal histone H3 tail regardless of H3K4 methylation state. As KDM7B aromatic cage mutation results in the same phenotypic outcome as JmjC catalytic site inactivation (*e.g.* KDM7B demethylase-dependent melanoma metastasis^20^), our results emphasise the crucial role of PHD-fingers in regulating KDM7 demethylase activity and the need to explore PHD-finger viability as therapeutic targets.

In contrast to KDM7B, KDM7A lacks activity against H3K4me3K9me2, but demethylates H3_1–21_K9me2.^57^ OC9, however, restored the activity of KDM7A against H3_1–21_K4me3K9me2 from <5% to a level (∼30%) that approached demethylation on H3_1–21_K9me2 with DMSO alone (∼40%) (Fig. 5b and S39†). Daminozide remained inhibitory against KDM7A and prevented restoration of activity in the equimolar mix with OC9. Activity enhancement by OC9 translated to an increased rate of demethylation against calf histone extracts (Fig. 5b and S42†). These combined results indicated OC9 blocks PHD-finger binding to K4me3 to allow demethylation of K9me2 by KDM7A, and further emphasises that OC9 binding is confined to the PHD-finger. The effect of PHD-finger inhibition by OC9 for KDM7C is unclear, due to its lack of demethylase activity *in vitro*.^59^ Due to the negligible permeability of OC9 in Caco-2 cell assay, the activity was not tested in cells. Further optimisations are likely required to improve the cellular permeability of OC9.

Screening against other isolated KDM sub-families (KDM2/3/4/5/6) with histone peptide substrates saw no significant inhibition by OC9. The panel included full-length recombinant KDM2A and KDM2B which have the closest JmjC-domains to KDM7s, but with unrelated PHD-fingers (Fig. S43 and S44†). Hence, OC9 not only represents the first selective and potent inhibitor for the PHD-fingers, but also of the demethylase activity for the KDM7 sub-family.^60^

While the use of *de novo* CPs to selectively inhibit epigenetic eraser and reader domains have been reported (*e.g.* KDM4 JmjC-domain,^61^ SIRT2,^62^ TET^63^ and bromodomain (BRD)^64^ sub-families), the PHD-finger targeting CPs provide unique context-dependent allosteric control *via* modulation of the tandem JmjC-domain. Thus, OC9 also exemplifies a PHD-finger targeting strategy for KDM inhibitor development, which can overcome selectivity challenges encountered by small molecules binding at the highly conserved active site of JmjC-domains.^58,65,66^

### PHD-finger dependent targeting of full-length KDM7s is highly selective in a cellular context

**Fig. 6:**
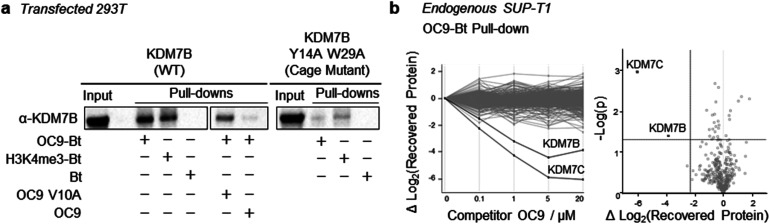
Selective capture of full-length KDM7 with OC9-Bt in nuclear lysates. (a) OC9-Bt, H3K4me3-Bt and Bt were used as affinity probes in HEK293T nuclear lysates, transiently transfected with wild type KDM7B, or PHD-finger mutant (Y14A, W29A). Capture was performed with 1 μM probe incubated in pre-cleared lysate, recovered with streptavidin-conjugated magnetic beads, then washed and eluted by heating in denaturing solution. 100-fold excess OC9 or OC9 V10A were used as competitors. (b) Proteomics analysis of endogenous pull-down samples (average of duplicates) from SUP-T1 nuclear lysate using 1 μM OC9-Bt affinity probe. (Left) Change in all protein (454) recoveries with competition by titration of unlabelled OC9. (Right) Volcano plot comparing relative abundance of pull-down content at 0 and 20 μM competition. The upper left quadrant indicates >5-fold difference in recovered protein (based on *T*-test difference of mean log 2 (LFQ) values) and *p*-value threshold <0.05.

To further probe the selectivity and mechanism of OC9 engagement in a cellular context, we conducted pull-down experiments with full length KDM7B using biotin (Bt) linked to OC9 (OC9-Bt, Fig. S45†) and H3K4me3-Bt. HEK293T cells were transiently transfected with either a full-length wild-type KDM7B, or an aromatic cage inactivating mutant KDM7B (Y14A, W29A).^20^ OC9-Bt and H3K4me3-Bt pull-down from nuclear lysates with streptavidin beads found wild-type KDM7B can be efficiently recovered from complex mixtures, while the recovery of the aromatic cage mutant was poor (Fig. 6a and S46†). Competition with a 100-fold excess of unmodified OC9 substantially reduced KDM7B recovery, but excess OC9 V10A variant did not reduce recovery so substantially. Together, these results confirmed that the binding of OC9-Bt to KDM7B is primarily driven by engagement with the intact aromatic cage and the V10^OC9^ residue is essential for this interaction.

A further chemoproteomic profiling analysis of endogenous protein capture by OC9-Bt from KDM7B-expressing T-cell lymphoblastic lymphoma SUP-T1 nuclear lysates^67^ identified 454 proteins. Only the relative abundance of KDM7B and KDM7C were significantly affected in a dose-dependent manner by competitive titration of unlabelled OC9 during pull-down (Fig. 6b, Proteomics ESI, and Western blot Fig. S47†). This proteomics data suggests remarkable selectivity and affinity of OC9 for KDM7 PHD-fingers, even in complex nuclear lysates abundant in chromatin associated proteins including H3K4me3 binders.

## Conclusions

Collectively, our findings demonstrate the power of display libraries in identifying efficient natural-product-like peptide scaffolds to bind potently and selectively against KDM7 PHD-fingers. Our work expands the motifs that can bind the KDM7 PHD-finger aromatic cages, which may have wider implications for KDM7 PHD-finger involvement beyond trimethyllysines and histones, whilst emphasising the possible diversity and versatility of PHD-fingers and potentially other reader domains which recognise methyllysine through similar aromatic cages (*e.g.* chromodomain, Tudor domain, MBTs). Indeed, some PHD-fingers have been reported to be involved in ternary complexes with histone and non-histone proteins,^8,68^ whilst some bind DNA,^69^ but others remain without assigned function. Our CPs can facilitate deeper exploration of PHD-finger contribution to epigenetic transcriptional control to probe the complex PTM interplay and protein–protein interactions of multi-domain epigenetic proteins. More broadly, this also paves a way towards chemical probe development for under-served PHD-fingers to better exploit their emerging therapeutic potential.^70^

## Data availability

The mass spectrometry proteomics data have been deposited to the ProteomeXchange Consortium *via* the PRIDE partner repository with the data set identifier PXD027151.

## Author contributions

Conceptualization by ODC and AK. Visualization by ODC, AK, CS and TDWC. Investigation and analysis of protein production by ODC with support from YZ. Investigation and analysis of mRNA display by ODC with support from TEM, and peptide synthesis and biophysical and biochemical characterisations by ODC with support from SC, TEM and SA. Investigation and analysis of protein NMR by ODC, JM, BT, CY, MIA, supervised by TDWC. Investigation and analysis of proteomics by ODC and JAW, supervised by KVMH. Investigation and analysis of HDXMS by CS and ODC. Analysis supported by HS. Writing by ODC, AK and TDWC with input from all authors. Supervision and overall project administration by AK.

## Conflicts of interest

There are no conflicts to declare.

## Supplementary Material

SC-014-D2SC05944D-s001

SC-014-D2SC05944D-s002

SC-014-D2SC05944D-s003

SC-014-D2SC05944D-s004
