# Supplementary material for: Cyclic peptides target the aromatic cage of a PHD-finger reader domain to modulate epigenetic protein function
Source: Chem Sci. 2023 Apr 17;14(26):7136–46. doi: 10.1039/d2sc05944d (PMC10321576; doi:10.1039/d2sc05944d)
Supplement: SC-014-D2SC05944D-s001 [file SC-014-D2SC05944D-s001.pdf]

Electronic Supplementary Material (ESI) for Chemical Science.
This journal is © The Royal Society of Chemistry 2023

Supplementary Information

Cyclic peptides target the aromatic cage of a PHD-finger reader domain to modulate epigenetic protein function

Oliver D. Coleman[1,2,3], Jessica Macdonald[2], Ben Thomson[2], Jennifer A. Ward[4,5], Christopher J. Stubbs[6], Tom E. McAllister[1,2,3], Shane Clark[2], Siddique Amin[1], Yimang Cao[2], Martine I. Abboud[2], Yijia Zhang[2], Hitesh Sanganee[7], Kilian V. M. Huber[4,5], Tim D. W. Claridge*[2], Akane Kawamura*[1,2,3]

[1] School of Natural and Environmental Sciences - Chemistry, Newcastle University, Newcastle NE1 7RU, United Kingdom

[2] Chemistry Research Laboratory, Department of Chemistry, University of Oxford, Mansfield Road, Oxford OX1 3TA, United Kingdom

[3] Radcliffe Department of Medicine, Wellcome Centre for Human Genetics, University of Oxford, Roosevelt Drive, Old Road Campus, Oxford, OX3 7BN, United Kingdom

[4] Target Discovery Institute, Nuffield Department of Medicine, University of Oxford, Old Road Campus, Oxford OX3 7FZ, United Kingdom

[5] Centre for Medicines Discovery, Nuffield Department of Medicine, University of Oxford, Oxford, OX3 7FZ, United Kingdom

[6] Mechanistic and Structural Biology, Discovery Sciences, R&D, AstraZeneca, Cambridge, CB4 0WG, United Kingdom

[7] Emerging Innovations Unit, Discovery Sciences, R&D, AstraZeneca, Cambridge, UK

*Corresponding author: akane.kawamura@ncl.ac.uk, tim.claridge@chem.ox.ac.uk

Supplementary Information Contents

Supplementary Figures **5**
Figure S1. Selection library enrichment and key cyclic peptide structures. 5
Figure S2. BLI for KDM7 proteins with cyclic peptides. 6
Figure S3. Sequence alignment of the KDM7A/B/C PHD-finger protein constructs. 7
Figure S4. BLI for KDM7 proteins with H3$_{1-21}$K4me3. 7
Figure S5. BLI data table for OC9, H3$_{1-21}$K4me3, OC3, OC4. 8
Figure S6. ITC titration curves for OC9 against KDM7 protein domains. 9
Figure S7. ITC titration curves for H3$_{1-21}$K4me3 against KDM7 protein domains. 10
Figure S8. ITC titration curves for His-Trx-KDM7B PHD. 11
Figure S9. ITC titration curve for OC3. 11
Figure S10. ITC results: Tabulated values from 1:1 binding model and conditions 12
Figure S11A. HDX-MS peptide level view for KDM7B dual domain. 13
Figure S11B. Continuation of Figure S11A. 14
Figure S12. HDX-MS residue level view for KDM7B dual domain. 15
Figure S13. HDX-MS peptide level view for KDM7B PHD finger. 16
Figure S14. HDX-MS residue level view for KDM7B PHD finger. 16
Figure S15. AlphaScreen displacement for OC9 with Kme3-reader domains. 17
Figure S16. AlphaScreen displacement for OC9 variants with KDM7B. 18
Figure S17. BLI for KDM7B PHD-finger protein with OC9 alanine scan variants. 19
Figure S18A. BLI for KDM7B PHD-finger protein with OC9 substitution variants. 20
Figure S18B. BLI for KDM7B PHD-finger protein with OC9 substitution variants. 21
Figure S19. BLI data table for OC9 ala scan and variant peptides. 22
Figure S20. Bioinformatic analysis of NGS results comparison to OC9 SAR. 23
Figure S21. ^{1}H-^{15}N HSQC spectra of the KDM7B PHD-finger, apo and in complex with OC9. 24
Figure S22. ^{1}H-^{15}N HSQC spectra temperature dependence of the KDM7B PHD-finger, apo and in complex with OC9. 25
Figure S23. TALOS+ prediction of PHD-finger secondary structure elements 26
Figure S24A. 1D ^{1}H-NMR spectra of KDM7B PHD-finger, apo and complex with OC9 or OC9 V10DV, and free OC9 27
Figure S24B. 1D ^{1}H-NMR chemical shifts for OC9 when complexed to KDM7B PHD-Finger 28
Figure S25. 1D ^{1}H-NMR spectra of OC9 variant titrations against KDM7B PHD-finger 29
Figure S26. ^{1}H-^{15}N HSQC spectrum of the KDM7B PHD-finger in complex with OC9 L3A. 30
Figure S27. ^{1}H-^{15}N HSQC spectrum of the KDM7B PHD-finger in complex with OC9 V10A. 31
Figure S28. ^{1}H-^{15}N HSQC spectrum of the KDM7B PHD-finger in complex with OC9 F11A. 32
Figure S29. ^{1}H-^{15}N HSQC spectrum of the KDM7B PHD-finger in complex with OC9 V10DV. 33
Figure S30. ^{1}H-^{15}N HSQC spectra overlay of the KDM7B PHD-finger complexed to different OC9 variants. 34

Figure S31. Surface mapping of NMR CSP. 35
Figure S32. NMR identification of OC9 V10 methyl signals. 36
Figure S33. 2D TOCSY protein NMR traces for cage residue W29. 37
Figure S34. OC9 V10DV affinity assessment. 38
Figure S35. PHD-finger phylogenetic tree and selected aromatic cage comparison. 39
Figure S36. ITC curves for DIDO1 and KDM5A vs OC9 and H3K4me3. 40
Figure S37. BLI traces of $H3_{1\text{-}21}$K4me3 and OC9 peptides with H3K4me3-reader domains. 41
Figure S38. Aligned KDM7A/B/C and DIDO1 PHD-finger sequences. 42
Figure S39. KDM7B and KDM7A dual domain demethylation assays by MALDI-TOF-MS. 42
Figure S40. KDM7B and KDM7A demethylation activity time-course assays by MALDI-TOF-MS. 43
Figure S41. KDM7B dual domain demethylase AlphaScreen assay. 43
Figure S42. Calf histone demethylation assay Western Blots. 44
Figure S43. KDM Cross Screening by MALDI-TOF-MS 45
Figure S44. KDM PHD-finger sequence alignments. 46
Figure S45. OC9-Biotin synthesis scheme. 47
Figure S46. Pull-downs from HEK293-T nuclear lysates. 48
Figure S47. Western blots for chemoproteomics pull-downs. 49
Supplementary Methods 51
mRNA Display Selection and Next-Generation Sequencing Analysis 51
General Procedure for His-tagged Protein Production 52
Isotopically (^{15}N, ^{13}C) Labelled Protein Production: 52
Biotinylated KDM7B Dual Domain Production 53
Expressed Protein Sequences 54
Protein Mass Spectrometry 55
Protein NMR 56
Peptide Synthesis 58
General Procedures 58
General Peptide Purification and Characterisation 59
Detailed Synthesis Procedure for OC9 on Chorus 60
Azide-Alkyne ‘Click’ Reaction for OC9-Biotin Synthesis 62
Peptide High-Resolution Mass Spectrometry Method 62
Peptide High-Resolution Mass Spectrometry Values 63
Peptide MALDI-TOF-MS Spectra 64
Peptide Analytical HPLC Traces 67
BioLayer Interferometry (BLI) 69
Isothermal Titration Calorimetry (ITC) 69
Hydrogen Deuterium Exchange Mass Spectrometry (HDX-MS) 70
AlphaScreen Displacement Assay 71
AlphaScreen Demethylation Assay 72
MALDI-TOF-MS KDM7 Activity Assay 73

KDM Selectivity Screening 73
Calf Histone Demethylation Assay 74
Cell permeability measurement for OC9 75
Western Blot 76
Wet Transfer: 76
Imaging: 76
Antibodies 76
Pull-down and Proteomics from SUP-T1 cells 77
Pull-down from Transfected HEK293T Cells 79
Full-Length KDM7B Plasmids and Mutagenesis 80
Supplementary References 81

Supplementary Figures

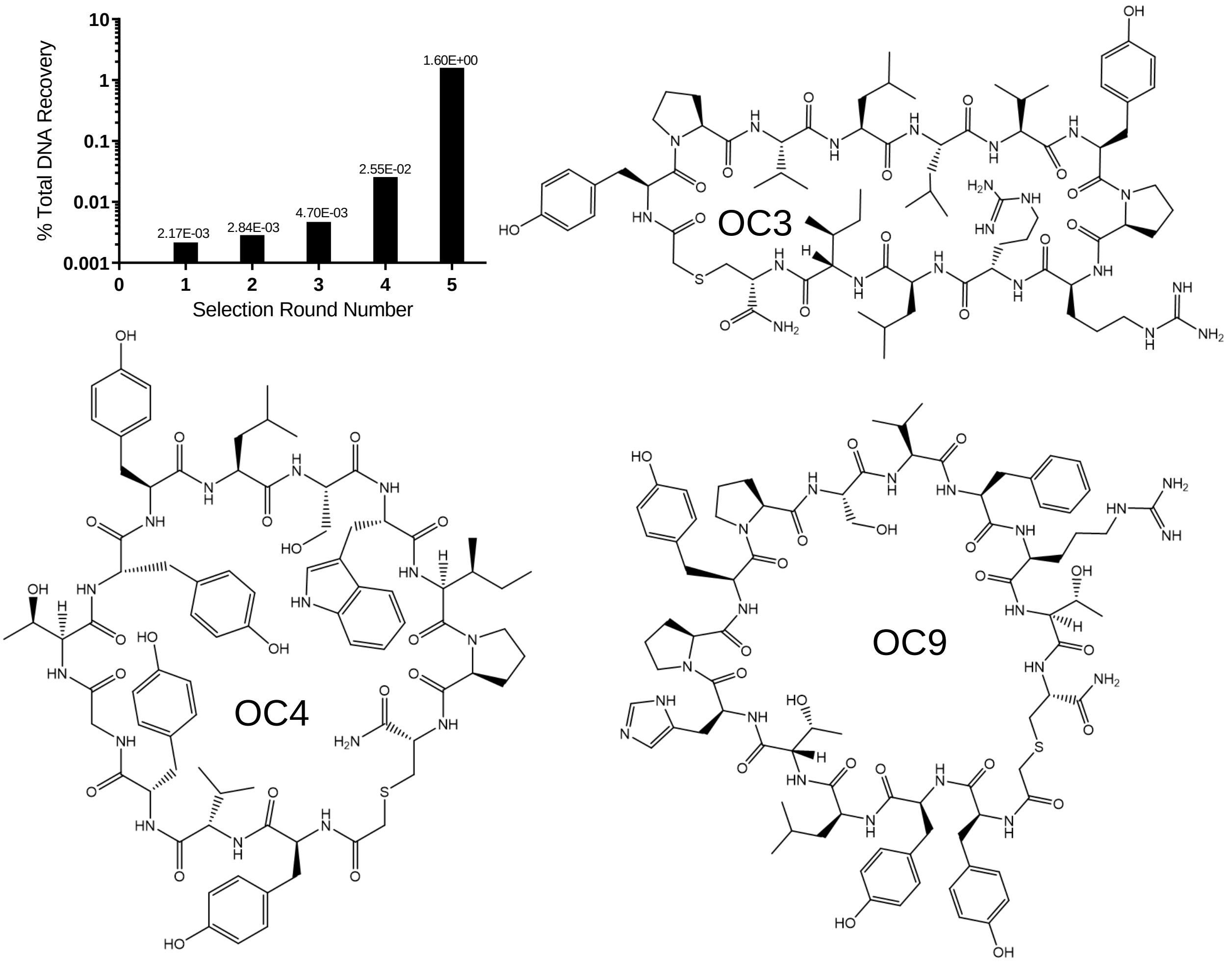

			BLI Screening		
**Peptide ID**	**Sequence**	**% A**	**KDM7B Dual**	**KDM7B PHD**	**KDM7B JmjC**
OC3	YPVLLVYPRRLIC	15.5	+	-	-
OC4	YVYGTYYLSWIPC	10.3	+	-	+
OC6	YLVRIYPNKLIWWC	4.5	NS	NS	NS
OC8	YPYILYPSVYTTNC	3.8	-		
OC9	YYLTHPYPSVFRTC	3.0	+	+	-
OC10	YIVPSPIWYHWVAC	2.9	-		
$H3_{1-21}K4me3$	ART[Kme3]QTARKSTGGKAPRKQLA		+	+	-

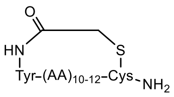

Figure S1. Selection library enrichment and key cyclic peptide structures.

Enrichment of the cyclic peptide NNK10-12 library against KDM7B dual domain. The recovery of DNA was determined by qPCR, using DNA standards generated by reverse transcription of an unbiased mRNA NNK starting library. Each recovery was calculated by taking the total quantity of DNA present at the end of the selection round, following magnetic bead extraction of binding sequences, as a percentage of the total quantity of DNA present in the pool immediately prior to selection. The structures of OC3, OC4 and OC9 are shown, and some of the most enriched sequences are tabulated. All OC peptides are cyclic, with thioether bond between *N*- and *C*- terminal amino acids. '%A' in the table represents percentage abundance of a given peptide sequence in the overall library count after round 5 NGS, with qualitative assessment of binding to KDM7B domains by BLI stated. (+) = binding, (-) = no binding, (NS) = non-specific.

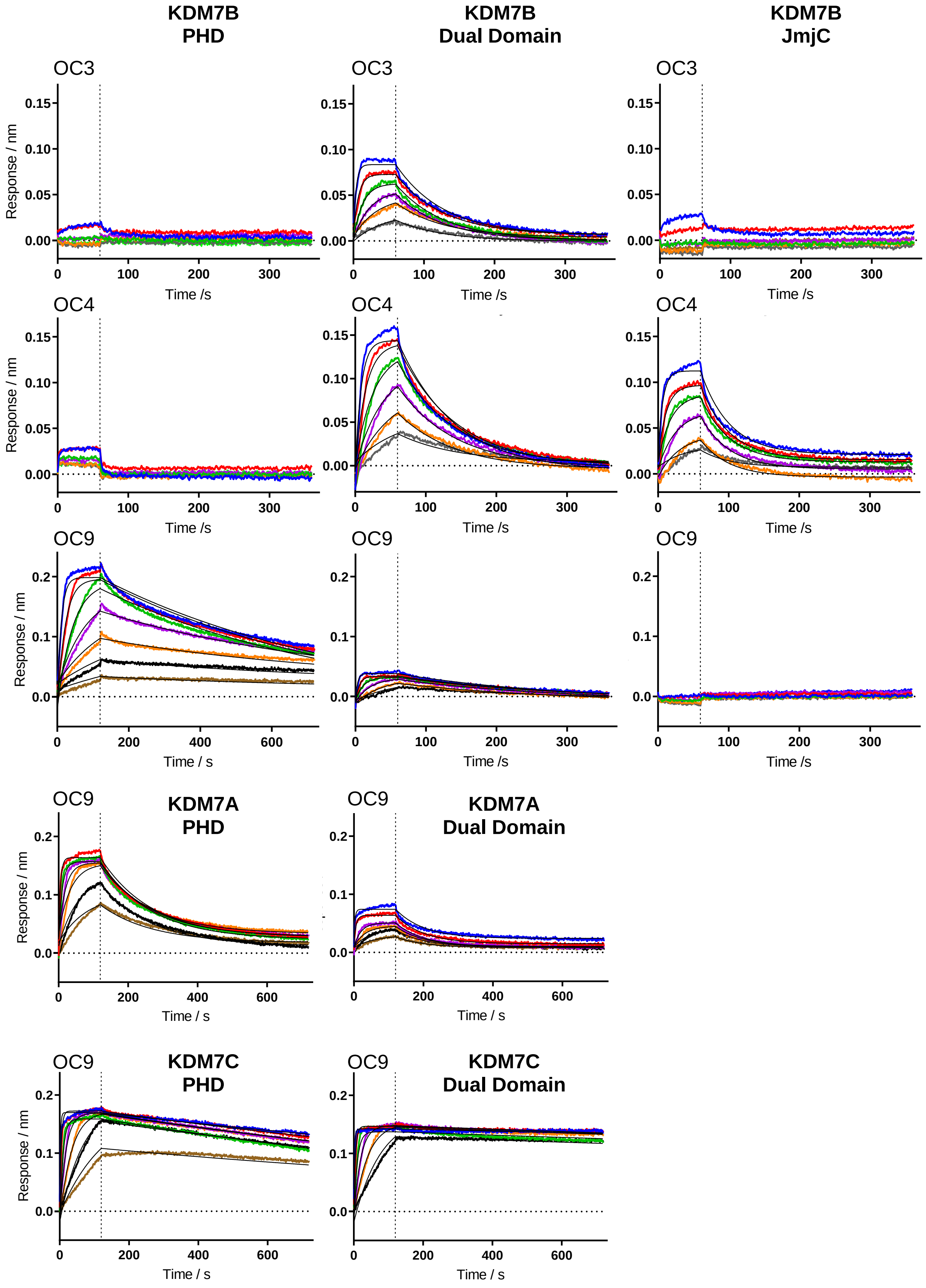

Figure S2. BLI for KDM7 proteins with cyclic peptides.

BLI traces for binding to KDM7 protein domains immobilised by *C*-terminal His-tag on Ni-NTA biosensors, against cyclic peptides. Response trace for each peptide concentration is represented by a different colour, with the fitted curves overlaid in black. See Figure S5 for results of fit and the top concentration plotted, with 2-fold dilutions thereafter. Dual domain = PHD+JmjC.

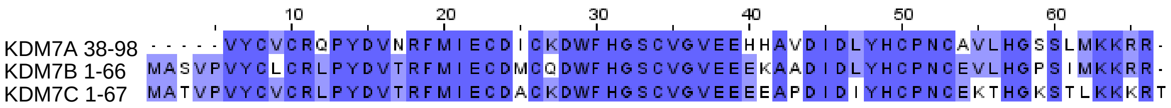

Figure S3. Sequence alignment of the KDM7A/B/C PHD-finger protein constructs.

Residues conserved within all three PHD-fingers are highlighted in dark blue, those conserved within at least two of the three are in light blue, and present in only one of the three in white. Alignment and graphic generated from JalView. Constructs those used in binding assays.

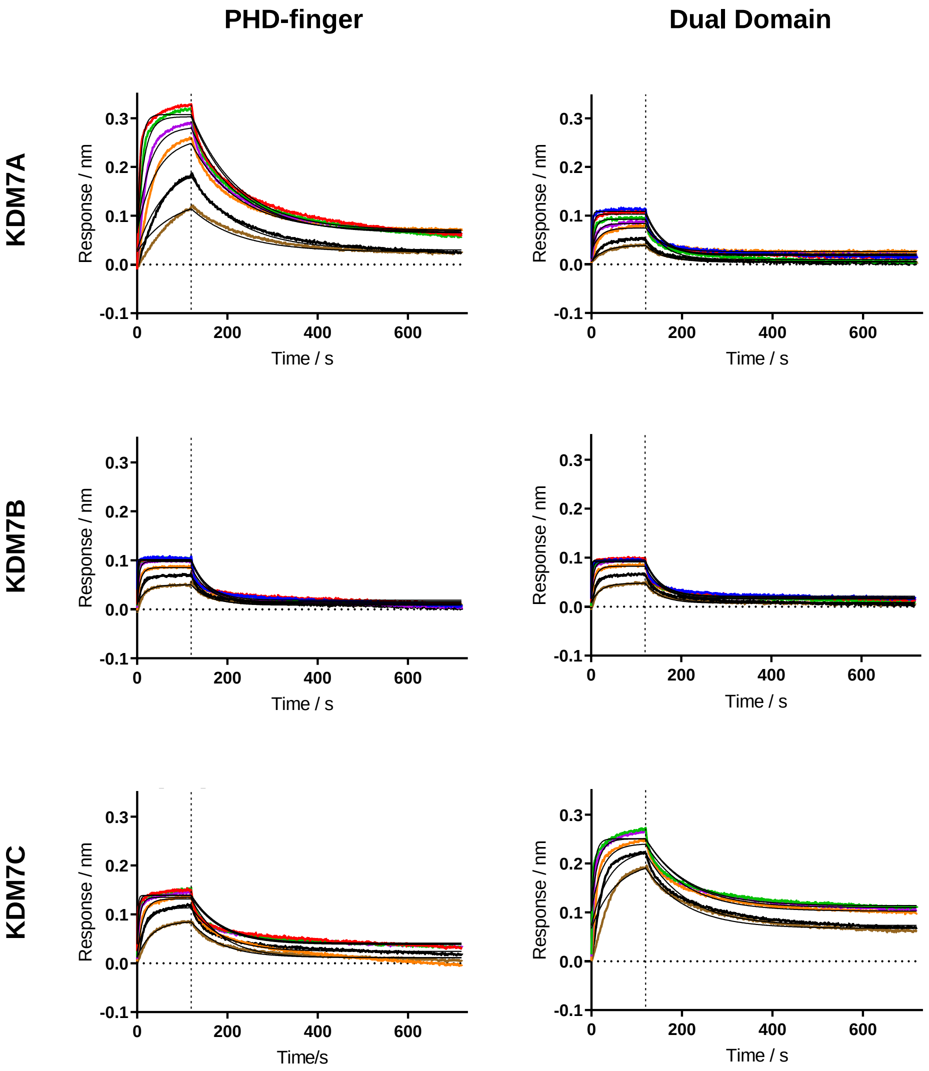

Figure S4. BLI for KDM7 proteins with H3$_{1\text{-}21}$K4me3.

BLI traces for binding to KDM7 protein domains immobilised by *C*-terminal His-tag on Ni-NTA biosensors, against H3$_{1\text{-}21}$K4me3 peptide. Response trace for each peptide concentration is represented by a different colour, with the fitted curves overlaid in black. See Figure S5 for results of fit and the top concentration plotted, with 2-fold dilutions thereafter. H3$_{1\text{-}21}$K4me3 sequence: ART[Kme3]QTARKSTGGKAPRKQLA with free *N*-terminus and primary amide *C*-terminus. Dual domain = PHD+JmjC.

Peptide	Protein	BLI K_D / M	pK_D	k_{on} x10^5 / M^{-1} s^{-1}	k_{off} x10^{-3} / s^{-1}	$t_{1/2}$ / mins	Global Fit R^2	Highest [Plotted] / nM
OC9	KDM7A PHD	1.90E-08 ± 2E-10	7.7	3.58 ± 0.03	6.79 ± 0.02	1.7	0.98	500
	KDM7B PHD	3.96E-09 ± 3E-11	8.4	3.60 ± 0.02	1.43 ± 0.01	8	0.98	250
	KDM7C PHD	1.05E-09 ± 1E-11	9.0	4.63 ± 0.02	0.483 ± 0.002	24	0.98	1000
	KDM7A Dual	4.25E-08 ± 5E-10	7.4	2.89 ± 0.03	12.3 ± 0.1	0.9	0.98	1000
	KDM7B Dual	5.98E-09 ± 1E-11	8.2	6.91 ± 0.07	4.13 ± 0.03	2.8	0.98	500
	KDM7C Dual	3.34E-10 ± 4E-12	9.5	4.09 ± 0.02	0.137 ± 0.001	84	0.96	1000
	KDM7B JmjC	> 1E-06	< 6.0	-	-	-	-	1000
	DIDO PHD	> 1E-06	< 6.0	-	-	-	-	5000
	TAF3 PHD	> 1E-06	< 6.0	-	-	-	-	1000
	ING2 PHD	> 1E-06	< 6.0	-	-	-	-	5000
	SPIN1 TTD	> 1E-06	< 6.0	-	-	-	-	5000
$H3_{1-21}$ K4me3	KDM7A PHD	4.42E-08 ± 1E-10	7.4	2.15 ± 0.02	9.51 ± 0.03	1.2	0.98	500
	KDM7B PHD	8.29E-08 ± 1.3E-09	7.1	3.18 ± 0.05	26.4 ± 0.1	0.4	0.97	5000
	KDM7C PHD	7.34E-08 ± 1.0E-09	7.1	2.02 ± 0.02	14.9 ± 0.1	0.8	0.97	2500
	KDM7A Dual	5.84E-08 ± 7E-10	7.2	4.60 ± 0.05	26.9 ± 0.1	0.4	0.98	1000
	KDM7B Dual	1.12E-07 ± 2E-09	7.0	2.52 ± 0.03	28.2 ± 0.1	0.4	0.98	5000
	KDM7C Dual	1.16E-07 ± 2E-09	6.9	0.946 ± 0.013	11.0 ± 0.1	1.0	0.96	1250
	DIDO PHD	1.26E-07 ± 2E-09	6.9	1.13 ± 0.02	14.2 ± 0.1	0.8	0.97	1250
	TAF3 PHD	6.04E-08 ± 6E-10	7.2	3.45 ± 0.03	20.8 ± 0.1	0.6	0.99	1000
	ING2 PHD	1.98E-06 ± 4E-08	5.7	1.12 ± 0.01	222 ± 1	0.1	0.99	5000
	SPIN1 TTD	8.97E-09 ± 2E-10	8.0	3.83 ± 0.04	3.44 ± 0.03	3.4	0.96	1250
OC3	KDM7B PHD	> 1E-06	< 6.0	-	-	-	-	1000
	KDM7B JmjC	> 1E-06	< 6.0	-	-	-	-	1000
	KDM7B Dual	2.95E-08 ± 3E-10	7.5	5.02 ± 0.05	14.8 ± 0.1	0.8	0.99	500
OC4	KDM7B PHD	> 1E-06	< 6.0	-	-	-	-	1000
	KDM7B JmjC	9.83E-08 ± 1.6E-09	7.0	2.49 ± 0.03	24.5 ± 0.1	0.5	0.98	500
	KDM7B Dual	5.86E-08 ± 9E-10	7.2	2.12 ± 0.02	12.4 ± 0.1	0.9	0.98	500

Figure S5. BLI data table for OC9, $H3_{1-21}$K4me3, OC3, OC4.

Results from fitted curves (1:1 BLI binding model, see methods for details) for screening of peptides against KDM7 and other protein domains. $t_{1/2}$ = half life (0.693 / K_{off})

KDM7A PHD

OC9

K_D = 33 ± 11 nM

pK_D = 7.5

KDM7B PHD

OC9

K_D = 12 ± 7 nM

pK_D = 7.9

KDM7C PHD

OC9

K_D = 10 ± 4 nM

pK_D = 8.0

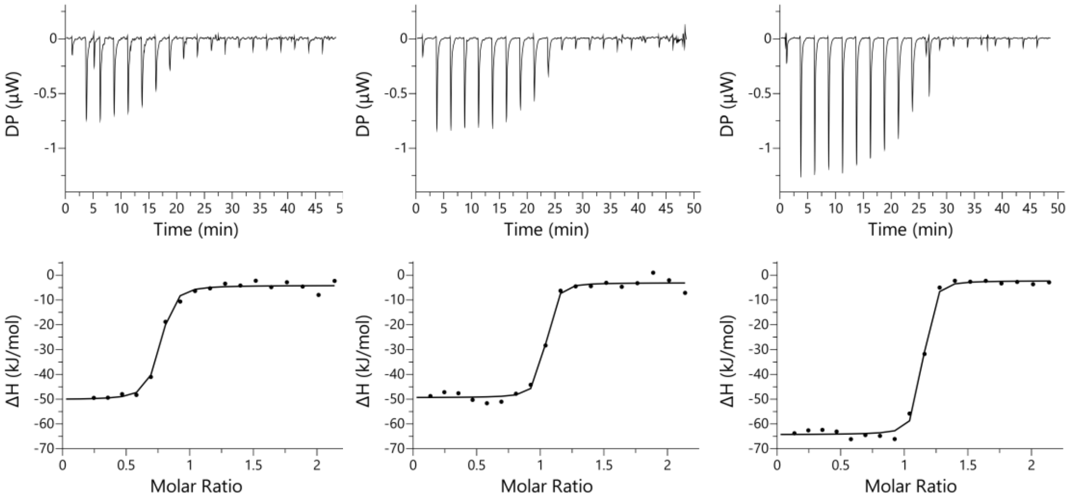

KDM7A Dual Domain

OC9

K_D = 34 ± 31 nM

pK_D = 7.5

KDM7B Dual Domain

OC9

K_D = 11 ± 6 nM

pK_D = 8.0

KDM7C Dual Domain

OC9

K_D = 10 ± 8 nM

pK_D = 8.0

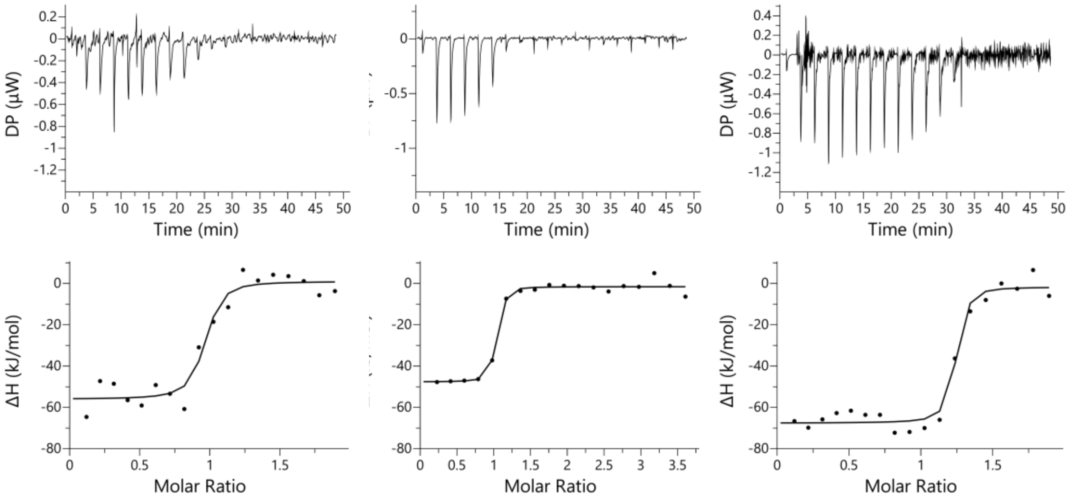

Figure S6. ITC titration curves for OC9 against KDM7 protein domains.

Top panels are raw heat of injection, bottom panels are normalised data fitted to 1:1 binding model. OC9 with KDM7A/B/C PHD-finger and dual domain proteins. For results of fit and conditions see Figure S10.

KDM7A PHD

$H3_{1-21}K4me3$

$K_D = 448 \pm 182$ nM

$pK_D = 6.3$

KDM7B PHD

$H3_{1-21}K4me3$

$K_D = 205 \pm 46$ nM

$pK_D = 6.7$

KDM7C PHD

$H3_{1-21}K4me3$

$K_D = 81 \pm 19$ nM

$pK_D = 7.1$

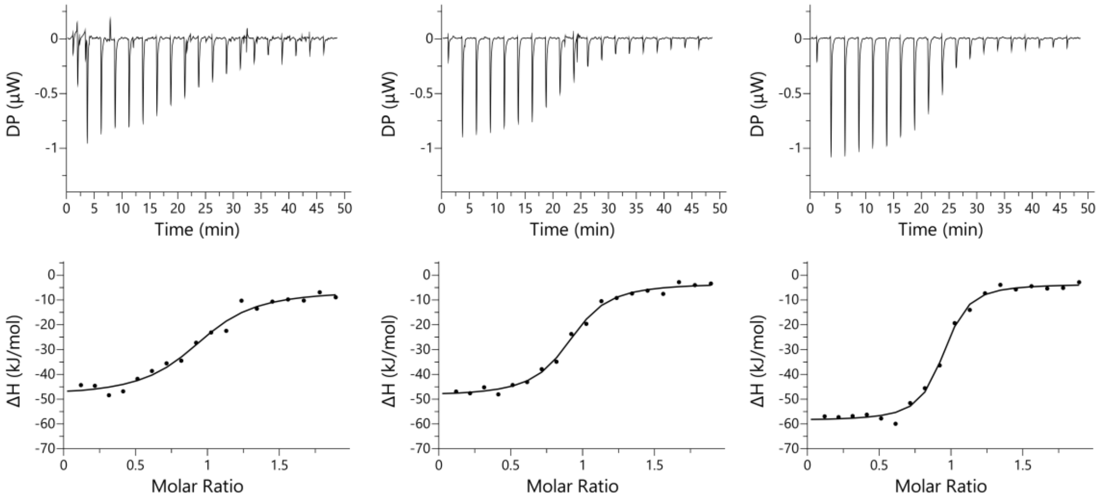

KDM7A Dual Domain

$H3_{1-21}K4me3$

$K_D = 51 \pm 27$ nM

$pK_D = 7.3$

KDM7B Dual Domain

$H3_{1-21}K4me3$

$K_D = 115 \pm 42$ nM

$pK_D = 6.9$

KDM7C Dual Domain

$H3_{1-21}K4me3$

$K_D = 601 \pm 167$ nM

$pK_D = 6.2$

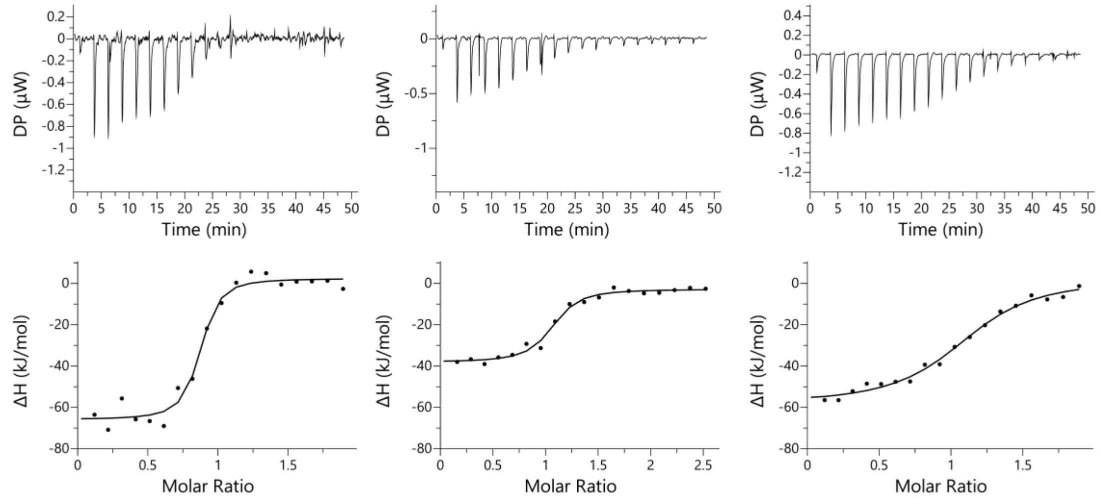

Figure S7. ITC titration curves for $H3_{1-21}K4me3$ against KDM7 protein domains.

Top panels are raw heat of injection, bottom panels are normalised data fitted to 1:1 binding model. $H3_{1-21}K4me3$ with KDM7A/B/C PHD-finger and dual domain proteins. For results of fit and conditions see Figure S10.

HisTrx-KDM7B PHD

OC9

$K_D = 8 \pm 4$ nM

$pK_D = 8.1$

HisTrx-KDM7B PHD

$H3_{1\text{-}21}K4me3$

$K_D = 141 \pm 37$ nM

$pK_D = 6.9$

HisTrx-KDM7B PHD

$H3_{1\text{-}21}$

K_D -

pK_D -

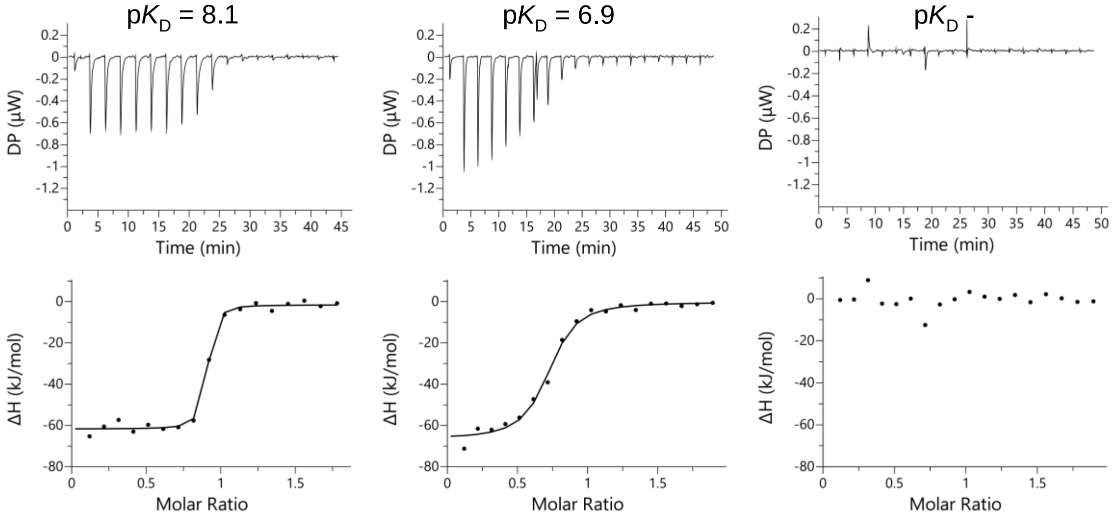

Figure S8. ITC titration curves for His-Trx-KDM7B PHD.

Top panels are raw heat of injection, bottom panels are normalised data fitted to 1:1 binding model. OC9, $H3_{1\text{-}21}K4me3$ and $H3_{1\text{-}21}$ with His-Trx tagged KDM7B PHD-finger. His-Trx = His_6+thioredoxin. For results of fit and conditions see Figure S10. The His-Trx tag made very little difference to binding of OC9 and $H3_{1\text{-}21}K4me3$.

KDM7B Dual Domain

OC3

$K_D = 163 \pm 91$ nM

$pK_D = 6.8$

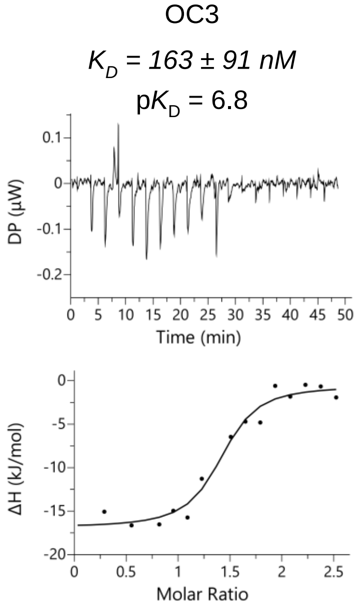

Figure S9. ITC titration curve for OC3.

Top panel is raw heat of injection, bottom panel is normalised data fitted to 1:1 binding model. OC3 with KDM7B dual domain. For results of fit and conditions see Figure S10.

Peptide in Syringe	Protein in Cell	ITC K_D / M	pK_D	N	ΔH / kJ mol^{-1}	TΔS / kJ mol^{-1}	ΔG / kJ mol^{-1}	Syringe / M	Protein / M
OC9	KDM7A PHD	3.34E-08 ± 1.11E-08	7.5	0.71 ± 0.01	-46.0 ± 1.3	-3.3	-42.7	1.13E-04	1.00E-05
	KDM7B PHD	1.17E-08 ± 6.73E-09	7.9	0.99 ± 0.01	-46.2 ± 1.2	-0.9	-45.3	1.13E-04	1.00E-05
	KDM7C PHD	9.56E-09 ± 3.53E-09	8.0	1.09 ± 0.01	-62.0 ± 1.0	-16.1	-45.8	1.13E-04	1.00E-05
	KDM7A Dual	3.43E-08 ± 3.12E-08	7.5	0.92 ± 0.03	-56.9 ± 4.3	-14.2	-42.7	1.00E-04	1.00E-05
	KDM7B Dual	1.05E-08 ± 6.34E-09	8.0	0.95 ± 0.02	-46.0 ± 1.5	-0.4	-45.6	1.13E-04	5.90E-06
	KDM7C Dual - ZB	9.73E-09 ± 8.05E-09	8.0	1.19 ± 0.01	-65.7 ± 3.1	-19.9	-45.8	1.00E-04	1.00E-05
	HisTrx-KDM7B PHD	8.27E-09 ± 4.15E-09	8.1	0.86 ± 0.01	-60.0 ± 1.3	-13.9	-46.2	1.00E-04	1.00E-05
	HisTrx-DIDO PHD	-	-	-	-	-	-	1.00E-04	1.00E-05
	KDM5A PHD3 - GST	-	-	-	-	-	-	5.00E-05	4.60E-06
$H3_{1-21}$ K4me3	KDM7A PHD	4.48E-07 ± 1.82E-07	6.3	0.93 ± 0.04	-42.7 ± 3.6	-6.4	-36.3	1.00E-04	1.00E-05
	KDM7B PHD	2.05E-07 ± 4.56E-08	6.7	0.89 ± 0.02	-45.6 ± 1.5	-7.4	-38.2	1.00E-04	1.00E-05
	KDM7C PHD	8.09E-08 ± 1.88E-08	7.1	0.90 ± 0.01	-55.1 ± 1.4	-14.6	-40.5	1.00E-04	1.00E-05
	KDM7A Dual	5.14E-08 ± 2.68E-08	7.3	0.83 ± 0.02	-68.3 ± 3.3	-26.6	-41.7	1.00E-04	1.00E-05
	KDM7B Dual	1.15E-07 ± 4.23E-08	6.9	1.02 ± 0.02	-35.3 ± 1.6	4.3	-39.7	1.00E-04	7.50E-06
	KDM7C Dual - ZB	6.01E-07 ± 1.67E-07	6.2	1.10 ± 0.04	-60.3 ± 4.2	-24.8	-35.6	1.00E-04	1.00E-05
	HisTrx-KDM7B PHD	1.41E-07 ± 3.74E-08	6.9	0.69 ± 0.01	-66.3 ± 2.3	-27.1	-39.2	1.00E-04	1.00E-05
	HisTrx-DIDO PHD	5.73E-08 ± 1.39E-08	7.2	0.70 ± 0.01	-63.5 ± 1.5	-22.1	-41.4	1.00E-04	1.00E-05
	KDM5A PHD3 - GST	1.33E-07 ± 7.21E-08	6.9	0.76 ± 0.04	-56.4 ± 5.2	-17.1	-39.3	5.00E-05	4.00E-06
OC3	KDM7B Dual	1.63E-07 ± 9.10E-08	6.8	1.37 ± 0.05	-16.3 ± 1.5	22.5	-38.8	1.00E-04	7.50E-06

Figure S10. ITC results: Tabulated values from 1:1 binding model and conditions

ITC buffer = 50 mM HEPES, 100 mM NaCl, pH 7.4, 0.2 µm filtered), at 25 °C. Dual = PHD+JmjC. HisTrx = His_6+thioredoxin tag, GST = glutathione S-transferase tag, ZB = Z-basic tag. Syringe contained peptide solution in buffer. Cell contained protein solution in buffer.

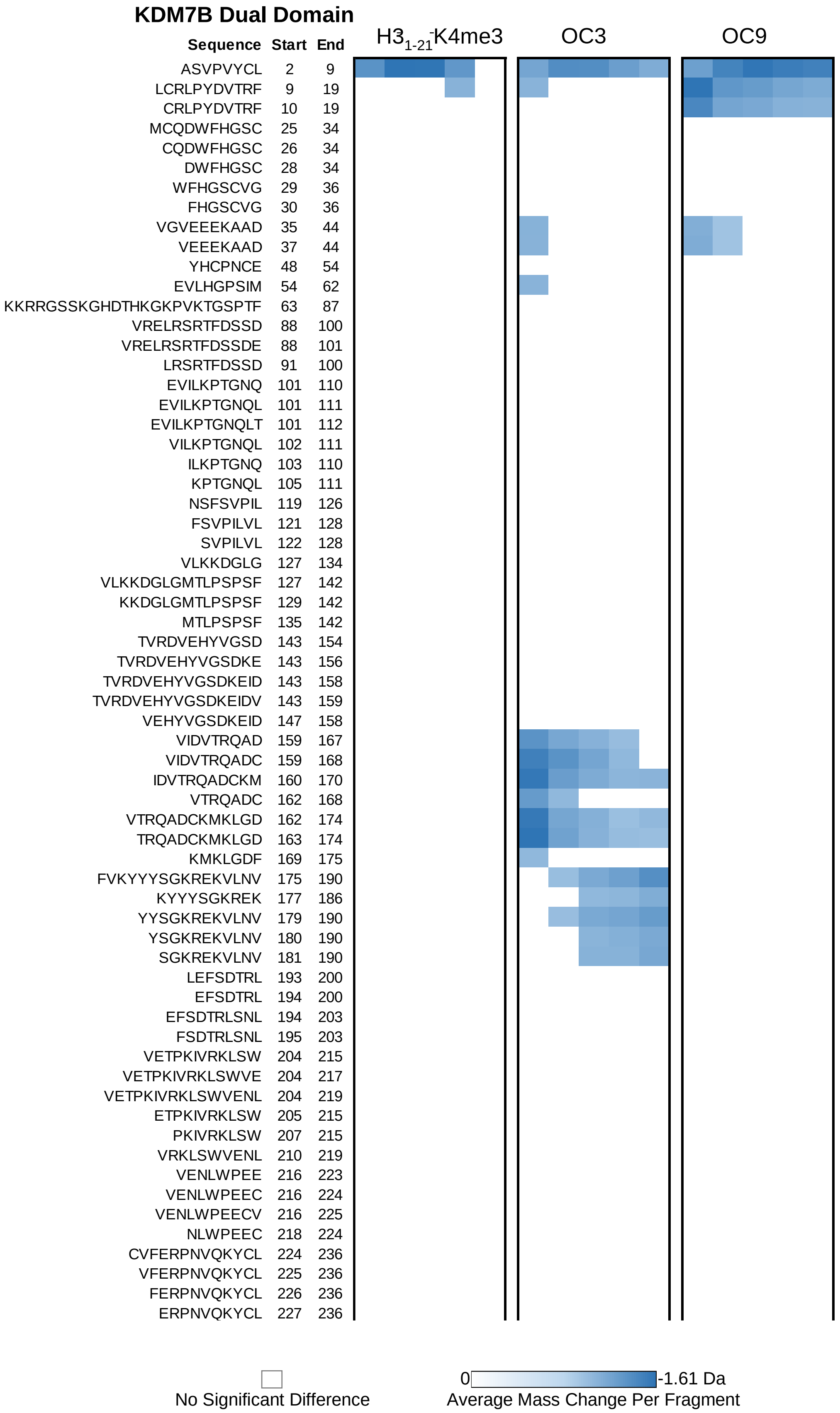

Figure S11A. HDX-MS peptide level view for KDM7B dual domain.

Peptide fragment level view of HDX-MS results from incubation of KDM7B dual domain with peptides. Mass changes are relative to a DMSO control. Continued in Figure S11B.

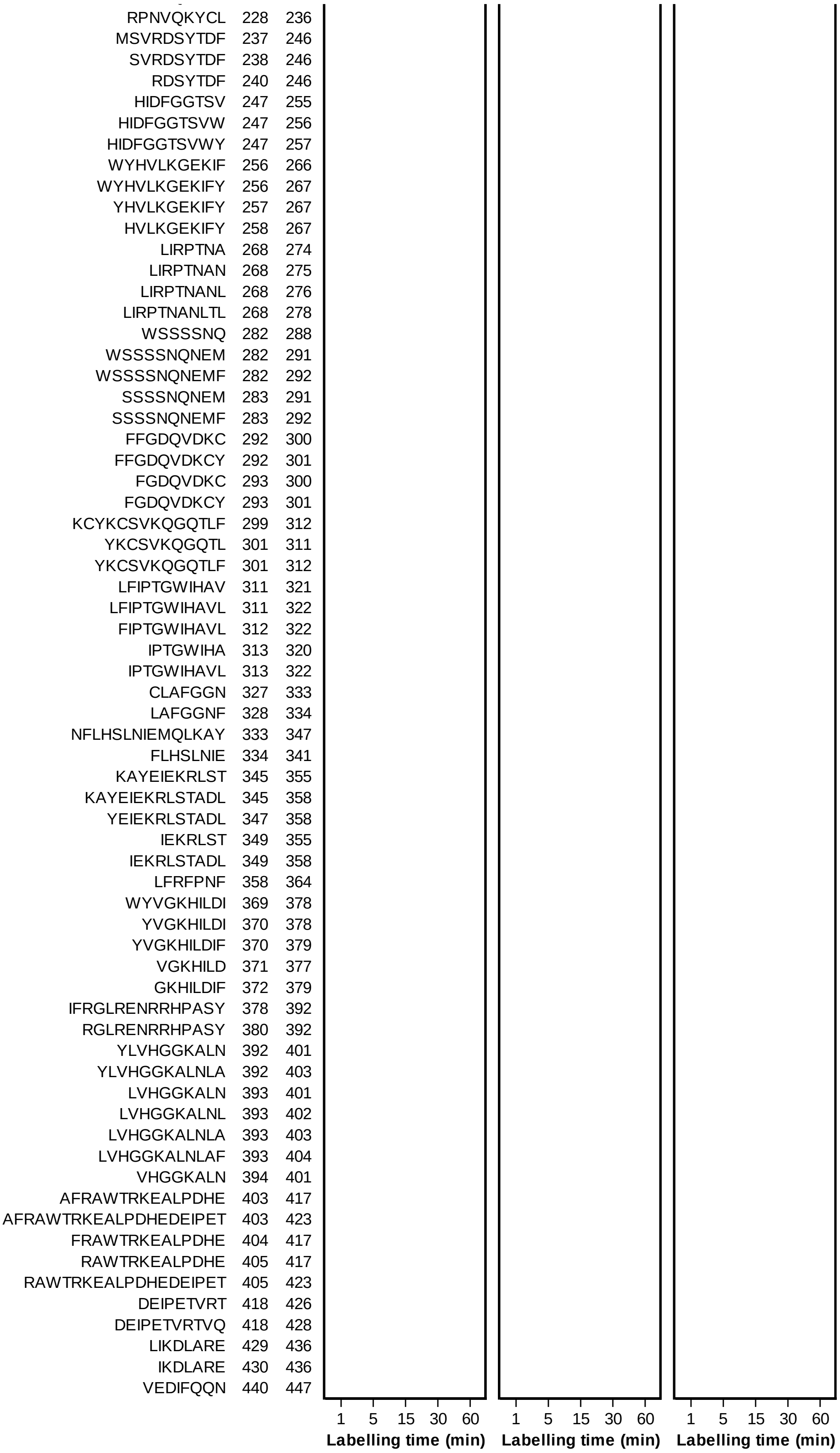

Figure S11B. Continuation of Figure S11A.

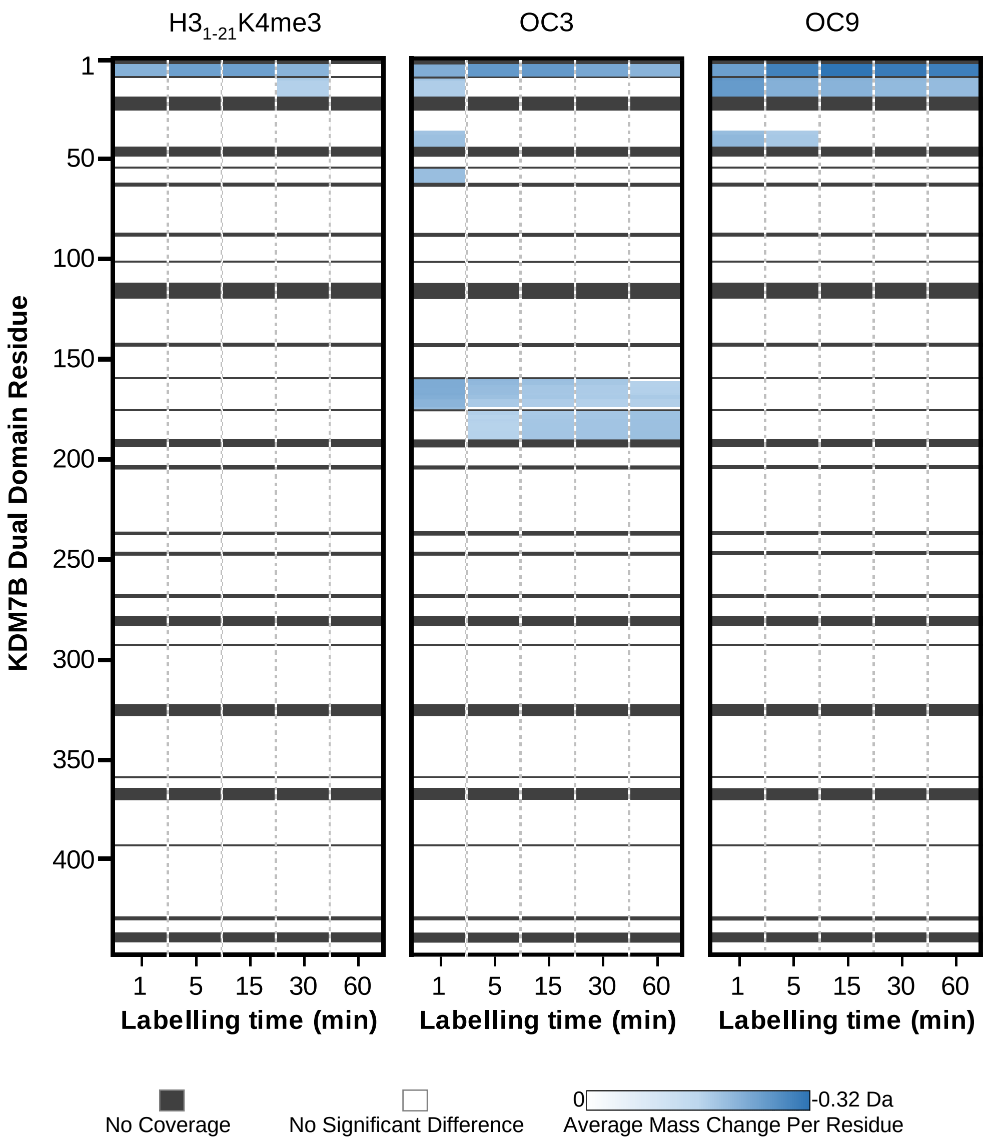

Figure S12. HDX-MS residue level view for KDM7B dual domain.

Residue level view generated from overlapping peptide fragments of HDX-MS results from incubation of KDM7B dual domain with compounds. Mass changes are relative to a DMSO control.

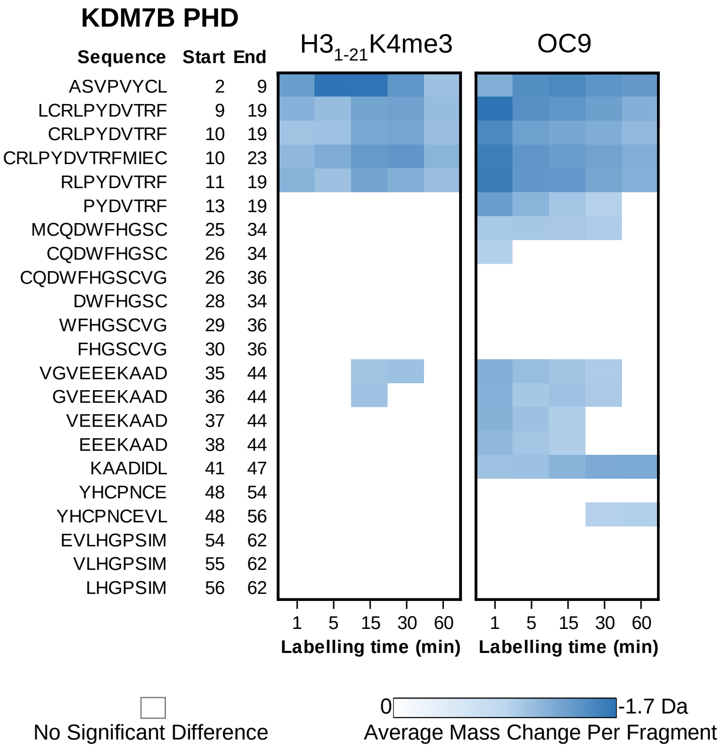

Figure S13. HDX-MS peptide level view for KDM7B PHD finger.

Peptide fragment level view of HDX-MS results from incubation of KDM7B PHD-finger with peptides. Mass changes are relative to a DMSO control.

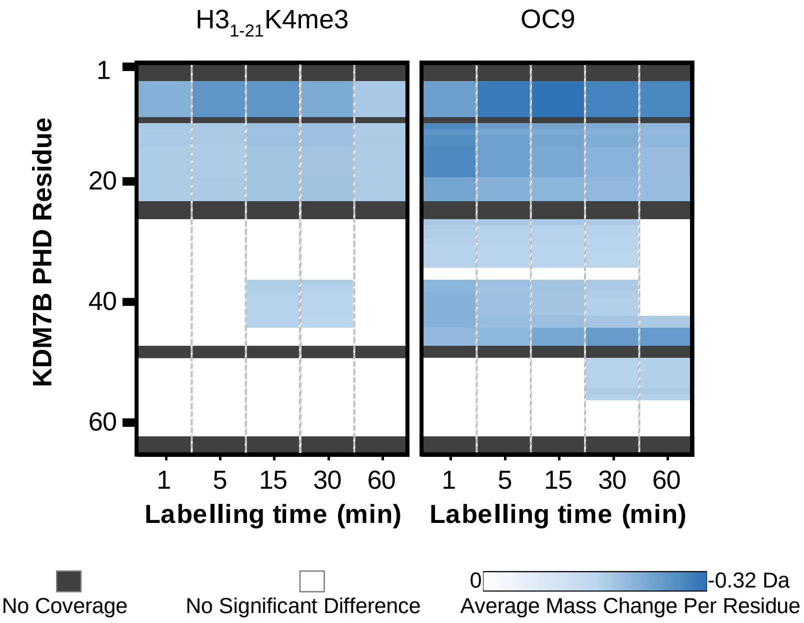

Figure S14. HDX-MS residue level view for KDM7B PHD finger.

Residue level view generated from overlapping peptide fragments of HDX-MS results from incubation of KDM7B PHD-finger with compounds. Mass changes are relative to a DMSO control.

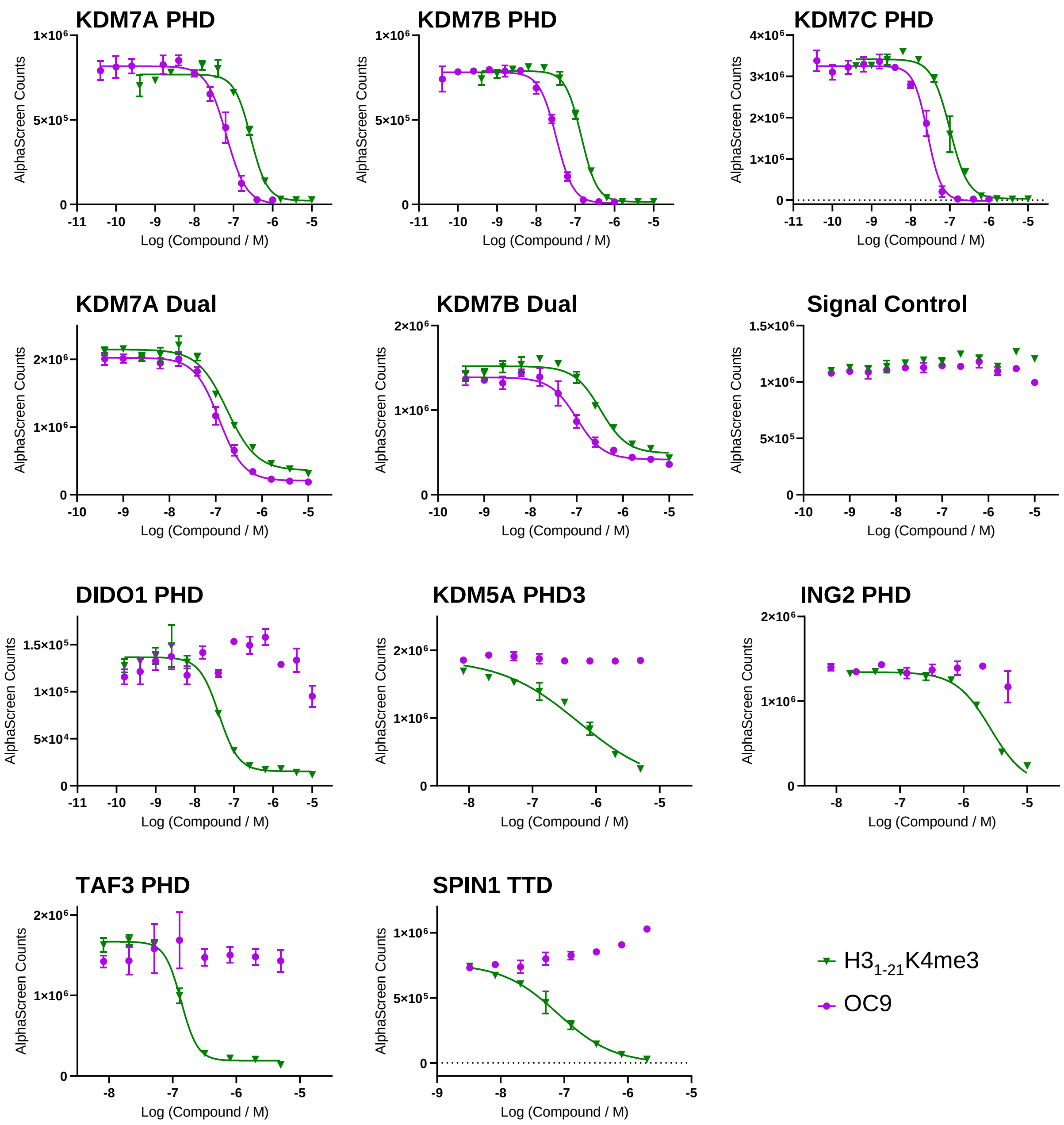

		KDM7A		KDM7B		KDM7C	DIDO	KDM5A	ING2	TAF3	SPIN1
		PHD	PHD+JmjC	PHD	PHD+JmjC	PHD	PHD	PHD3	PHD	PHD	TTD
OC9	IC_{50} (nM)	62	132	32	95	26	> 10,000	> 5000	> 5000	> 5000	> 2000
	pIC_{50} ± StDev	7.21 ± 0.04	6.88 ± 0.07	7.49 ± 0.01	7.02 ± 0.03	7.58 ± 0.01	< 5.0	< 5.3	< 5.3	< 5.3	< 5.7
H3$_{1-21}$ K4me3	IC_{50} (nM)	288	148	135	275	93	48	536	2660	136	79.4
	pIC_{50} ± StDev	6.54 ± 0.03	6.83 ± 0.11	6.87 ± 0.03	6.56 ± 0.03	7.03 ± 0.04	1.32 ± 0.04	6.3	5.6	6.9	7.1

Figure S15. AlphaScreen displacement for OC9 with Kme3-reader domains.

Representative examples of AlphaScreen assay measurements of H3$_{1-21}$K4me3-Bt peptide displacement by OC9 and H3$_{1-21}$K4me3 against a variety of H3K4me3 reader domains. The ‘signal control’ used a His$_6$-Biotin peptide to generate signal between beads, thus showing that neither compound interferes with the assay components themselves. Tabulated values are average IC_{50} and pIC_{50} of two independent experiments in duplicate. pIC_{50} in M. See methods for further details.

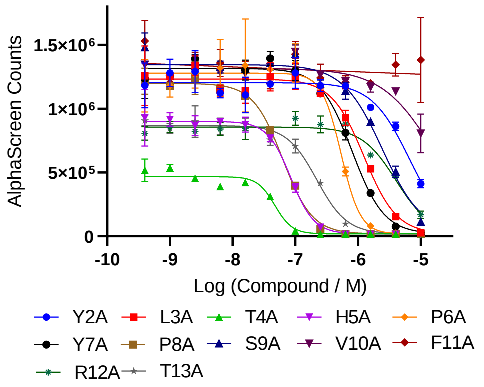

OC9 Variant	IC_{50} (nM)	pIC_{50} ± StDev
Y2A	7586	5.12 ± 0.09
L3A	1318	5.88 ± 0.03
T4A	32	7.50 ± 0.25
H5A	38	7.42 ± 0.49
P6A	490	6.31 ± 0.06
Y7A	851	6.07 ± 0.04
P8A	51	7.29 ± 0.13
S9A	2691	5.57 ± 0.06
V10A	> 10,000	> 5.0
F11A	> 10,000	> 5.0
R12A	3890	5.41 ± 0.02
T13A	200	6.70 ± 0.06

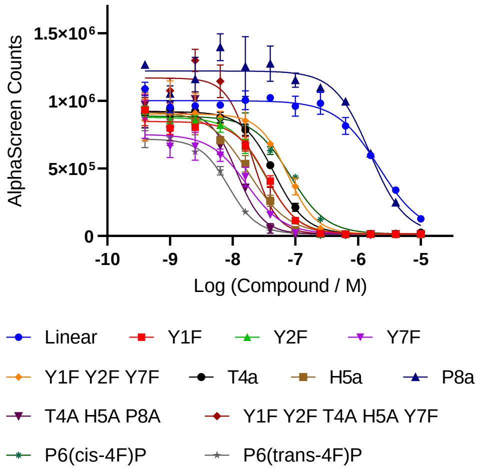

OC9 Variant	IC_{50} (nM)	pIC_{50} ± StDev
Linear	2199	5.66 ± 0.01
Y1F	28	7.55 ± 0.13
Y2F	24	7.62 ± 0.19
Y7F	14	7.86 ± 0.12
Y1F, Y2F, Y7F	65	7.19 ± 0.09
T4a	45	7.34 ± 0.00
H5a	20	7.70 ± 0.08
P8a	1700	5.77 ± 0.06
T4A, H5A, P8A	8	8.08 ± 0.20
Y1F, Y2F, T4A, H5A, Y7F	20	7.71 ± 0.01
P6(cis-4F)P	94	7.03 ± 0.09
P6(trans-4F)P	10	7.99 ± 0.11

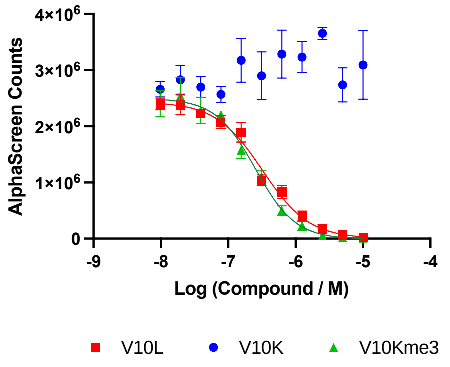

OC9 Variant	IC_{50} (nM)	pIC_{50} ± StDev
V10L	316	6.50 ± 0.13
V10K	>10,000	< 5.0
V10Kme3	234	6.63 ± 0.17

Figure S16. AlphaScreen displacement for OC9 variants with KDM7B.

Representative examples of AlphaScreen assay measurements of $H3_{1\text{-}21}$K4me3-Bt peptide displacement from KDM7B PHD-finger protein by OC9 variant peptides. Tabulated values are average IC_{50} and pIC_{50} of at least two independent experiments in duplicate. pIC_{50} in M. ‘a’ = D-alanine.

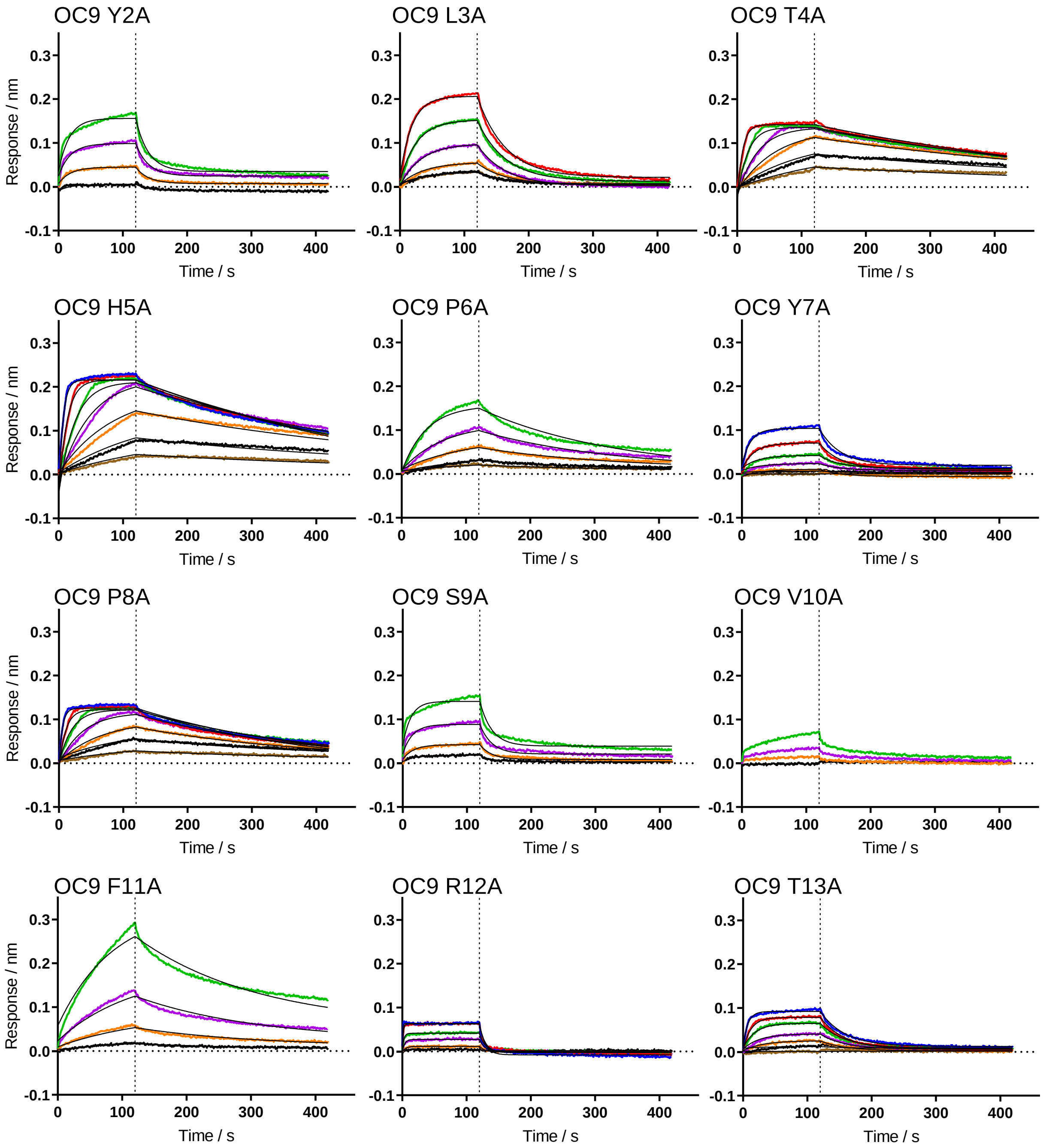

Figure S17. BLI for KDM7B PHD-finger protein with OC9 alanine scan variants.

BLI traces for binding to KDM7B PHD-finger with *C*-terminal his-tag immobilised on Ni-NTA biosensors. Response trace for each peptide concentration is represented by a different colour, with fitted curve overlaid in black. See Figure S19 for results of fit and the top concentration plotted, with 2-fold dilutions thereafter.

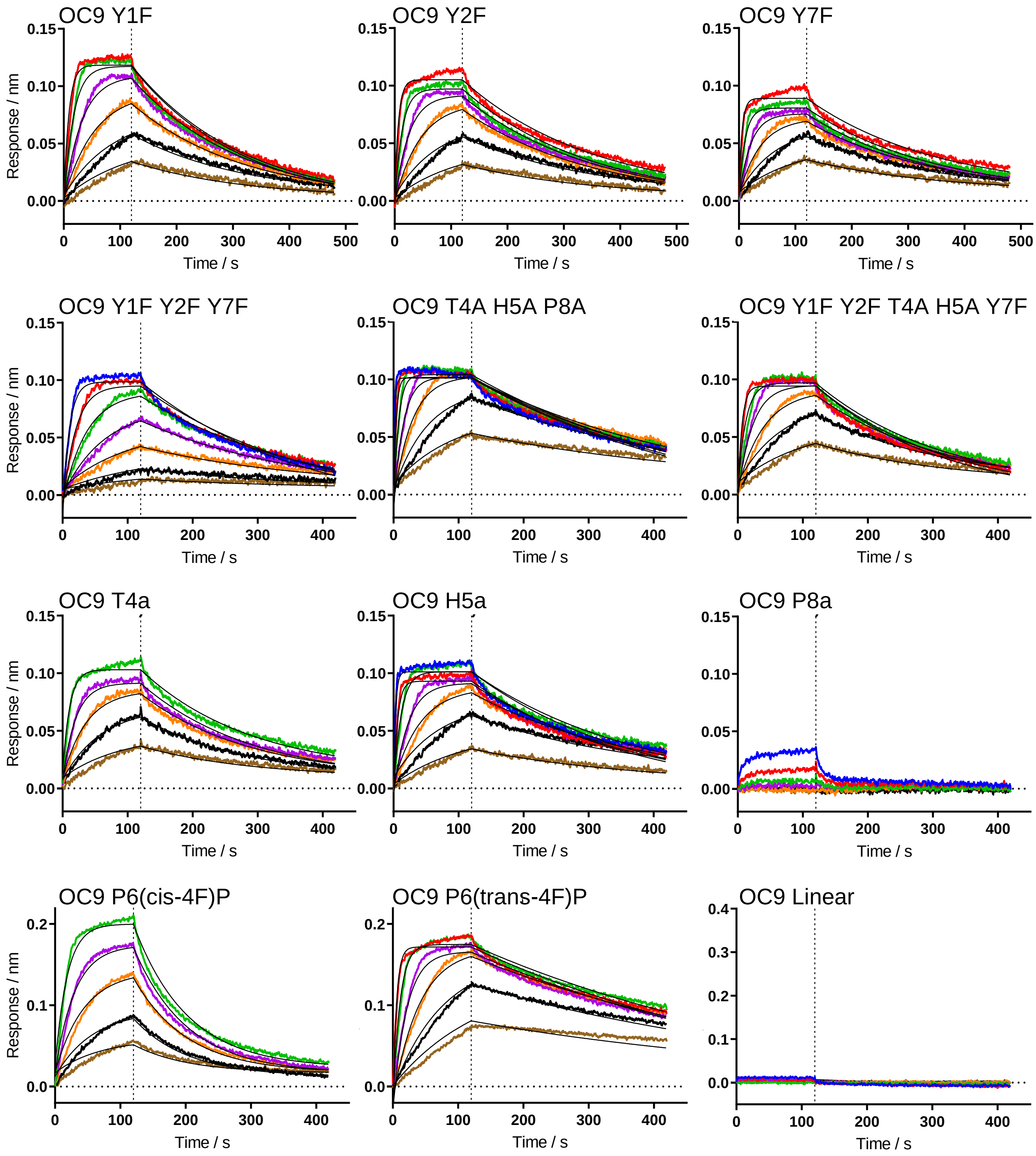

Figure S18A. BLI for KDM7B PHD-finger protein with OC9 substitution variants.

BLI traces for binding to KDM7B PHD-finger with *C*-terminal his-tag immobilised on Ni-NTA biosensors. Response trace for each peptide concentration is represented by a different colour, with fitted curve overlaid in black. See Figure S19 for results of fit and the top concentration plotted, with 2-fold dilutions thereafter.

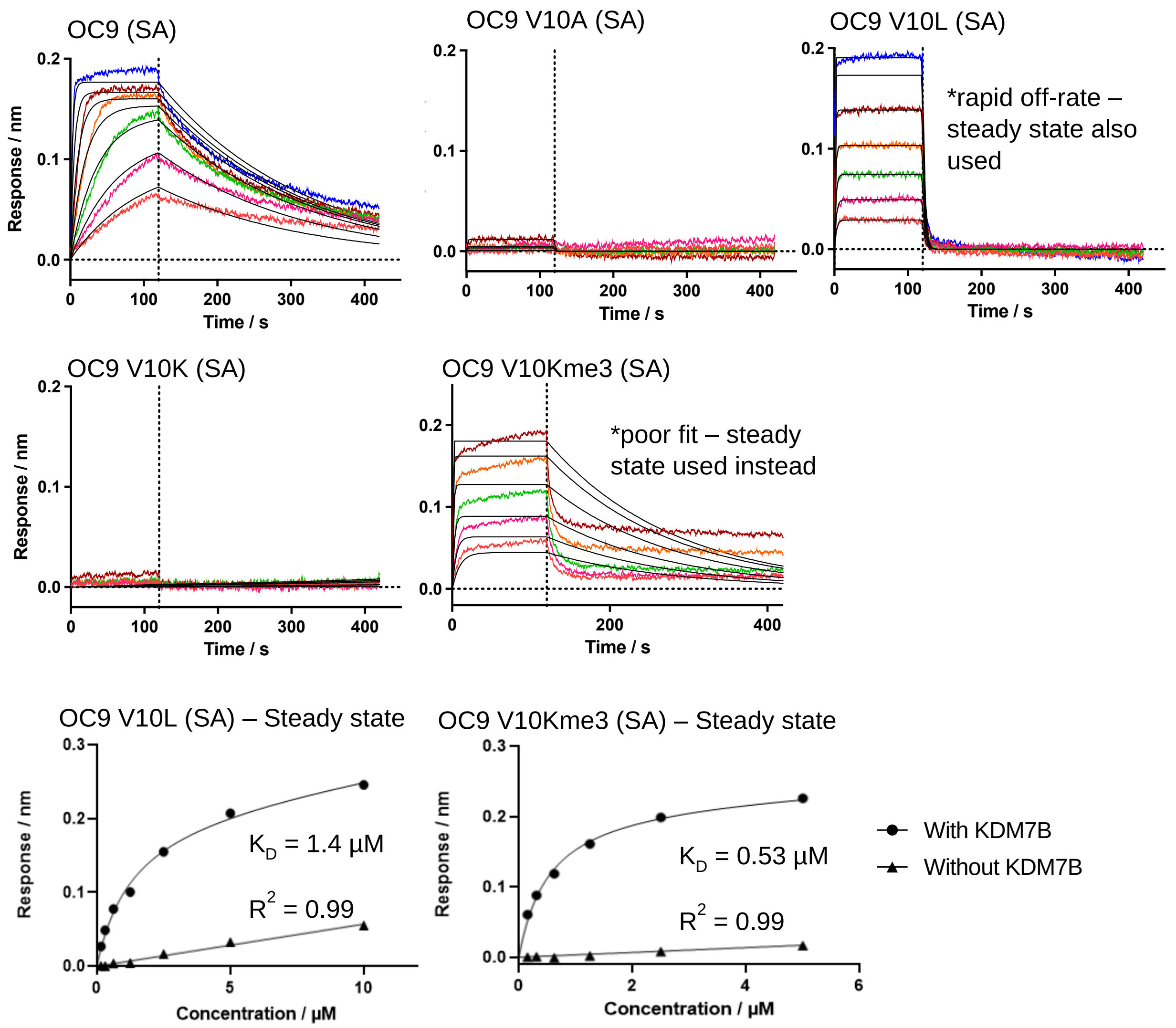

Figure S18B. BLI for KDM7B PHD-finger protein with OC9 substitution variants.

BLI traces for binding to immobilised KDM7B dual domain (PHD+JmjC) with *C*-terminal Avitag-biotin immobilised on streptavidin biosensors (SA). Response trace for each peptide concentration is represented by a different colour, with fitted curve overlaid in black. See Figure S19 for results of fit and the top concentration plotted, with 2-fold dilutions thereafter.

Protein	OC9 Variant	BLI K_D / M	pK_D	k_{on} x10^5 / M^{-1} s^{-1}	k_{off} x10^{-3} / s^{-1}	$t_{1/2}$ / mins	T_r / mins	Global Fit R^2	Bio-sensor	Highest [Plotted] / nM
KDM7B PHD	OC9	3.96E-09 ± 3E-11	8.4	3.60 ± 0.02	1.43 ± 0.01	8.1	11.7	0.98	Ni-NTA	250
	OC9 Y2A	1.33E-05 ± 1E-06	4.9	0.038 ± 0.003	50.8 ± 0.3	0.2	0.3	0.99	Ni-NTA	2500
	OC9 L3A	3.60E-07 ± 3E-09	6.4	0.631 ± 0.005	22.7 ± 0.5	0.5	0.7	0.99	Ni-NTA	500
	OC9 T4A	7.03E-09 ± 7E-11	8.2	2.48 ± 0.01	1.75 ± 0.01	6.6	9.5	0.99	Ni-NTA	500
	OC9 H5A	1.34E-08 ± 1E-10	7.9	1.56 ± 0.01	2.08 ± 0.01	5.5	8.0	0.98	Ni-NTA	1000
	OC9 P6A	5.41E-08 ± 4E-10	7.3	0.867 ± 0.013	4.69 ± 0.03	2.5	3.6	0.98	Ni-NTA	250
	OC9 Y7A	6.98E-07 ± 1E-08	6.2	0.436 ± 0.001	30.4 ± 0.1	0.4	0.5	0.99	Ni-NTA	1000
	OC9 P8A	1.88E-08 ± 2E-10	7.7	1.88 ± 0.01	3.53 ± 0.01	3.3	4.7	0.98	Ni-NTA	1000
	OC9 S9A	5.24E-06 ± 3E-07	5.3	0.103 ± 0.005	53.7 ± 0.1	0.2	0.3	0.98	Ni-NTA	2500
	OC9 V10A	> 1E-06	< 6.0	-	-	-	-	-	Ni-NTA	2500
	OC9 F11A	2.01E-06 ± 1E-07	5.7	0.0262 ± 0.0012	5.25 ± 0.04	2.2	3.2	0.99	Ni-NTA	2500
	OC9 R12A	9.31E-07 ± 2E-08	6.0	1.22 ± 0.03	114 ± 1	0.1	0.1	0.99	Ni-NTA	10000
	OC9 T13A	2.44E-07 ± 3E-09	6.6	1.07 ± 0.01	26.3 ± 0.1	0.4	0.6	0.99	Ni-NTA	1000
	OC9 Linear	> 1E-06	< 6.0	-	-	-	-	-	Ni-NTA	1000
	OC9 Y1F	2.31E-08 ± 3E-10	7.6	2.18 ± 0.02	5.03 ± 0.02	2.3	3.3	0.98	Ni-NTA	500
	OC9 Y2F	1.48E-08 ± 2E-10	7.8	2.89 ± 0.02	4.29 ± 0.02	2.7	3.9	0.98	Ni-NTA	500
	OC9 Y7F	1.21E-08 ± 1E-10	7.9	3.55 ± 0.03	4.31 ± 0.03	2.7	3.9	0.98	Ni-NTA	500
	OC9 Y1F Y2F Y7F	4.11E-08 ± 5E-10	7.4	0.946 ± 0.007	3.89 ± 0.02	3.0	4.3	0.98	Ni-NTA	1000
	OC9 T4A H5A P8A	5.09E-09 ± 7E-11	8.3	5.33 ± 0.04	2.71 ± 0.02	4.3	6.2	0.97	Ni-NTA	1000
	OC9 T4a	1.69E-08 ± 2E-10	7.8	3.48 ± 0.03	5.88 ± 0.03	2.0	2.8	0.98	Ni-NTA	250
	OC9 H5a	1.17E-08 ± 2E-10	7.9	3.32 ± 0.03	3.87 ± 0.03	3.0	4.3	0.97	Ni-NTA	1000
	OC9 P8a	> 1E-06	< 6.0	-	-	-	-	-	Ni-NTA	1000
	OC9 Y1F Y2F T4A H5A Y7F	1.58E-08 ± 2E-10	7.8	3.16 ± 0.03	4.99 ± 0.03	2.3	3.3	0.97	Ni-NTA	500
	OC9 P6cis-4-fluoro-P	7.21E-08 ± 9E-10	7.1	1.69 ± 0.02	12.2 ± 0.1	0.9	1.4	0.99	Ni-NTA	250
	OC9 P6trans-4-fluoro-P	4.92E-09 ± 5E-11	8.3	3.66 ± 0.02	1.80 ± 0.01	6.4	9.3	0.98	Ni-NTA	500
	OC9 V10$_D$V	> 1E-05	< 5.0	-	-	-	-	-	Ni-NTA	10000
KDM7B Dual	OC9	1.38E-08 ± 1E-10	7.9	3.69 ± 0.03	5.09 ± 0.01	2.3	3.3	0.96	SA	1000
	OC9 V10L	1.14E-06 ± 3E-08	5.9	3.00 ± 0.06	341 ± 2	0.03	0.05	0.99	SA	10000
	OC9 V10K	> 1E-05	< 5.0	-	-	-	-	-	SA	10000
	OC9 V10Kme3	5.33E-07	6.3	From steady state model				0.99	SA	2500
	OC9 V10A	> 1E-05	< 5.0	-	-	-	-	-	SA	10000
DIDO1 PHD	OC9	> 1E-06	< 6.0	-	-	-	-	-	Ni-NTA	5000
	OC9 Y1F Y2F Y7F	> 1E-06	< 6.0	-	-	-	-	-	Ni-NTA	5000
	OC9 T4A H5A P8A	> 1E-06	< 6.0	-	-	-	-	-	Ni-NTA	5000
	OC9 Y1F Y2F T4A H5A Y7F	> 1E-06	< 6.0	-	-	-	-	-	Ni-NTA	5000
	OC9 H5a	> 1E-06	< 6.0	-	-	-	-	-	Ni-NTA	5000

Figure S19. BLI data table for OC9 ala scan and variant peptides.

Tabulated values from 1:1 BLI binding models (see methods for details) for screening OC9 substitution variants against KDM7 and other proteins. $t_{1/2}$ = half life (0.693 / K_{off}), T_r = residence time (1 / K_{off}).

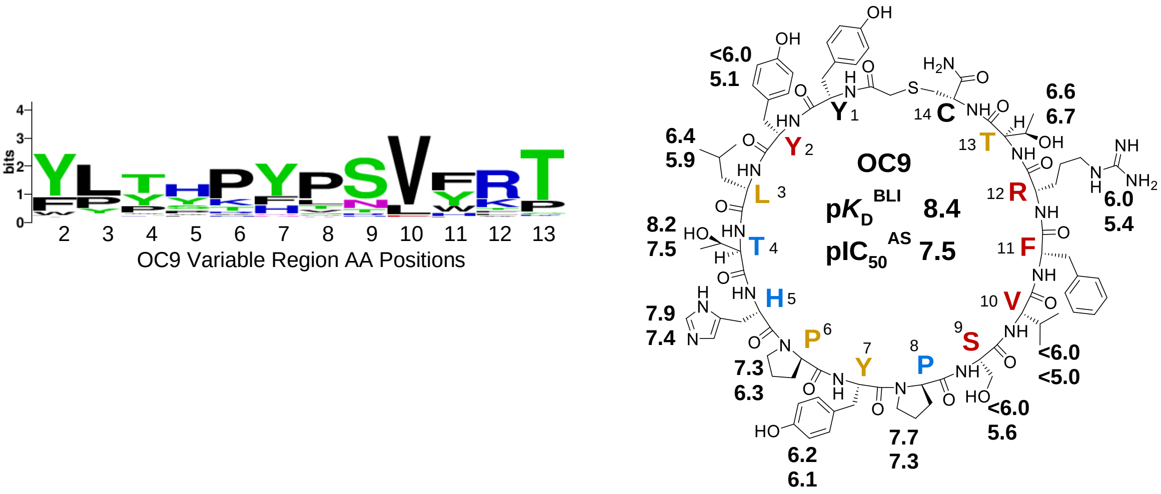

Figure S20. Bioinformatic analysis of NGS results comparison to OC9 SAR.

(**Left**) - Logo plot representation of variable region in OC9-related sequences from selection NGS. The 902 related sequences contained up to 6 differences from OC9 after ‘fuzzy’ matching was applied (S ≡ T, F ≡ H ≡ W ≡ Y, K ≡ R, D ≡ E ≡ N ≡ Q, A ≡ I ≡ L ≡ V). (**Right**) - Mapping of alanine scanning assay results to OC9 structure to indicate SAR.

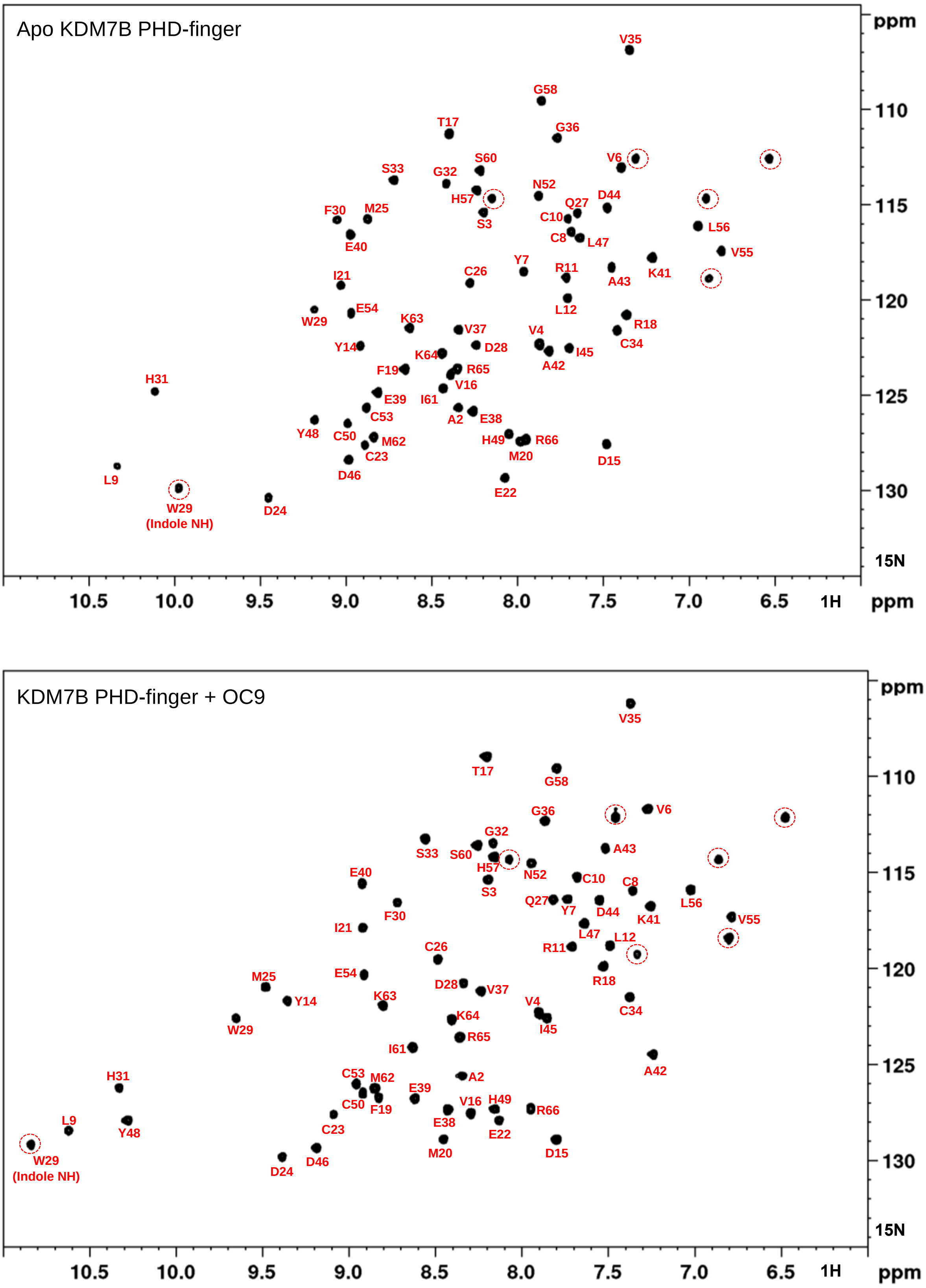

Figure S21. **^{1}H-^{15}N HSQC spectra of the KDM7B PHD-finger, apo and in complex with OC9.**

Annotated with the assigned backbone amide NH residues and the W29 indole. Red circles indicate side-chain associated NH. OC9:PHD is 1:1.

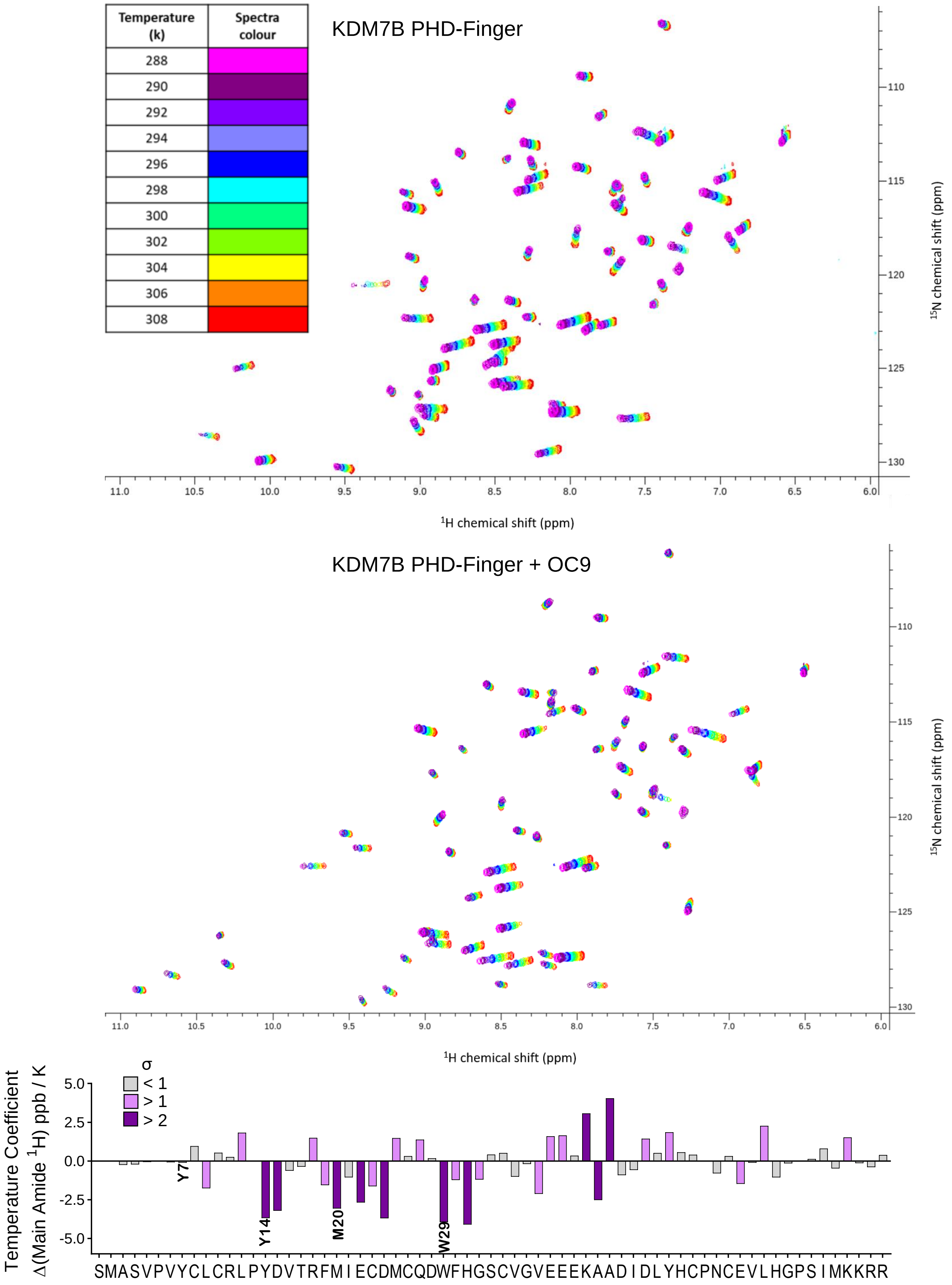

Figure S22. ^{1}H-^{15}N HSQC spectra temperature dependence of the KDM7B PHD-finger, apo and in complex with OC9.

The changes in ^{1}H NMR temperature coefficient for the backbone amide signals of KDM7B PHD-finger are graphed and cage residues are noted (bold). Significant values are indicated by colour (>1 s.d. (standard deviation) pink, >2 s.d. purple). OC9:PHD is 5:1.

Apo PHD-finger

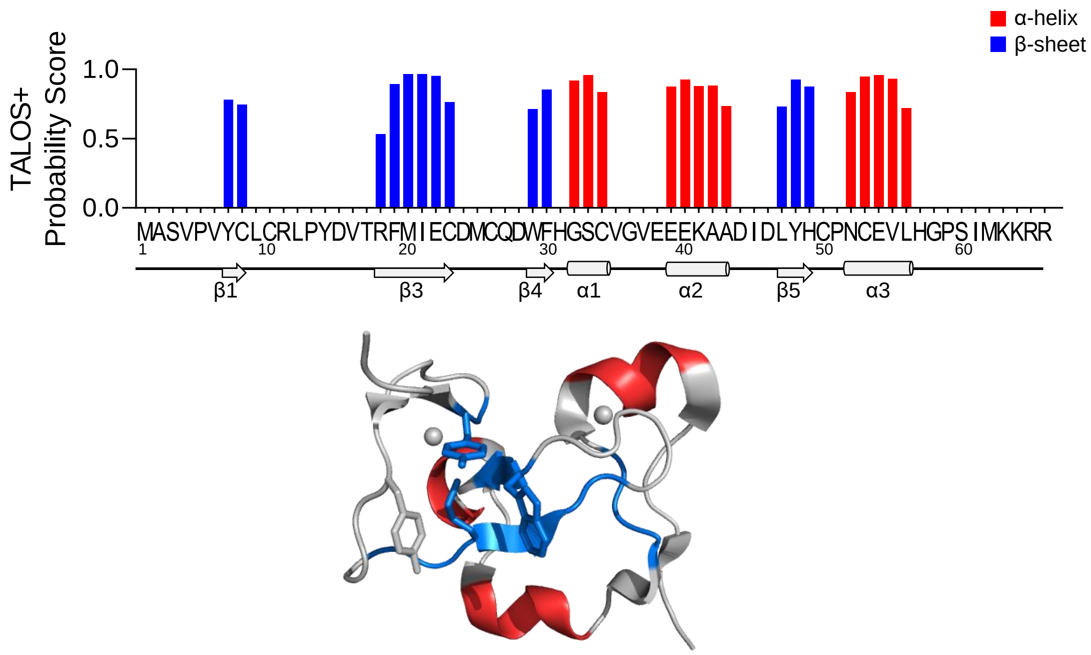

PHD-finger + OC9

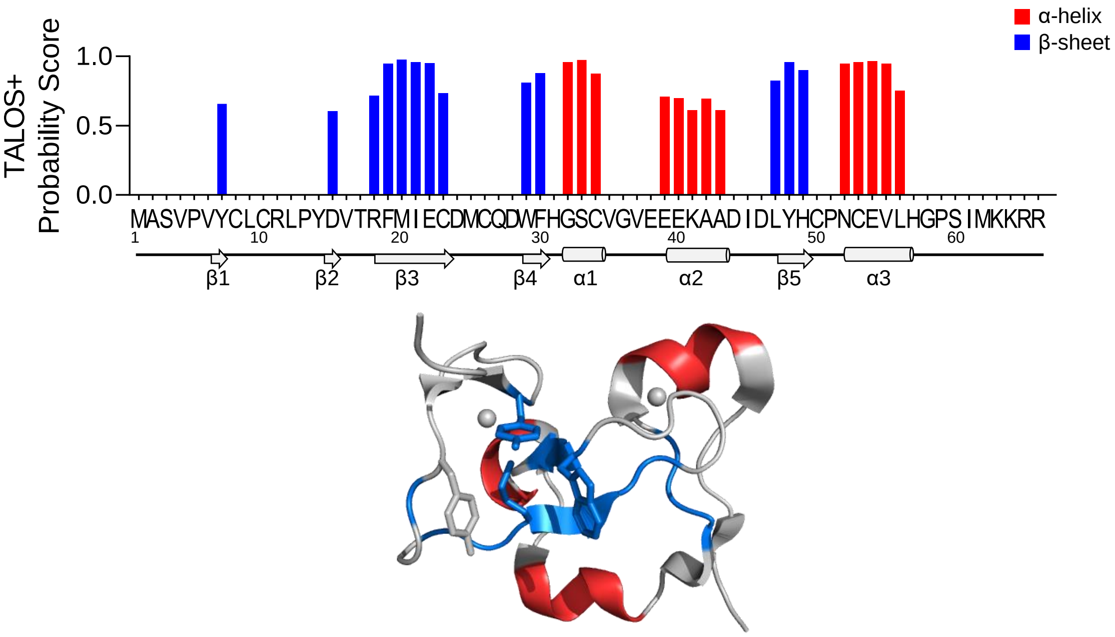

Figure S23. TALOS+ prediction of PHD-finger secondary structure elements

The TALOS+ software package was used to predict the likelihood of involvement in either alpha helix or beta sheet secondary structure elements for each residue in solution, based on their chemical shifts and sequence. A score of greater than 0.5 was considered probable. The data are colour mapped to the PDB structure 3KV4 for comparison. The cage residues' side chains are shown.

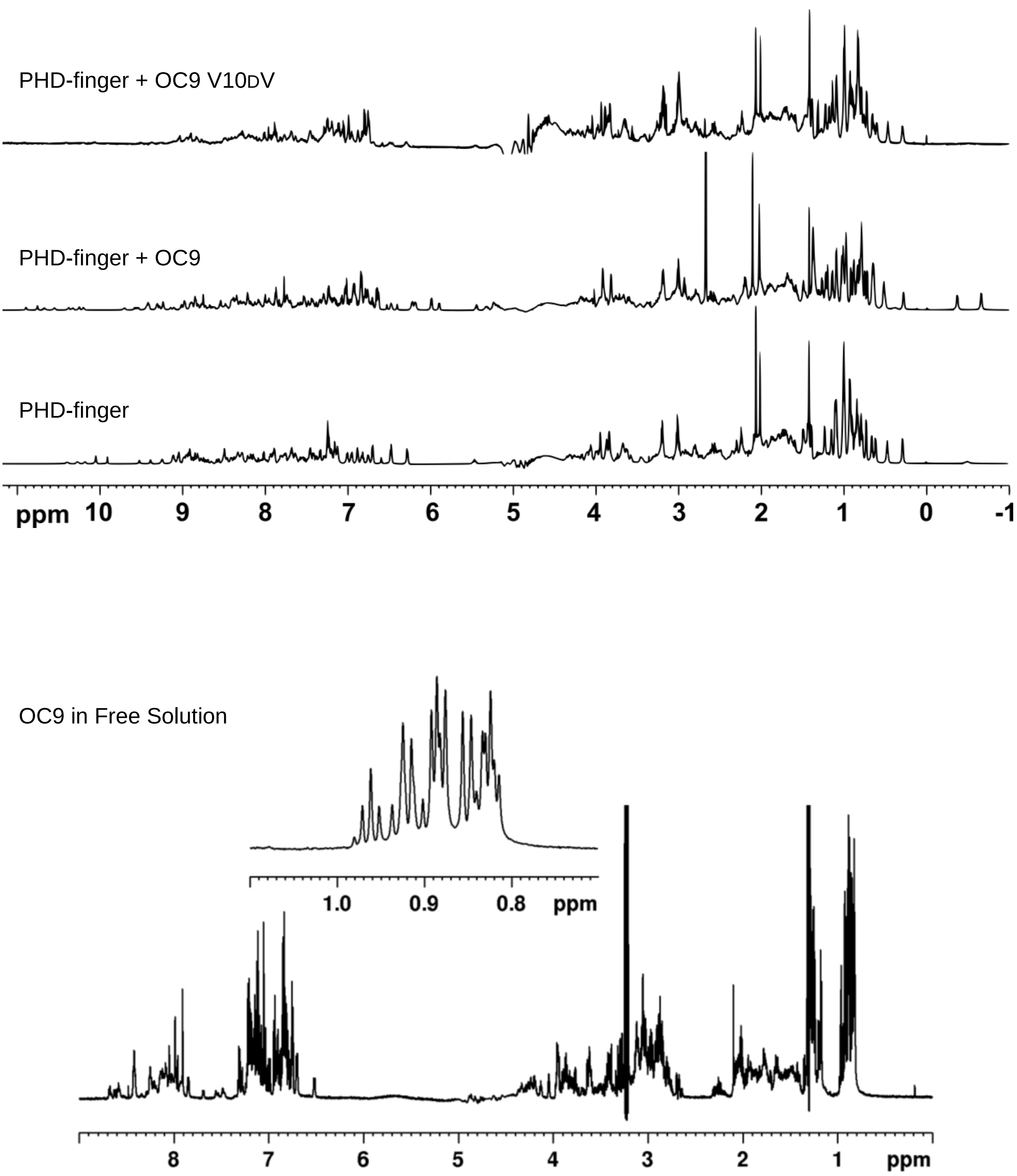

Figure S24A. 1D ^{1}H-NMR spectra of KDM7B PHD-finger, apo and complex with OC9 or OC9 V10DV, and free OC9

(Top): Stacked ^{1}H-NMR spectra for the free KDM7B PHD-finger in buffered solution, 1:1 with OC9 and 1:1 with OC9 V10DV. Changes are visible for the +OC9 spectrum at -0.5 ppm and 6 ppm, relative to free protein and +OC9 V10DV.

(Bottom): 1H-NMR signals for OC9 when free in buffer, not bound to protein. The complexity of the methyl region (corresponding to 1 Leu and 1 Val) evidences the multiple conformations observed for OC9.

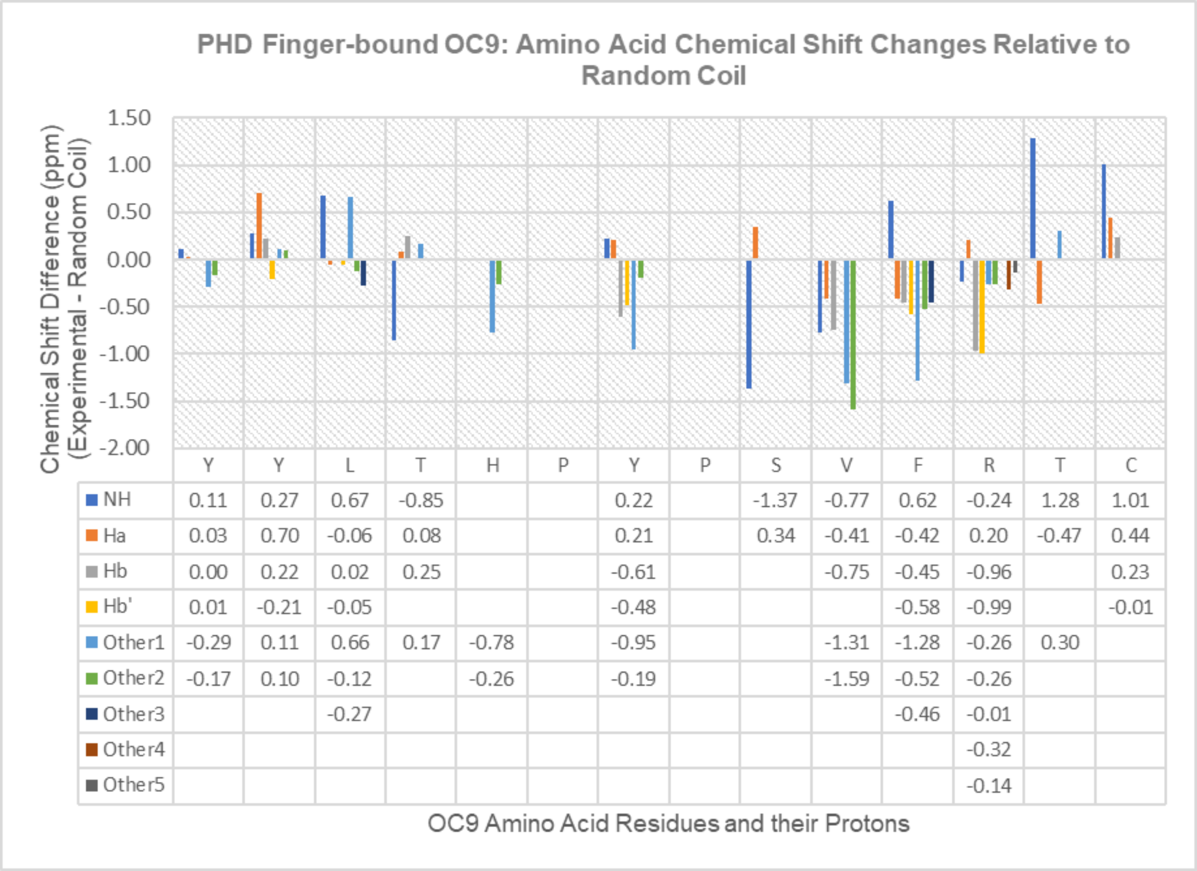

	1H Chemical Shift (ppm): OC9 (Complex)					1H Chemical Shift (ppm): Random coil				
**AA**	**NH**	**Hα**	**Hβ**	**Hβ'**	**Other**	**NH**	**Hα**	**Hβ**	**Hβ'**	**Other**
**Y1**	8.23	4.58	3.03	2.99	6.85 (o), 6.67 (m)	8.12	4.55	3.03	2.98	7.14 (o), 6.84 (m)
**Y2**	8.39	5.25	3.25	2.77	7.25 (o), 6.94 (m)	8.12	4.55	3.03	2.98	7.14 (o), 6.84 (m)
**L3**	8.83	4.28	1.64	1.57	2.25 (γ), 0.80 (δ), 0.65 (δ')	8.16	4.34	1.62	1.62	1.59 (γ), 0.92 (δ), 0.87 (δ')
**T4**	7.30	4.43	4.49		1.38 (γ Me)	8.15	4.35	4.24		1.21 (γ Me)
**H5**	ND	ND	ND	ND	7.79 (Hε), 7.03 (Hδ)	8.37	5.00	3.23	3.12	8.57 (Hε), 7.29 (Hδ)
**P6**		ND	ND	ND	ND		4.42	2.29	1.94	2.02 (γ), 2.02 (γ'), 3.63 (δ), 3.63 (δ')
**Y7**	8.32	5.05	2.44	2.41	6.20 (o), 6.67 (m)	8.10	4.84	3.05	2.89	7.15 (o), 6.86 (m)
**P8**		ND	ND	ND	ND		4.42	2.29	1.94	2.02 (γ), 2.02 (γ'), 3.63 (δ), 3.63 (δ')
**S9**	6.94	4.81	ND	ND		8.31	4.47	3.89	3.87	
**V10**	7.26	3.71	1.33		-0.37 (γ), -0.66 (γ')	8.03	4.12	2.08		0.94 (γ), 0.93 (γ')
**F11**	8.92	4.20	2.69	2.46	6.00 (o), 6.86 (m+p)	8.30	4.62	3.14	3.04	7.28 (o), 7.38 (m), 7.32 (p)
**R12**	7.99	4.54	0.90	0.77	1.37 (γ, γ'), 3.19 (δ), 2.88 (δ'), 7.93 (εNH)	8.23	4.34	1.86	1.76	1.63 (γ), 1.63 (γ'), 3.20 (δ), 3.20 (δ'), 8.07 (εNH)
**T13**	9.43	3.88	ND		1.51 (γ Me)	8.15	4.35	4.24		1.21 (γ Me)
**C14**	9.33	4.99	3.16	2.92		8.32	4.55	2.93	2.93	

Figure S24B. 1D ^{1}H-NMR chemical shifts for OC9 when complexed to KDM7B PHD-Finger

Chemical shift values for OC9 (determined via isotope filtering of the bound spectrum), showing the most substantially affected residues, compared to random coil amino acids [1] are the 'SVFR' motif. Note the random coil values for H5 and Y7 are for those when followed by Pro in sequence. ND = not determined.

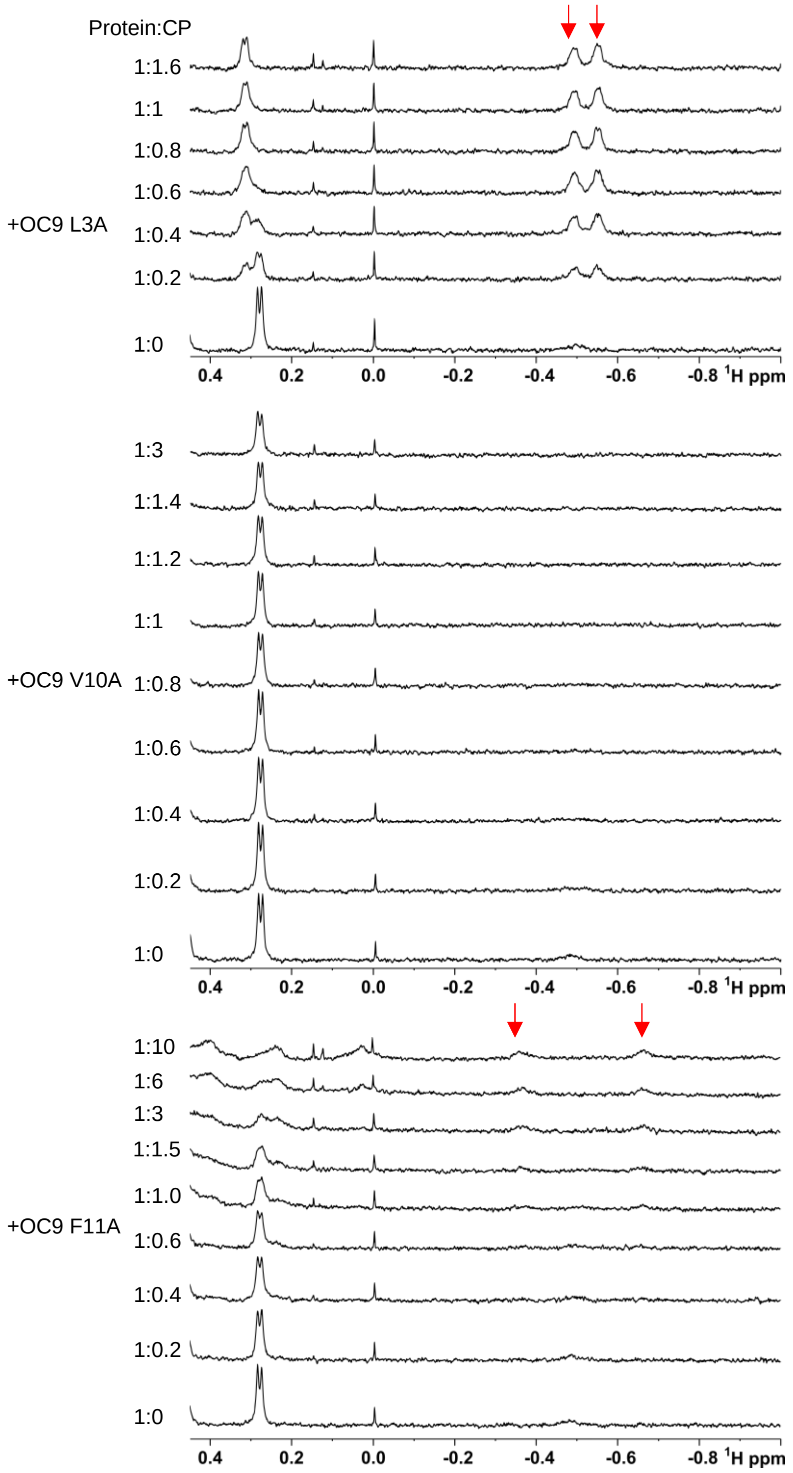

Figure S25. 1D ^{1}H-NMR spectra of OC9 variant titrations against KDM7B PHD-finger

The shift region containing the OC9 V10 γ-methyl groups centred around -0.5 ppm (marked in red) is shown. No signals in this region were observed for OC9 V10A. The titrations of OC9 V10A/F11A showed progressive peak broadening and weakening of multiple residues within ^{1}H-^{15}N HSQC spectra up to 1:1 protein:peptide ratios, indicating an intermediate exchange regime consistent with their lower affinities, with the least affected protein signals corresponding to residues primarily located in the α1 and α3 regions.

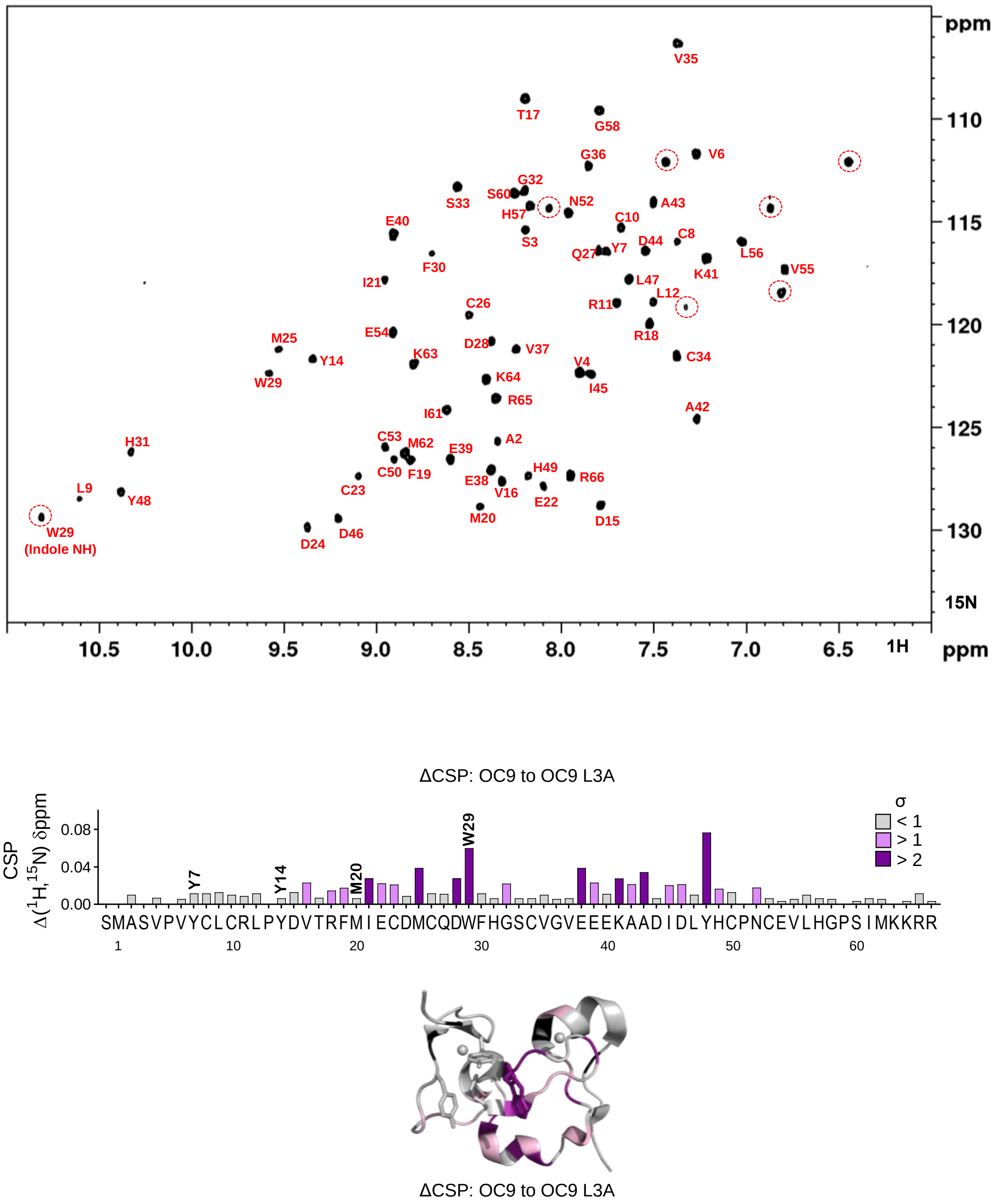

Figure S26. ^{1}H-^{15}N HSQC spectrum of the KDM7B PHD-finger in complex with OC9 L3A.

Annotated with the assigned backbone amide NH residues and the W29 indole, by nearest neighbour assignment to the OC9 complex. Red circles indicate side-chain associated NH. The change in CSP relative to the OC9 complex is plotted and coloured by standard deviation, mapped onto PDB: 3KV4, indicating the OC9 L3 residue may bind adjacent to the cage within a pocket encompassed by α1, α2, β4 and β5. OC9 L3A:PHD is 1.6:1.

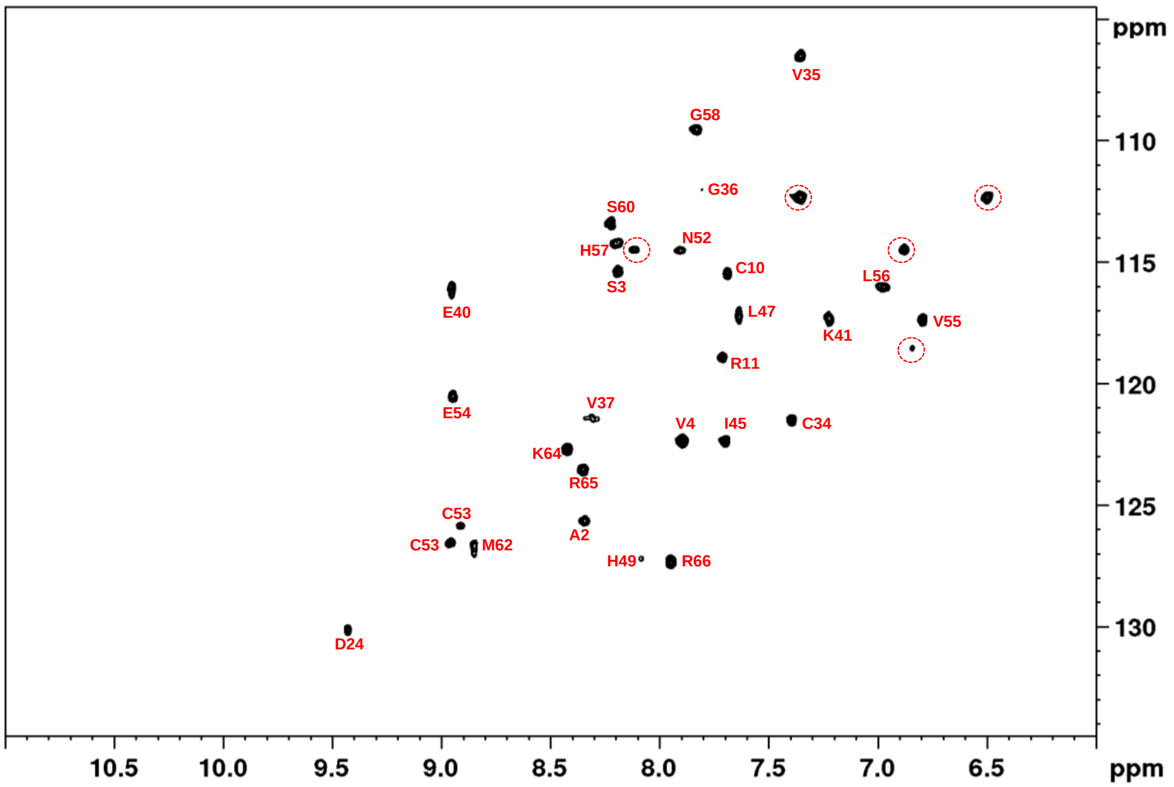

Figure S27. ^{1}H-^{15}N HSQC spectrum of the KDM7B PHD-finger in complex with OC9 V10A.

Annotated with the assigned backbone amide NH residues, by nearest neighbour assignment to the OC9 complex and apo protein. Red circles indicate side-chain associated NH. OC9 V10A:PHD is 3:1.

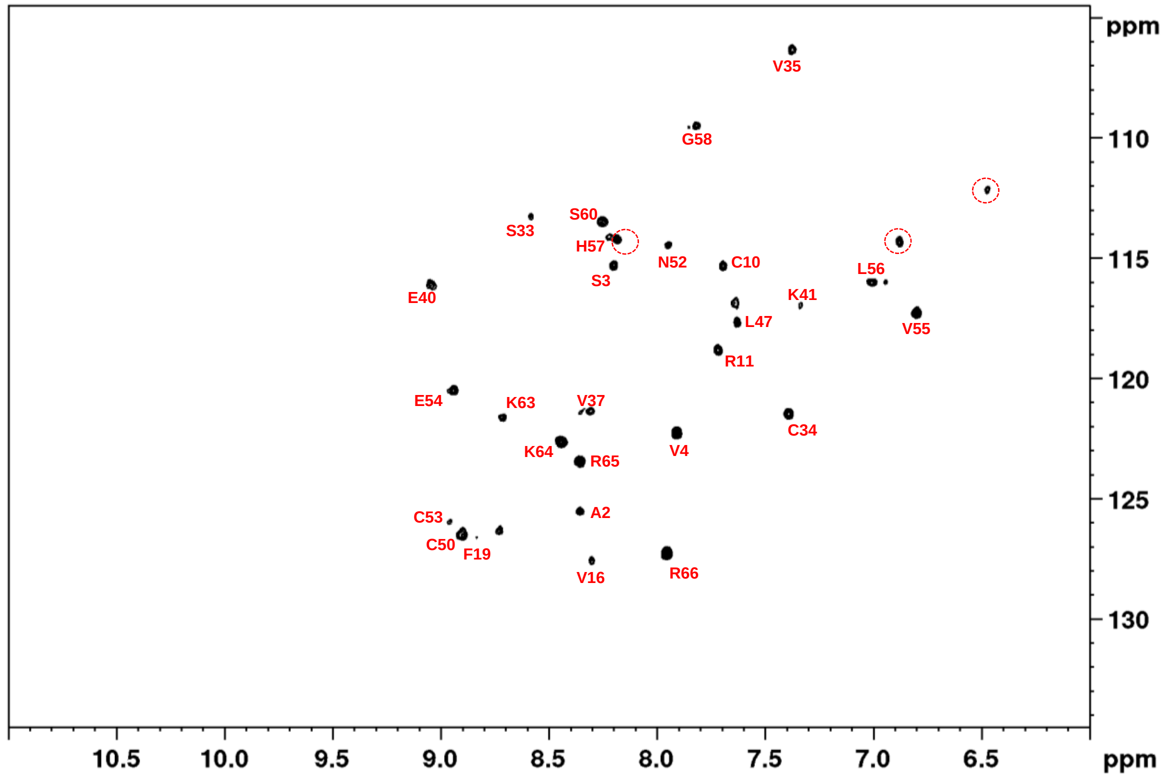

Figure S28. ^{1}**H-**^{15}**N HSQC spectrum of the KDM7B PHD-finger in complex with OC9 F11A.**

Annotated with the assigned backbone amide NH residues, by nearest neighbour assignment to the OC9 complex and apo protein. Red circles indicate side-chain associated NH. OC9 F11A:PHD is 10:1. Unclear nearest neighbour signals are left unassigned.

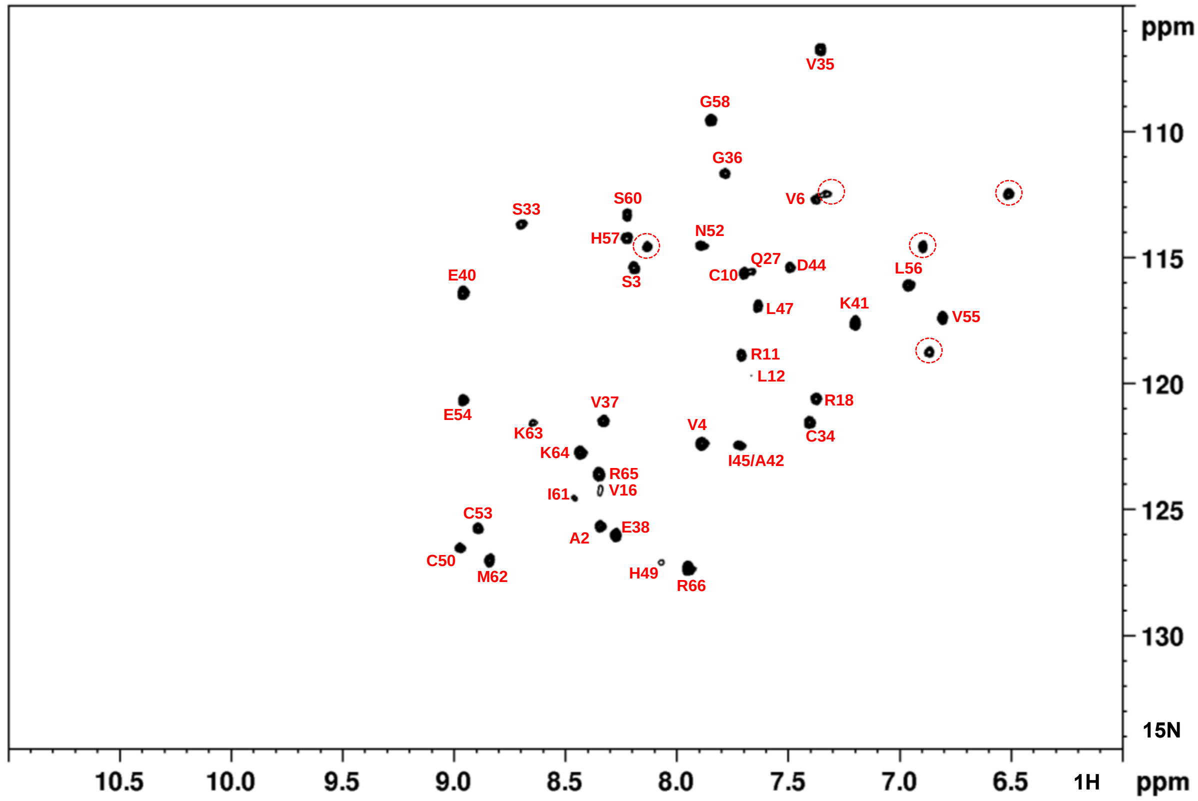

Figure S29. **^{1}H-^{15}N HSQC spectrum of the KDM7B PHD-finger in complex with OC9 V10DV.**

Annotated with the assigned backbone amide NH residues, by nearest neighbour assignment to the OC9 complex and apo protein. Red circles indicate side-chain associated NH. OC9 V10DV:PHD is 5:1.

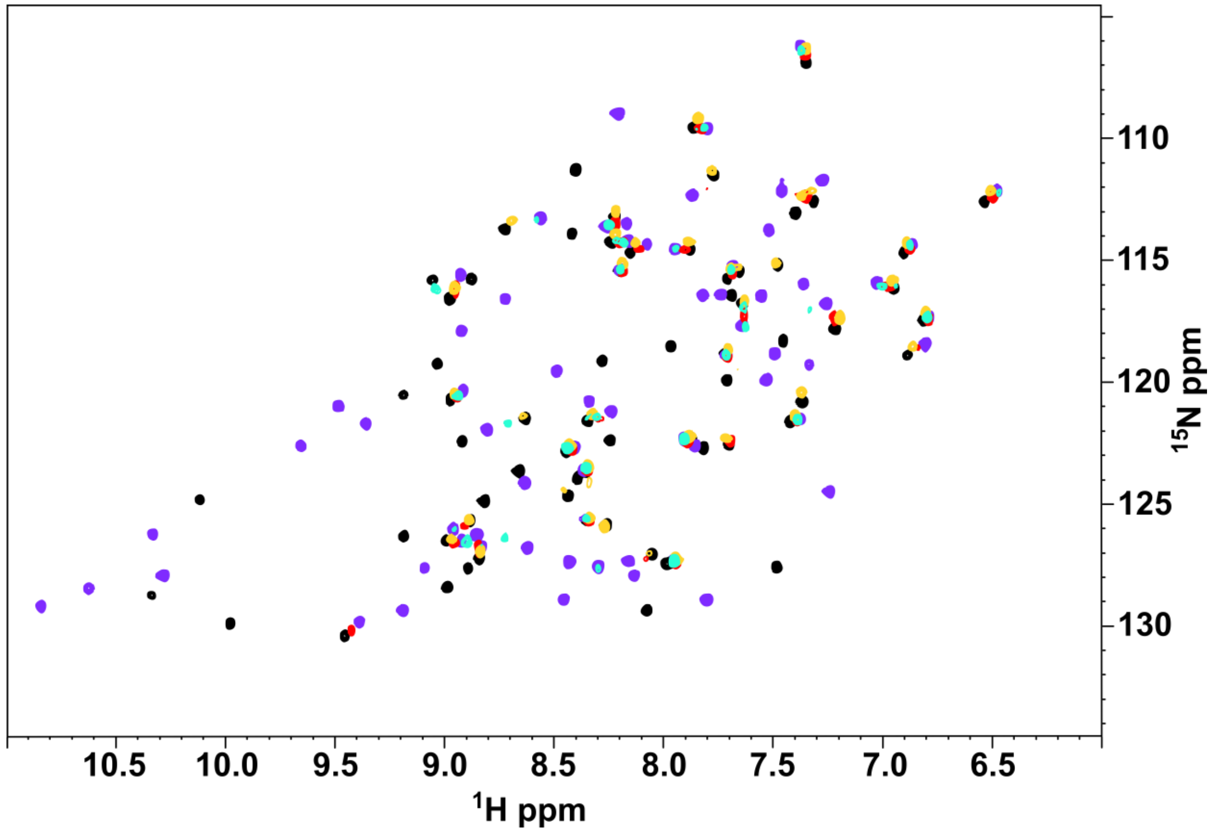

Figure S30. ^{1}H-^{15}N HSQC spectra overlay of the KDM7B PHD-finger complexed to different OC9 variants.

Apo KDM7B PHD-finger (black) is compared to complexes with complexed peptides: OC9 (purple 1:1); OC9 V10A (red 3:1); OC9 V10DV (yellow 5:1); OC9 F11A (cyan 10:1).

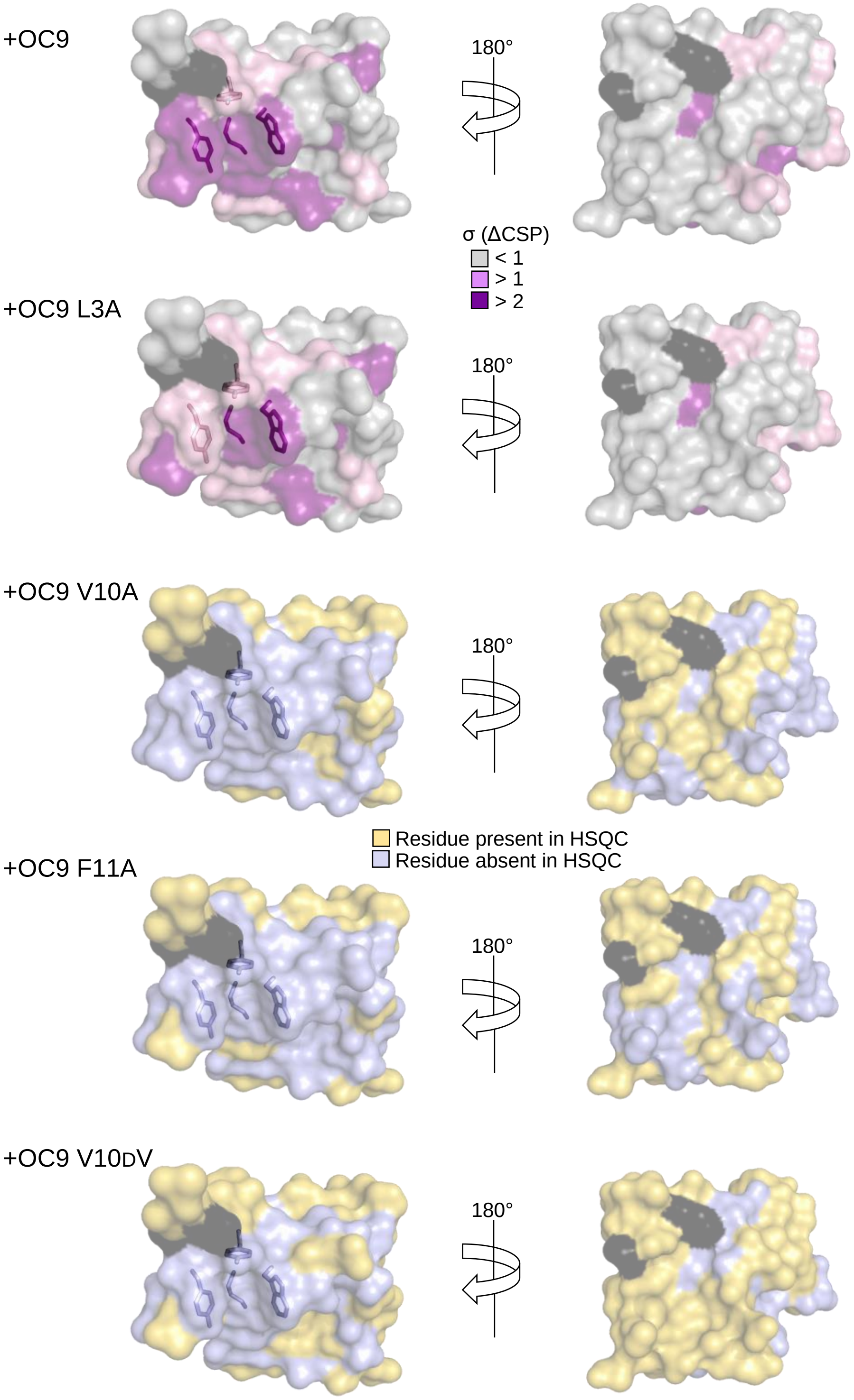

Figure S31. Surface mapping of NMR CSP.

Affected residues (complex versus apo protein) for OC9 and OC9 L3A complexes with the KDM7B PHD-finger, are mapped to PDB: 3KV4. For OC9 V10A, OC9 F11A and OC9 V10DV complexes, the presence (yellow) or absence (blue) of residues within their ^{1}H-^{15}N HSQC spectra is shown. Absent residues are likely to be involved in fast-exchange low-affinity binding, supporting the cage-containing PHD-finger face being the primary OC9-interaction site. Cage residue side chains are shown.

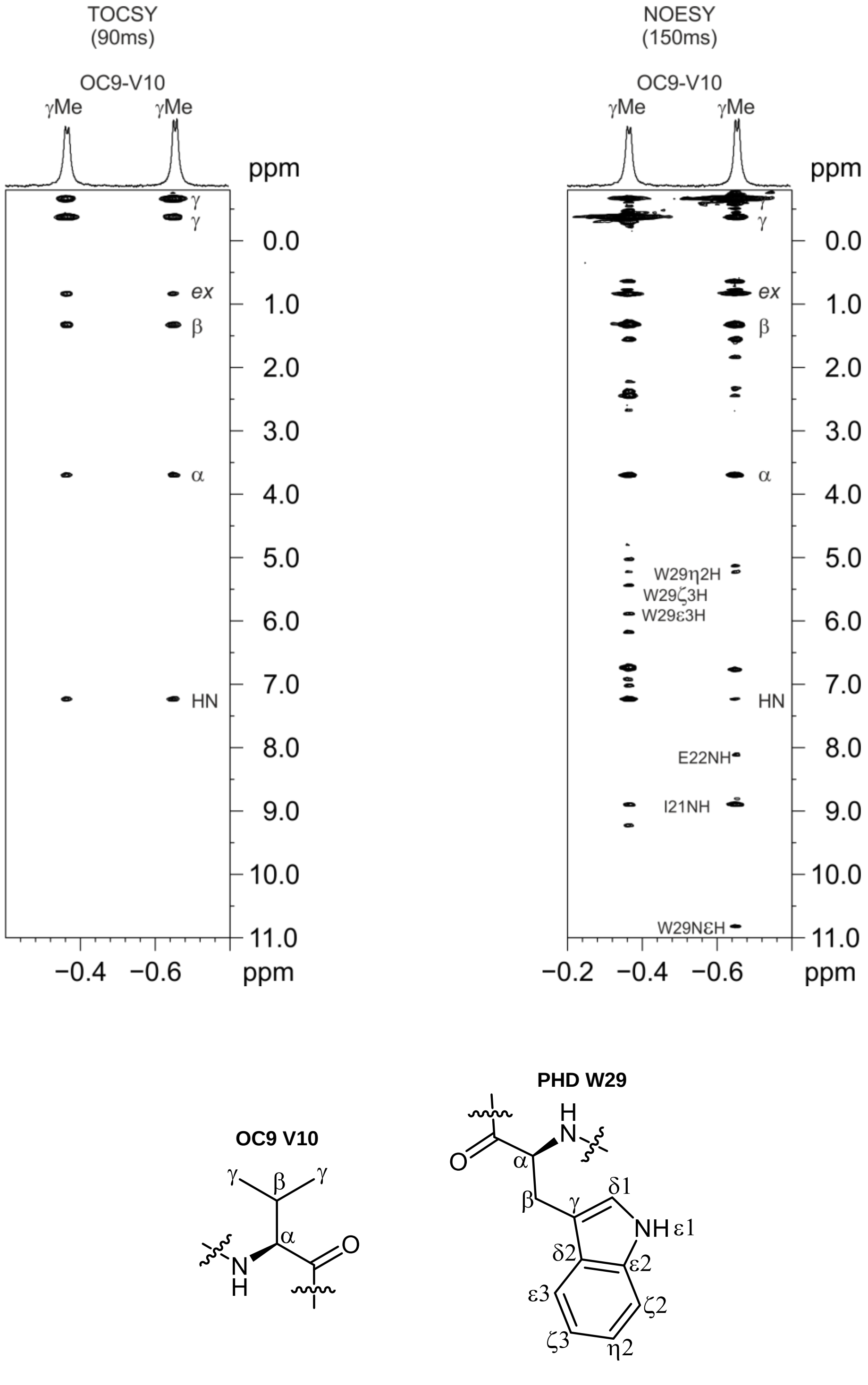

Figure S32. NMR identification of OC9 V10 methyl signals.

NMR correlations identifying the OC9 V10 γ-methyl groups to be shielded and centred around -0.5 ppm (TOCSY) with OC9 in complex with KDM7B PHD-finger protein, with NOE contacts to least the I21, E22 and W29 protein residues (NOESY). *ex* = signal from free exchange of OC9 peptide by ROESY.

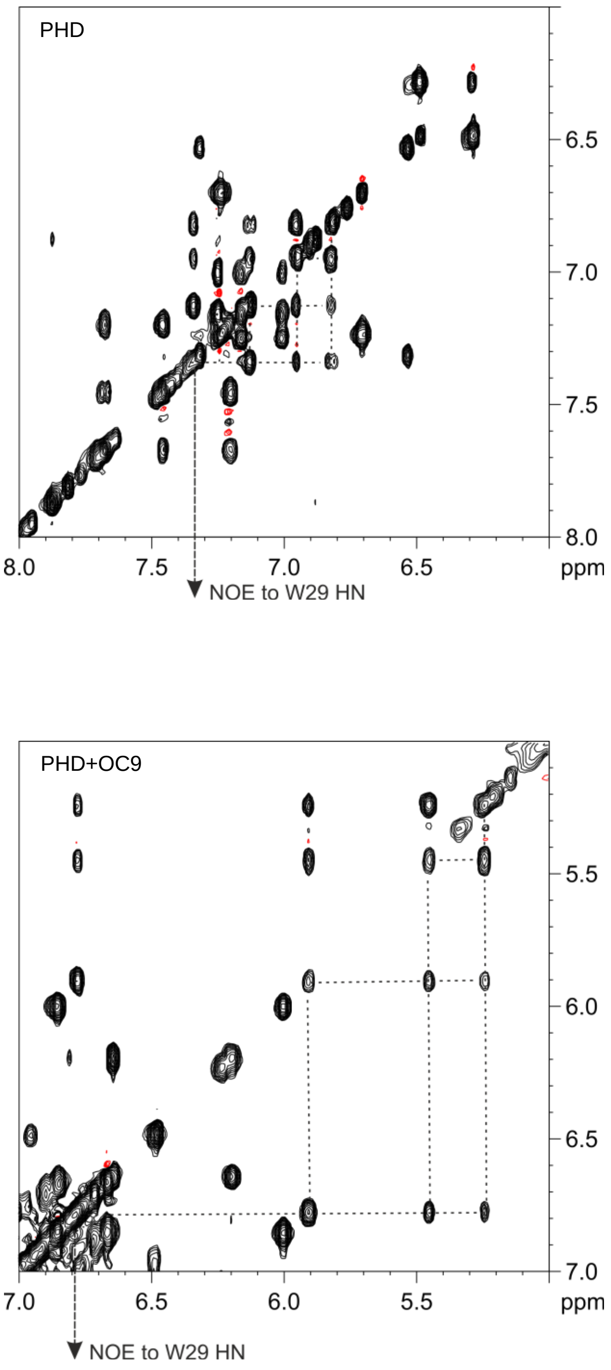

Figure S33. 2D TOCSY protein NMR traces for cage residue W29.

Highlighting correlations within the KDM7B PHD-finger W29 residue, for the apo and OC9-bound protein. OC9 causes shielding of the W29 aromatic side chain chemical shifts.

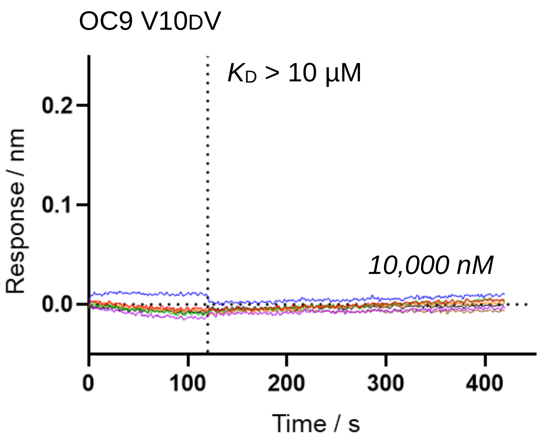

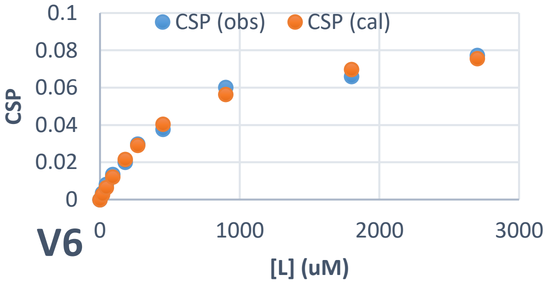

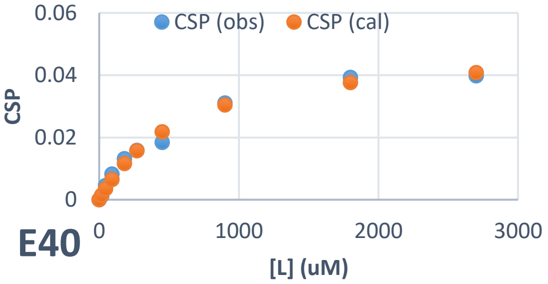

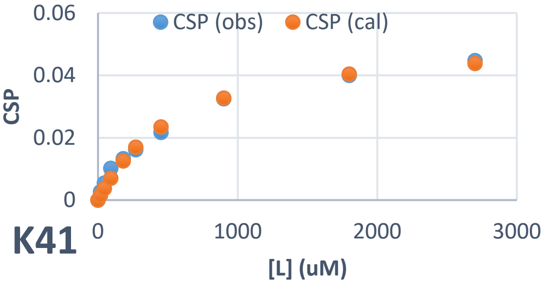

V6 K_D = 504 µM

E40 K_D = 504 µM

K41 K_D = 502 µM

Avg K_D = 503 µM

St. Dev. = 1.3

Figure S34. OC9 V10DV affinity assessment.

Top: BLI showed no discernible affinity for KDM7B PHD-finger at up to 10 µM OC9 V10DV peptide. **Bottom**: Three ^{1}H-^{15}N NMR HSQC peaks showing the clearest titration profiles for OC9 V10DV complex with KDM7B PHD-finger were fitted and their calculated K_D values averaged. Chemical shift perturbation (CSP) values ($\Delta\delta_{obs}$) were plotted against total ligand concentrations $[L]_t$ and data fitted to the binding isotherm for 1:1 complex (n=1) using the solver tool in Excel, with K_D and $\Delta\delta_{max}$ treated as variables, whereby the differences between calculated (cal) and experimental (obs) CSPs were subject to least squares fitting:

$$\Delta\delta_{obs} = \Delta\delta_{max}\left\{(n[P]_t + [L]_t + Kd) - [(n[P]_t + [L]_t + K_d)^2 - 4n[P]_t[L]_t]^{1/2}\right\} \Big/ 2n[P]_t$$

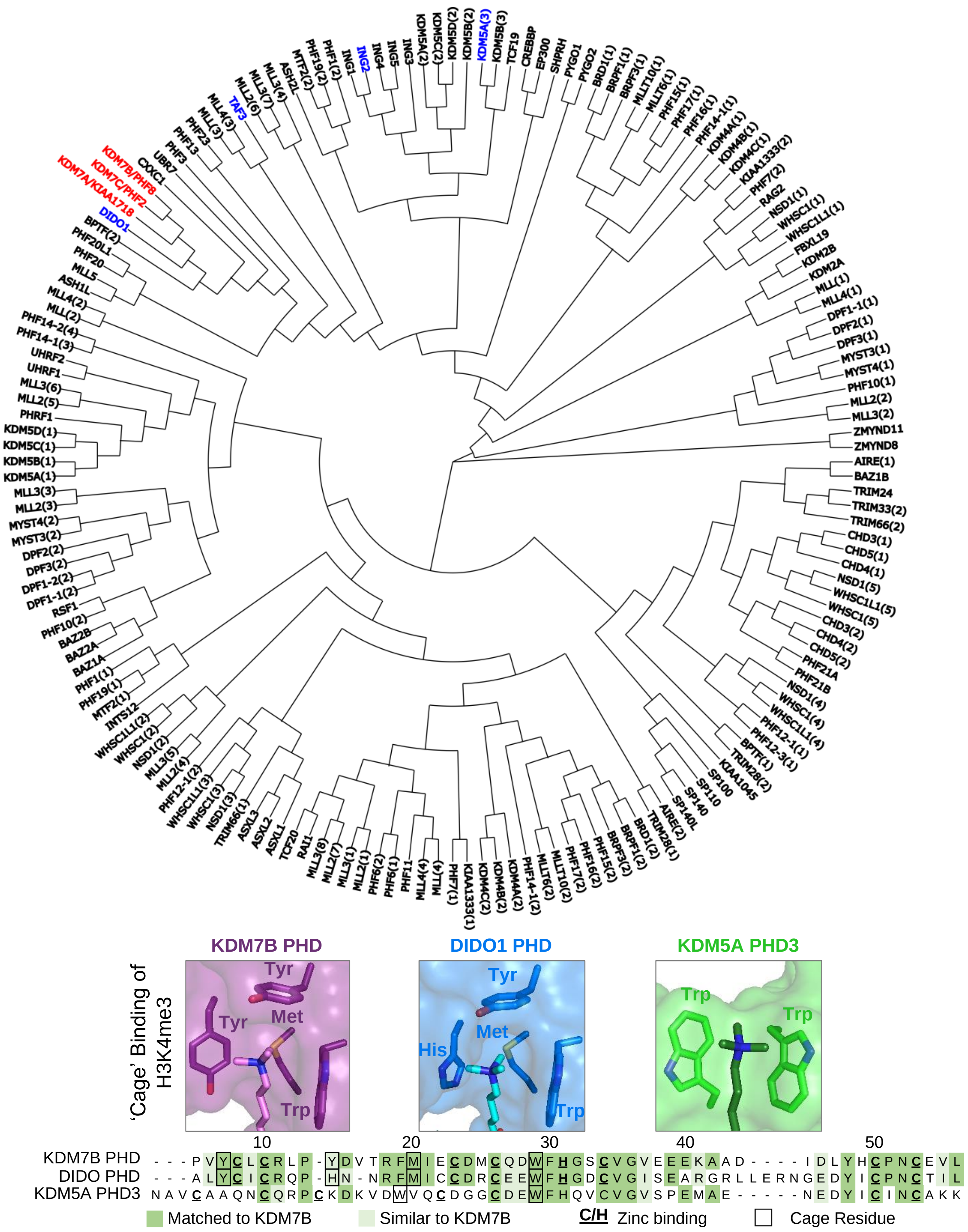

Figure S35. PHD-finger phylogenetic tree and selected aromatic cage comparison.

(Top) PHD-finger domain phylogenetic tree adapted from SGC ChromoHub. The KDM7 sub-family is marked in red. Other proteins that OC9 was screened against are marked in blue. (Bottom) Comparison of H3K4me3-binding cages in three PHD-fingers. Sequences were taken from Uniprot and aligned using Clustal Omega, with amino acid similarity relative to KDM7B from BLASTP. Cage and zinc binding residues determined by inspection of crystal structures using PyMOL. KDM7B: 3KV4, DIDO: 4L7X, KDM5A PHD3: 3GL6.

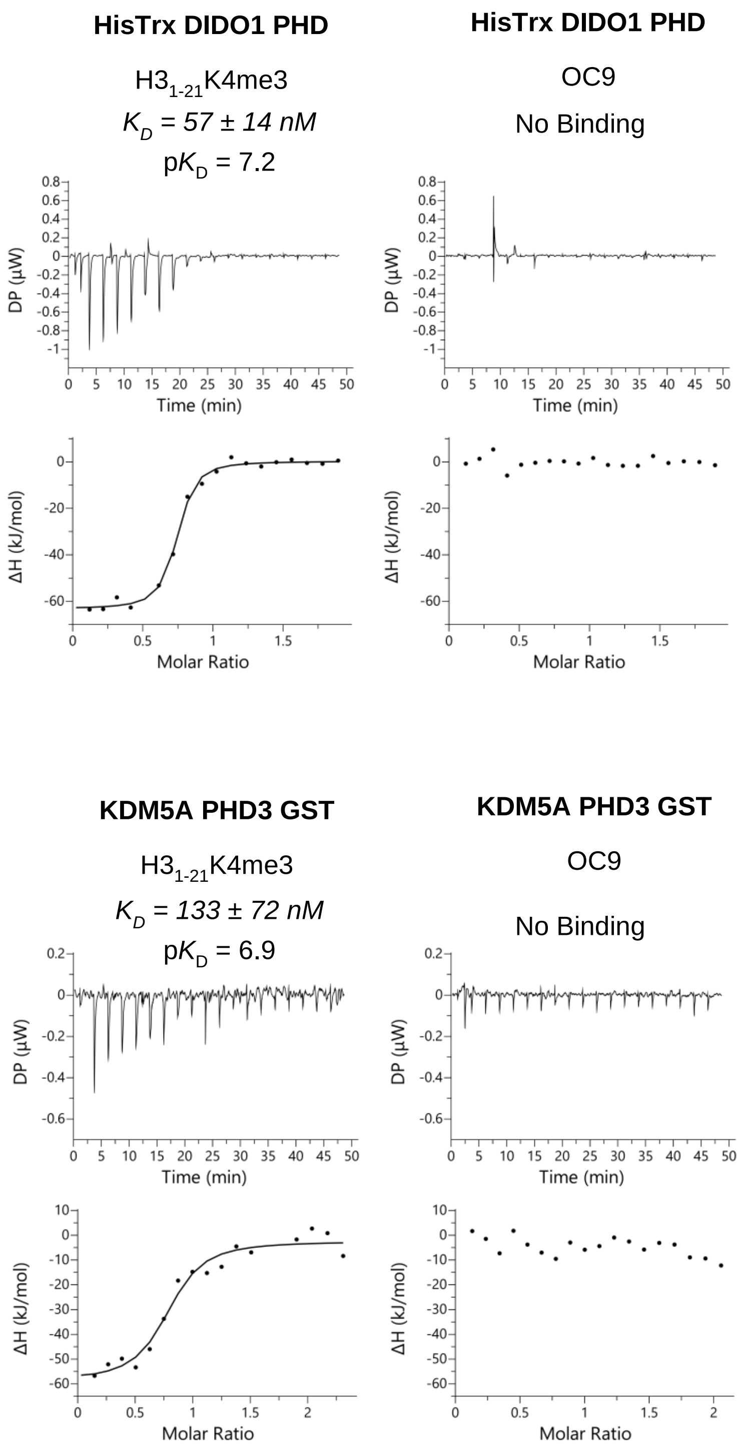

Figure S36. ITC curves for DIDO1 and KDM5A vs OC9 and H3K4me3.

Top panels are raw heat of injection, bottom panels are normalised data fitted to 1:1 binding model. OC9 and $H3_{1\text{-}21}K4me3$ with DIDO1 PHD-finger and KDM5A PHD3-finger proteins. HisTrx = His+thioredoxin tag, GST = glutathione S-transferase tag. For results of fit and conditions see Figure S10.

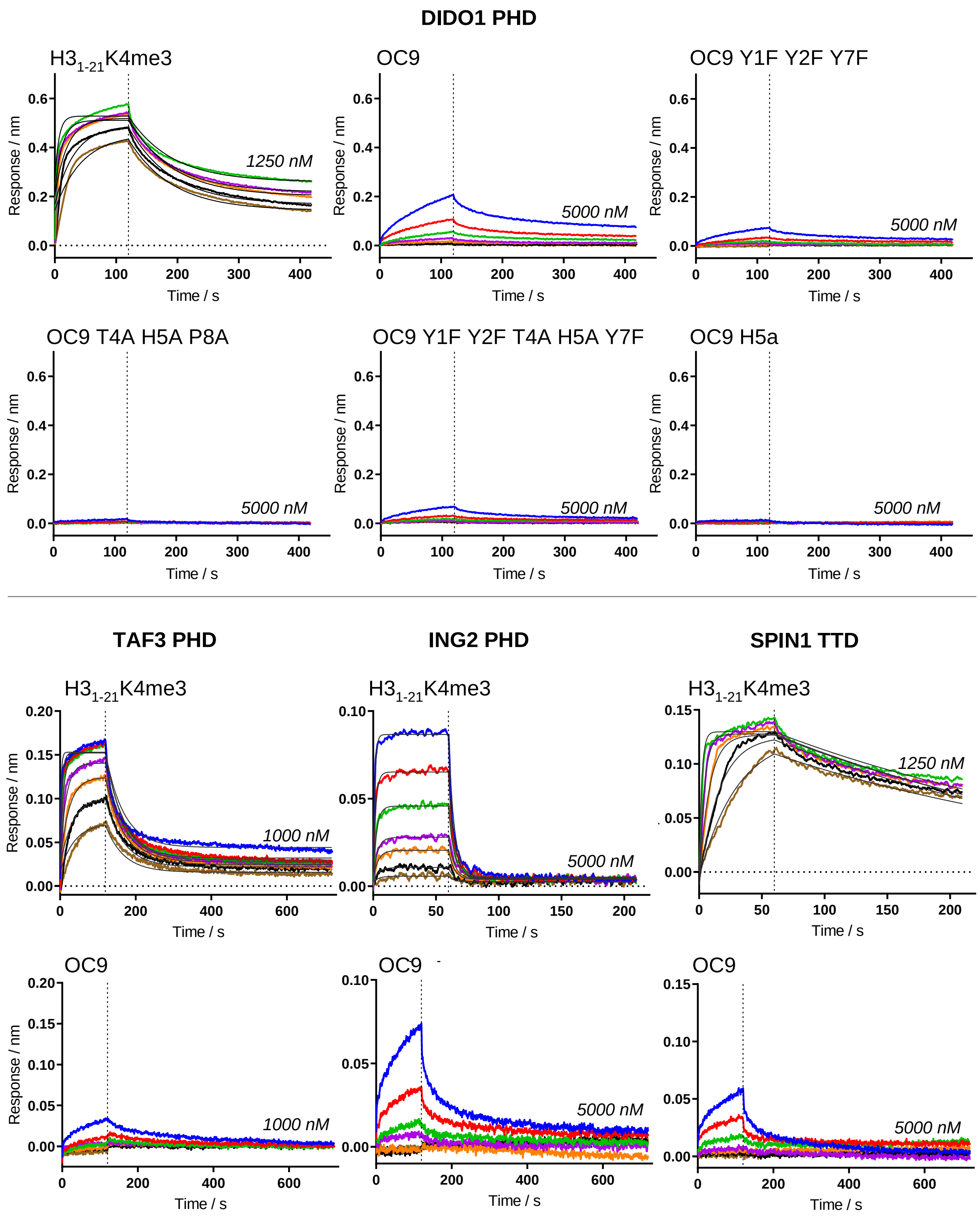

Figure S37. BLI traces of H3$_{1-21}$K4me3 and OC9 peptides with H3K4me3-reader domains.

Top: screening against the DIDO1 PHD-finger. Bottom: screening against other reader domains. Response trace for each peptide concentration is represented by a different colour, with the fitted curves where they could be fitted overlaid in black. See Figure S5 and S19 for results of fit and the top concentration plotted, with 2-fold dilutions thereafter.

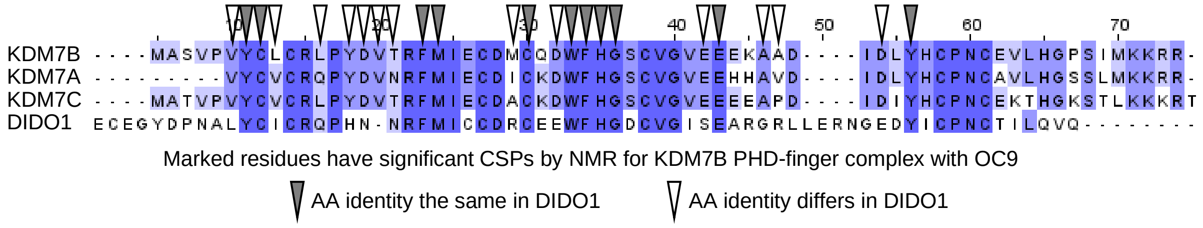

Figure S38. Aligned KDM7A/B/C and DIDO1 PHD-finger sequences.

KDM7B residues that experience significant CSP upon OC9-binding by NMR are marked (black triangle), and amongst these the amino acids which differ in identity in DIDO1 are marked (red line). Alignment performed using Clustal Omega, with amino acid colouring in JalView (dark blue is present in all 4 sequences, light blue is present in 2 or 3, white is present in 1 only).

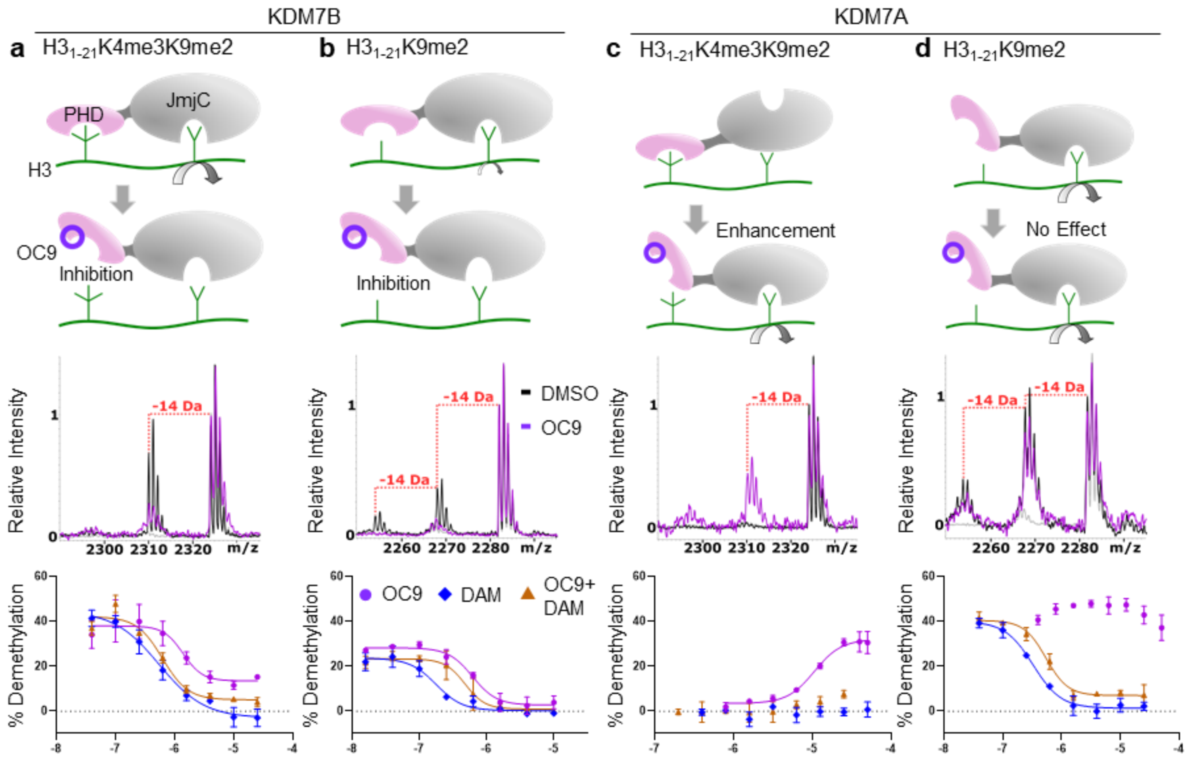

	KDM7B		KDM7A	
H3$_{1-21}$	K9me2	K4me3K9me2	K9me2	K4me3K9me2
(µM)	IC_{50}	IC_{50}	IC_{50}	EC_{50}
**OC9**	0.51 ± 0.15	1.19 ± 0.21	N.E.	11.3 ± 0.9
**DAM**	0.24 ± 0.08	0.46 ± 0.24	0.24 ± 0.12	No Effect
**OC9+DAM (1:1)**	0.44 ± 0.05	0.54 ± 0.23	0.65 ± 0.15	No Effect

Figure S39. KDM7B and KDM7A dual domain demethylation assays by MALDI-TOF-MS.

Schematic representation of KDM7B and KDM7A PHD-finger cross-talk with the JmjC domain to regulate demethylase activities against histone H3 peptides. The effect of OC9 is depicted, and example MALDI-TOF-MS spectra are shown comparing OC9 (highest concentration) to DMSO. Representative IC_{50} plots are shown and IC_{50} values (average of two experiments in triplicate) tabulated.

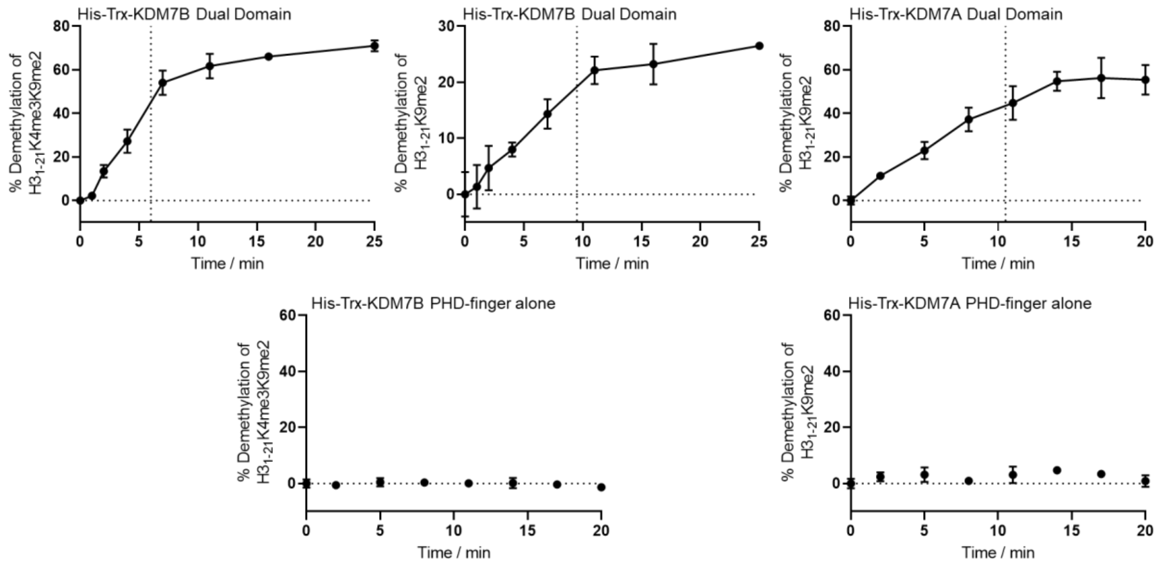

Figure S40. KDM7B and KDM7A demethylation activity time-course assays by MALDI-TOF-MS.

Time-course incubations were conducted to find appropriate conditions for compound testing. Dashed vertical lines indicate timepoint chosen for ‘stop’ point of compound testing assays. His-Trx tagged KDM7B dual domain was used at 400 nM, 25°C for $H3_{1\text{-}21}$K4me3K9me2 and 30°C for $H3_{1\text{-}21}$K9me2. His-Trx tagged KDM7A dual domain was used at 550 nM and 30°C for $H3_{1\text{-}21}$K9me2. His-Trx tagged KDM7B PHD-finger and KDM7A PHD-finger were used at 1 µM and 30°C. All assays used 100 µM Fe(II), 100 µM 2OG, 200 µM NaAsc, with 2.5 µM histone peptide. See supplementary methods for full conditions.

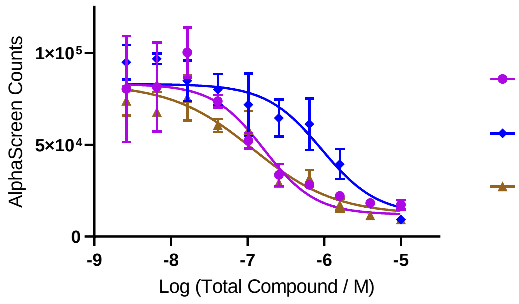

	IC_{50} (nM)	pIC_{50}
OC9	163	6.8
DAM	925	6.0
OC9+DAM (1:1)	119	6.9

Figure S41. KDM7B dual domain demethylase AlphaScreen assay.

An AlphaScreen IgG detection kit with anti-H3K9me1 antibody was used to screen the effect of compounds against turnover of $H3_{1\text{-}21}$K4me3K9me2-GGK-biotin substrate (150 nM) after 10 minutes with KDM7B dual domain (20 nM) and co-factors: 20 µM 2OG, 10 µM Fe^{II}, 100 µM Asc. DAM = daminozide.

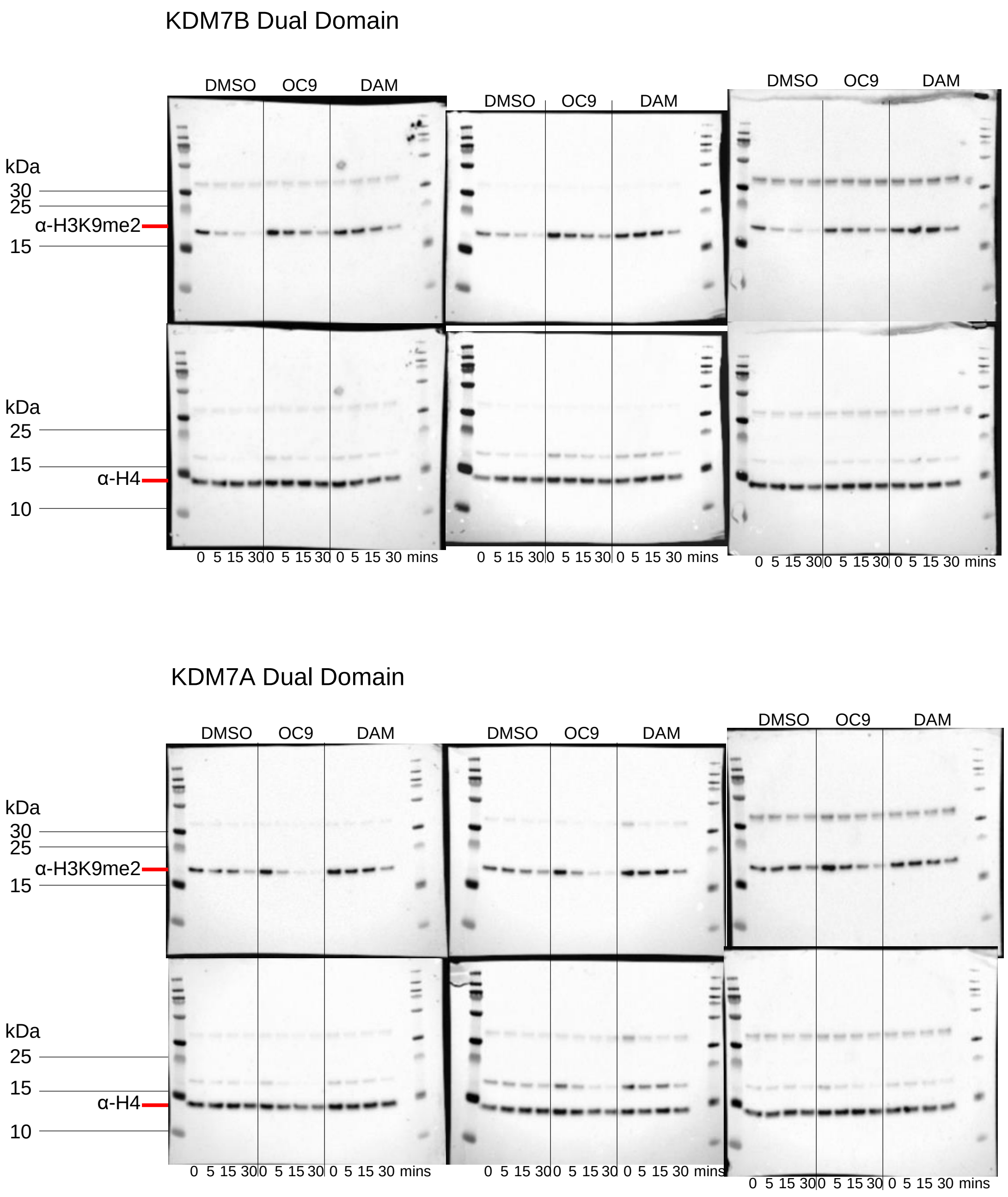

Figure S42. Calf histone demethylation assay Western Blots.

Corresponds to the plotted and fitted time-course in Figure 5. Each blot is an independent assay experiment. DAM = daminozide.

a.

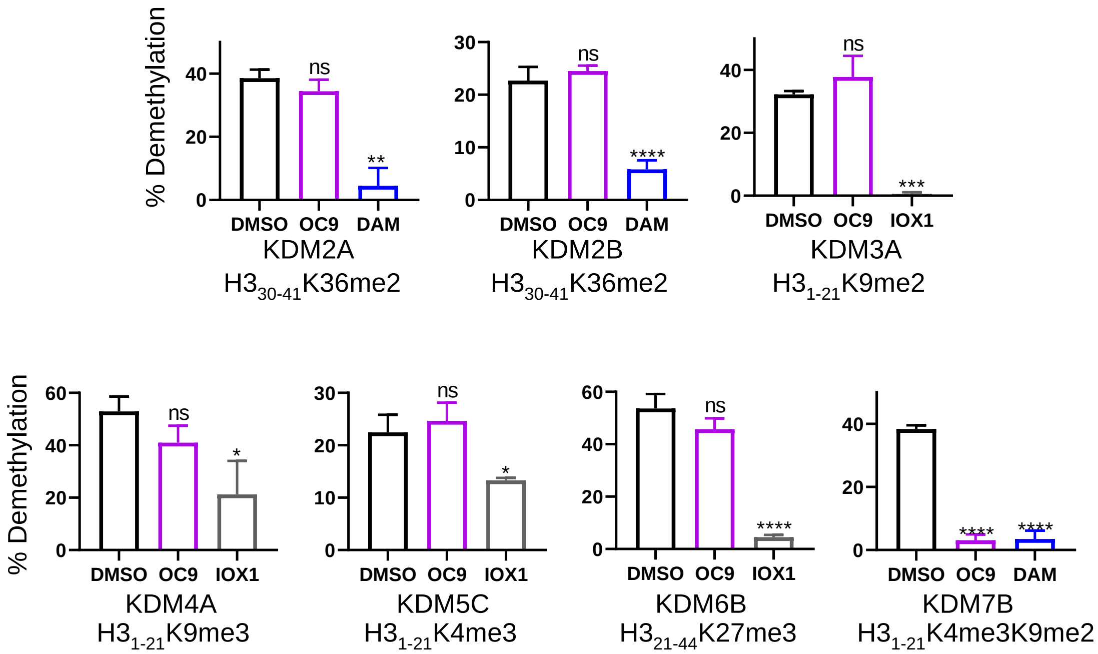

b.

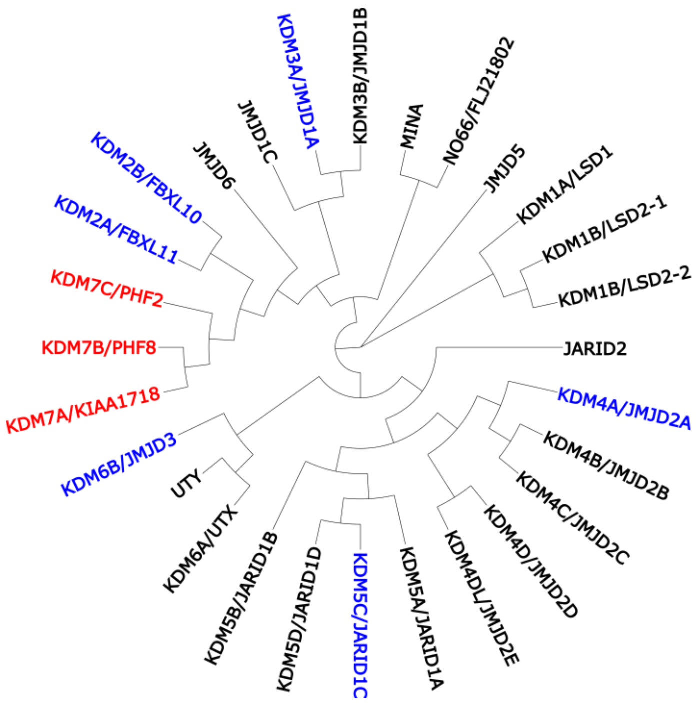

Figure S43. KDM Cross Screening by MALDI-TOF-MS

(a) Screening of OC9 and a small molecule JmjC-inhibitor (DAM = Daminozide, or IOX1) by MALDI-TOF-MS demethylation assays, using 20 µM compounds or DMSO (0.4 %). KDM2A (0.3 µM) and KDM2B (0.2 µM) were full-length proteins. KDM3-6 (0.1 to 1.0 µM) and KDM7B (2.0 µM) were JmjC-containing constructs. Histone peptide substrates (noted under each enzyme) were at 10 µM for KDM2-6 and 5 µM for KDM7B. See supplementary methods for experimental details. One-way ANOVA multiplicity adjusted p value, comparison to DMSO control: ns >0.12, *<0.0332, **<0.0021, ***<0.0002, ****<0.0001. Each protein screened in technical duplicate or triplicate. (b) The phylogenetic tree for the catalytic domains of KDM proteins was adapted from the SGC ChromoHub, with the KDM7 sub-family marked in red. Other sub-families that OC9 was screened against are marked in blue.

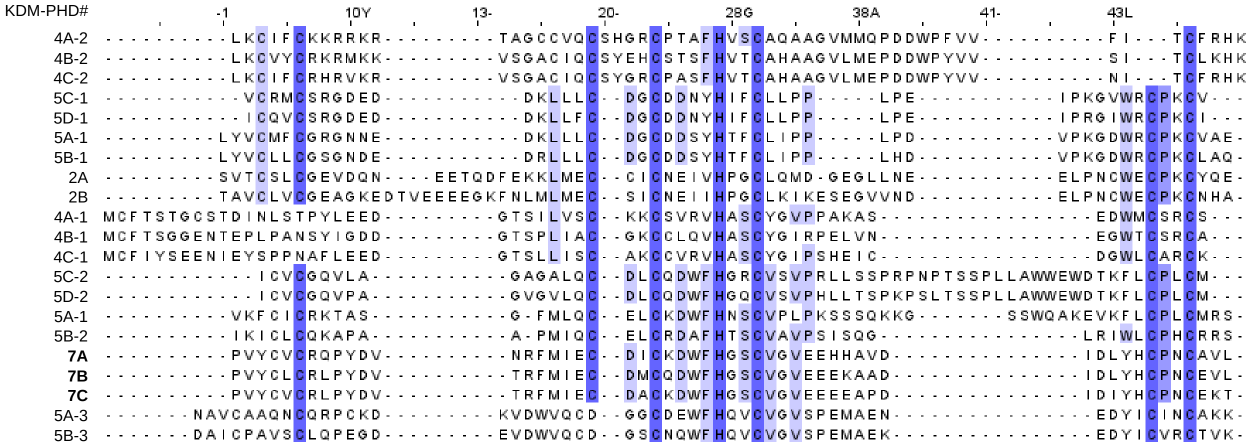

Figure S44. KDM PHD-finger sequence alignments.

Canonical KDM PHD finger sequences from Uniprot were multiply aligned using Clustal Omega. Highly conserved residues are marked in blue using JalView. Little similarity is seen between sub-families. Highly conserved Cys and His residues are zinc binding.

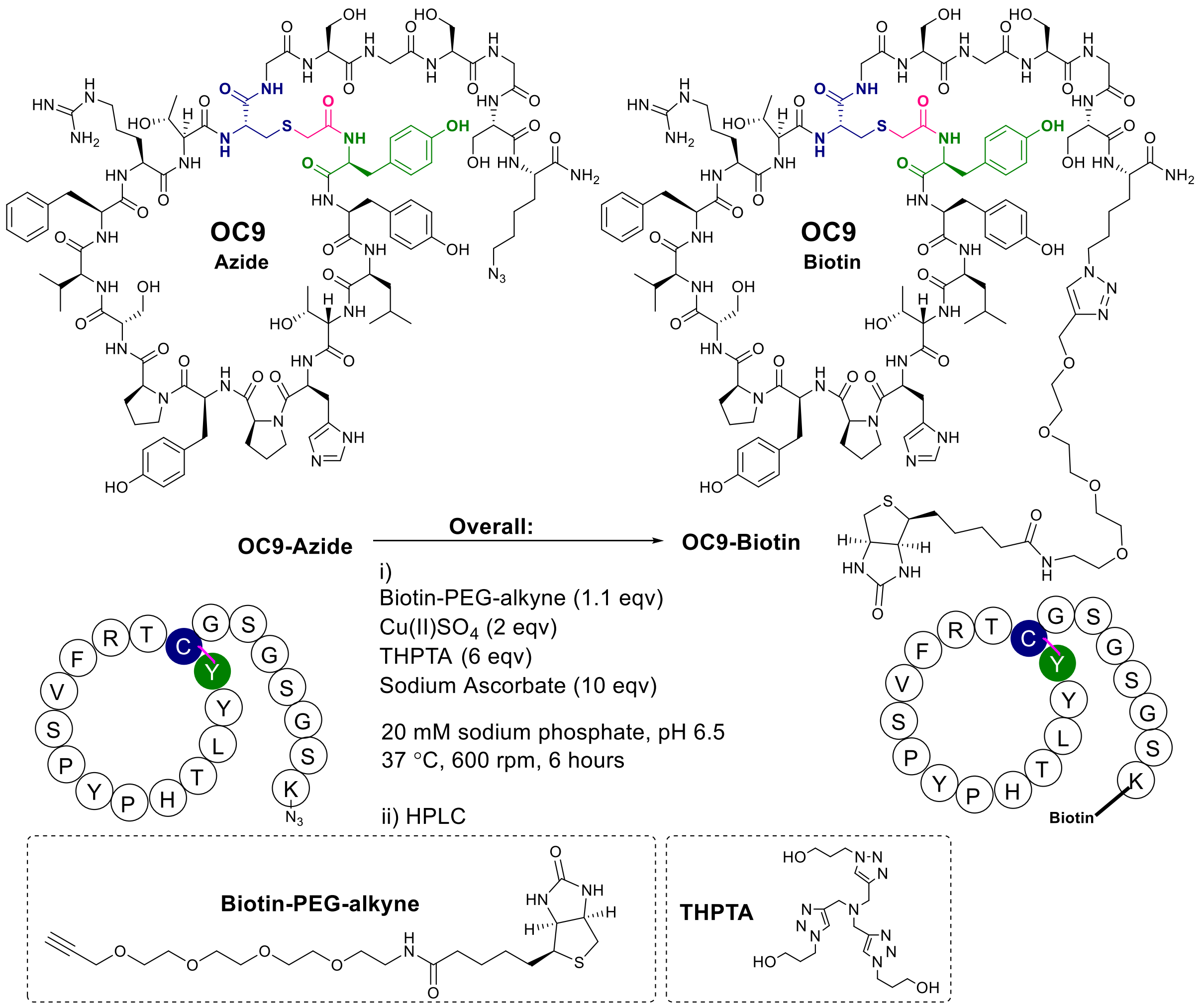

Figure S45. OC9-Biotin synthesis scheme.

Overview of the synthesis of OC9-Biotin (OC9-Bt). OC9-Azide was made using standard Fmoc-SPPS with Fmoc-Lys(N_3). OC9-Azide (sequence: YYLTHPYPSVFRTCGSGSGSK(N_3)) was synthesised by SPPS. Full method in Supplementary Methods.

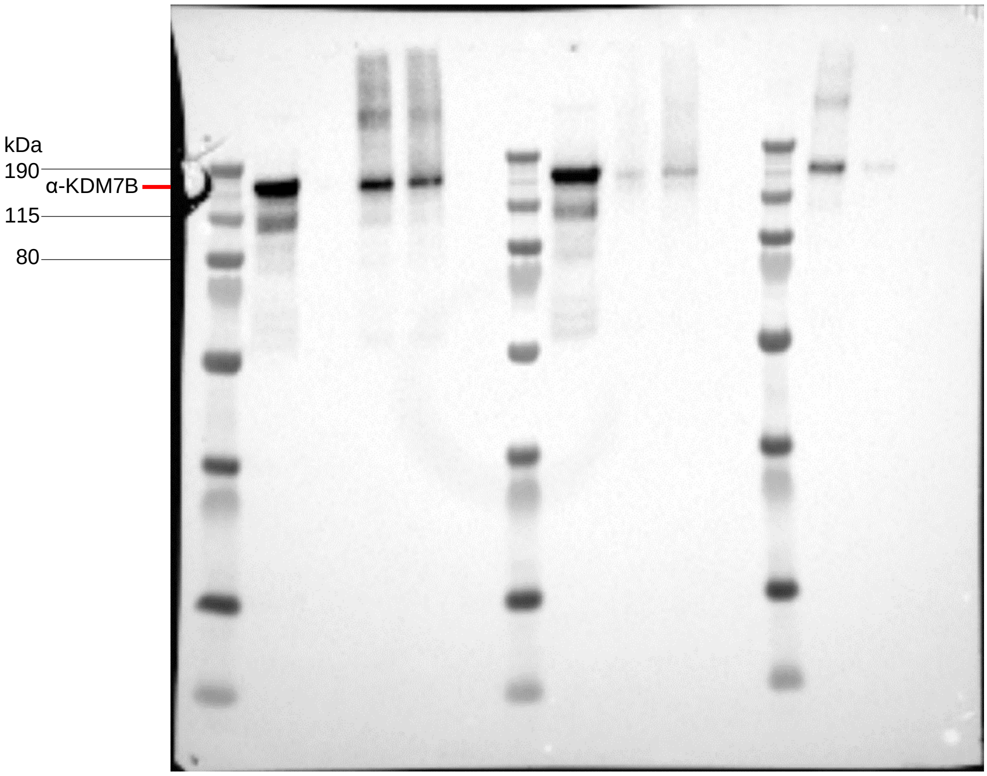

Figure S46. Pull-downs from HEK293-T nuclear lysates.

Full Western blot corresponding to Figure 6a.

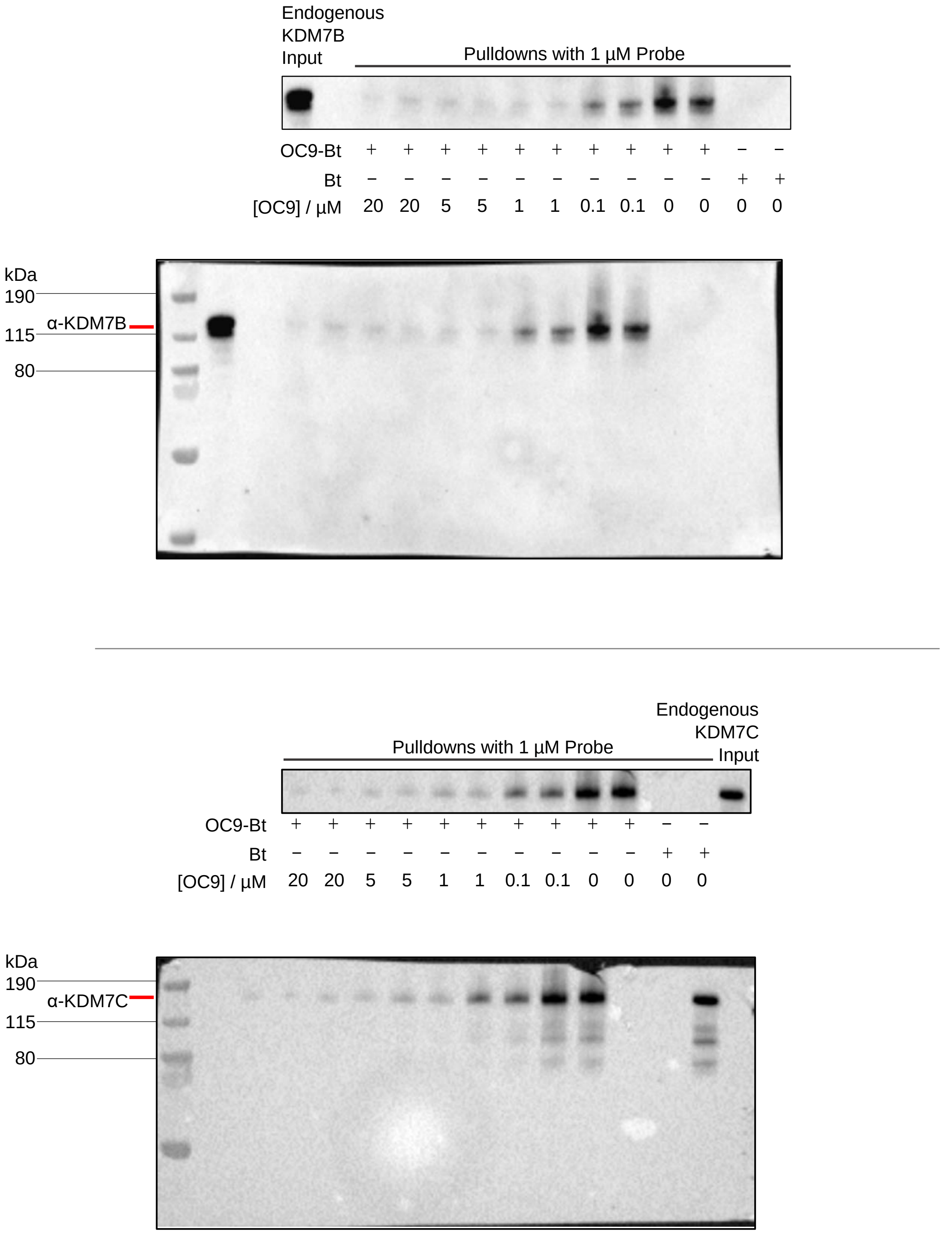

Figure S47. Western blots for chemoproteomics pull-downs.

Confirmation of the dose dependent competition of OC9 with OC9-Bt recovery of endogenous KDM7 through pull-down from SUP-T1 nuclear lysates. The same samples were used for proteomic analysis. The full blot images are given underneath.

Supplementary Methods

mRNA Display Selection and Next-Generation Sequencing Analysis

Library preparation: DNA templates for transcription were generated by PCR as described[2] with minor modifications. mRNA was transcribed from DNA using T7 RNA polymerase, purified by phenol/chloroform extraction followed by ethanol precipitation. In the first selection round a 1:1:1 molar ratio of NNK10/NNK11/NNK12 (see below for example library sequence) was used. An exemplar round of selection is described: The mRNA library (10 µM) was ligated[3] in T4 RNA ligase buffer, with puromycin-linker (15 µM), RNase inhibitor (0.2 U), T4 RNA ligase (18.8 U) and MilliQ H_2O was added to a final volume of 7.5 µL. The reaction was assembled in a PCR tube at 20 °C for 1 hour, then 70 °C for 10 minutes. All translation components were kept on ice during preparation and assembled in a low-binding tube. The puromycin-oligo ligated RNA encoding for the peptide library (2.5 µM) was translated in vitro using NEB PURE express kit (ΔRF1, ΔMet) supplemented with 500 pmol of *N*-chloroacetyl-L-tyrosine-$tRNA^{fMet}_{CAU}$ [4] in 5.9 µL for 1 hr at 37 °C, then 10 minutes at 60 °C. Reverse transcription (10 µL) was carried out at 42 °C for 1 hour using M-MLV RTase (50 U), Primer P2 (1.78 µM), M-MLV buffer (0.53X), dNTPs (0.27 mM) and input (translation reaction). On completion, the RT mixture was diluted with 10 µL of 2X selection buffer (selection buffer: PBS, 0.01% BSA, 0.05% Tween20, 2 mM beta-mercaptoethanol (BME)). 0.25 µL from the diluted RT mixture was taken and further diluted to 1000 µL in H_2O (Sample 1). All subsequent manipulations were carried out at 4 °C using pre-chilled buffers.

Binding cyclic peptide selection: To remove non-specific binders, the mRNA/cDNA-cyclic peptide library was initially applied to streptavidin coated magnetic beads (80 µg, pre-washed with selection buffer and half of beads loaded with Biotin) and incubated gently with agitation (30 min, 4 °C). The beads were pelleted using a magnet, and the supernatant was transferred to a tube with fresh beads and the process was conducted three times in total. 0.25 µL from the screened library mixture was taken and further diluted to 1000 µL in H_2O (Sample 2). Target protein was loaded to beads by taking 10 µL of $KDM7B_{PHD+JmjC}$-biotin (36 µM) (thawed on ice) and diluting it with an equal volume of selection buffer (with added 10 µM $ZnCl_2$), then 5 µL of the diluted solution was further diluted with selection buffer (no added $ZnCl_2$) to a final protein concentration of 1 µM. 100 µg of pre-washed streptavidin beads were re-suspended in 20 µL of 1 µM $KDM7B_{PHD+JmjC}$-biotin at 4 °C for 30 min with rotation, then beads were pelleted and washed five times with 120 µL of selection buffer. Bead loading was previously determined to be ~2 pmol protein per µL bead slurry. The protein loaded beads were re-suspended in the ~20 µL of library solution and incubated at 4 °C with rotation for 30 min, then pelleted and supernatant removed before re-suspension of the beads in 100 µL of selection buffer and transfer to a fresh low-binding tube. The re-suspension and transfer processes was conducted three times in total. The beads were finally re-suspended in 100 µL of PCR mix (NH4 buffer, 2.5 mM $MgCl_2$, 0.25 mM dNTPs, 0.5 µM P1, 0.5 µM P2), then heated at 95 °C for 5 mins and the supernatant containing the liberated DNA immediately recovered to a fresh tube. Recovered DNA was amplified by PCR and purified by phenol/chloroform extraction and ethanol precipitation, and used as the template DNA for the next round of selection. The percentage recovery was quantitated by qPCR (input sample 1&2 vs recovery) for each round of selection. The recovery at the end of round 5 suggested sufficient enrichment and no further rounds of selection were undertaken.

Next Generation Sequencing: DNA pools from selection rounds were sequenced by Illumina sequencing as previously described[5].

Example DNA library NNK sequence: `ATG(NNK)`$_{10}$`TGCGGCAGCGGCAGCGGCAGCTAG`

DNA Primers: P1: `TAATACGACTCACTATAGGGTTAACTTTAAGAAGGAGATATACATATG`
P2: `TTTCCGCCCCCCGTCCTAGCTGCCGCTGCCGCTGCCGCA`

General Procedure for His-tagged Protein Production

Rosetta2 or BL21 (DE3) E. coli cells were transformed with the desired plasmid (see Supplementary Methods – Expressed Protein Sequences). A 100 mL starter culture was inoculated and grown with kanamycin (50 µg/mL) and chloramphenicol (34 µg/mL) for 16 hours. Expression media (2 L, 2TY media) was inoculated with 100 mL of the overnight starter culture and grown at 37 °C with kanamycin at 220 RPM until OD600 ~1.0. Expression was induced with isopropyl β-D-1-thiogalactopyranoside (IPTG) (0.25 mM final). $ZnCl_2$ was added (10 µM final) for PHD finger containing proteins and incubated for 16 hours at 18 °C. Cells were harvested by centrifugation (8,000 g, 10 min) and pellets stored at -80 °C. The subsequent steps were performed at 4 °C unless otherwise stated. The frozen cell pellet (~10 g) was re-suspended and thawed in ~60 mL of binding buffer [25 mM HEPES, 500 mM NaCl, 5 mM imidazole, pH 7.4, 1 mM TCEP, with DNaseI and Protease Inhibitor Cocktail Tablet (Sigma Aldrich)] with vigorous magnetic stirring until homogenous. Cells were lysed by sonication for 5 min, with 10s on 30s off cycle (25 % AMP, Sonics VCX 500W), and centrifuged (10,000 g, 20 min) to removed cell debris. The cell lysate was filtered (0.45 µm) before application to a 1 mL HisTrap FF Crude on an Akta Pure system. Protein was eluted by imidazole gradient with elution buffer [25 mM HEPES, 500 mM NaCl, 250 mM imidazole, pH 7.4, 1 mM TCEP]. Protein containing fractions were combined, concentrated using a molecular weight cut off concentrator (10 kDa MWCO Amicon Ultra, Millipore) and purified by size-exclusion chromatography (SEC) using a gel filtration column (S75, 120 mL) in gel filtration buffer [25 mM HEPES, 200 mM NaCl, pH 7.4, 1 mM TCEP]. For His-thioredoxin tag cleavage, tobacco etch virus (TEV) protease was added in a ~10:1 ratio of Protein:TEV (mg) and incubated for 16 hours, then buffer exchanged into binding buffer using a PD-10 column (GE Healthcare) and re-applied to Ni-affinity resin before SEC. Target protein containing fractions were determined by SDS PAGE gel and concentrated (3 kDa MWCO Amicon Ultra, Millipore), aliquoted and flash frozen in liquid nitrogen for long-term storage at -80 °C. Final protein concentrations were measured by NanoDrop or Pherastar FSX.

Isotopically (^{15}N, ^{13}C) Labelled Protein Production:

Labelled KDM7B PHD (pNH-TrxT vector) protein was produced in minimal growth media with $^{15}NH_4Cl$ and $^{13}C6$-glucose (CK Isotopes). The growth solution (2 L total) was prepared in MilliQ H_2O with Na_2HPO_4 (6 g/L), KH_2PO_4 (3 g/L), $^{15}NH_4Cl$ (2 g/L), NaCl (0.5 g/L), $CaCl_2$ (3 mg/L), then autoclaved and cooled. Immediately before use, filtered (0.22 µm) solutions of 1 M $MgSO_4.7H_2O$ (500 µL/L), 20 % w/v $^{13}C6$-glucose (20 mL/L), 0.5 % w/v vitamin B1 (50 µL/L) were prepared and added to the growth media. A starter culture was grown from transformed Rosetta2 cells in 2TY overnight and used to inoculate the minimal growth media (~10 mL/L) and grown at 37 °C with kanamycin at 220 RPM until OD600 ~0.8. Expression was induced with IPTG (Isopropyl β-D-1-thiogalactopyranoside) (0.25 mM final) and incubated for 16 hours at 18 °C. Harvesting, purification and TEV cleavage procedures then followed the standard unlabelled protocol. Isotopic labelling was confirmed by ESI-MS (See Protein Mass Spectrometry). ^{15}N-only labelled protein was produced similarly, but with unlabelled glucose.

Biotinylated KDM7B Dual Domain Production

KDM7B Dual Domain (1-447) in pNIC-Bio3 (kindly donated by Finn Wolfreys / Paul Brennan, Target Discovery Institute, Oxford University) was transformed into pBirA-containing Rosetta *E.coli* cells. A single colony was used to inoculate a starter culture in 2TY supplemented with chloramphenicol (34 µg/mL), spectinomycin (50 µg/mL) and kanamycin (30 µg/mL), and grown overnight (37 °C, 160 rpm). The starter culture (1 mL) was diluted 1000-fold in 1 L LB supplemented with spectinomycin (50 µg/mL) and kanamycin (30 µg/mL) and grown (37 °C, 160 rpm). The expression of KDM7B and BirA (biotin ligase) was induced with 0.5 mM IPTG at OD 600nm ~1.00 and the media was further supplemented with 0.5 mM biotin solution. Cells were further grown for 18 hrs (20 °C, 160 rpm) and harvested (4,000 g at 4 °C). Cell pellets were stored at -80 °C. Cell pellets were thawed and re-suspended in 100 mL binding buffer (500 mM NaCl, 5% Glycerol, 50 mM HEPES pH 7.5, 5 mM Imidazole) containing DNaseI and PMSF, 1 mM $MgCl_2$, then lysed by sonication. Cell debris was removed by centrifugation (10,000 g, 4 °C, 35 min) and the supernatant was filtered (0.45 µm) and loaded onto a 5 mL HP His-Trap column on an AKTA Purifier (GE Healthcare). The column was washed with binding buffer (3 CV) and further washed with wash buffer (500 mM NaCl, 5% Glycerol, 50 mM HEPES pH 7.5, 30 mM Imidazole) (3 CV). His-tagged KDM7B was eluted with increasing imidazole gradient using elution buffer (500 mM NaCl, 5% Glycerol, 50 mM HEPES pH 7.5, 250 mM Imidazole). KDM7B containing fractions were analysed by SDS-PAGE and pooled and concentrated to 14 mg/mL. The concentrated protein was treated with TEV protease (at 50:1 protein:TEV mg) at 4 °C overnight for cleavage of the thioredoxin and HisTag, confirmed by SDS-PAGE, before re-application to the HisTrap. The unbound flowthrough containing KDM7B was collected and buffer exchanged (10 mM HEPES pH 7.5, 500 mM NaCl, 5% glycerol), concentrated to 2 mg/mL and aliquots snap frozen in LN_2. Biotinylation was confirmed by protein ESI-MS and confirmation of loading to streptavidin magnetic beads.

Expressed Protein Sequences

Expression Plasmids: pNH-TrxT (N-terminal His_6+Thioredoxin), pNIC-ZB (N-terminal His_6+ZBasic), pNIC-Bio3 (N-terminal His_6 and C-terminal AviTag), pGTVL2 (N-terminal His_6+GST), pNIC-CTHF (C-terminal His_6). TEV Recognition site = ENLYFQS. Cleavage occurs prior to the Serine.

KDM7A PHD. Expression Plasmid: pNH-TrxT. AA: 38-98 HS(Q6ZMT4-1):
MHHHHHHSSGMSDKIIHLTDDSFDTDVLKADGAILVDFWAEWCGPCKMIAPILDEIADEYQGKLTVAKLNIDQNP
GTAPKYGIRGIPTLLLFKNGEVAATKVGALSKGQLKEFLDANLAGTENLYFQSMVYCVCRQPYDVNRFMIECDIC
KDWFHGSCVGVEEHHAVDIDLYHCPNCAVLHGSSLMKKRR

KDM7A PHD+JmjC. Expression Plasmid: pNH-TrxT. AA: 38-480 HS(Q6ZMT4-1):
MHHHHHHSSGMSDKIIHLTDDSFDTDVLKADGAILVDFWAEWCGPCKMIAPILDEIADEYQGKLTVAKLNIDQNP
GTAPKYGIRGIPTLLLFKNGEVAATKVGALSKGQLKEFLDANLAGTENLYFQSMVYCVCRQPYDVNRFMIECDIC
KDWFHGSCVGVEEHHAVDIDLYHCPNCAVLHGSSLMKKRRNWHRHDYTEIDDGSKPVQAGTRTFIKELRSRVFPS
ADEIIIKMHGSQLTQRYLEKHGFDVPIMVPKLDDLGLRLPSPTFSVMDVERYVGGDKVIDVIDVARQADSKMTLH
NYVKYFMNPNRPKVLNVISLEFSDTKMSELVEVPDIAKKLSWVENYWPDDSVFPKPFVQKYCLMGVQDSYTDFHI
DFGGTSVWYHVLWGEKIFYLIKPTDENLARYESWSSSVTQSEVFFGDKVDKCYKCVVKQGHTLFVPTGWIHAVLT
SQDCMAFGGNFLHNLNIGMQLRCYEMEKRLKTPDLFKFPFFEAICWFVAKNLLETLKELREDGFQPQTYLVQGVK
ALHTALKLWMKKELVSEHAFEIPDNVRPGHLIKELSKVIRAIEEENG

KDM7B PHD. Expression Plasmid: pNH-TrxT. AA: 1-66 HS(Q9UPP1-2):
MHHHHHHSSGMSDKIIHLTDDSFDTDVLKADGAILVDFWAEWCGPCKMIAPILDEIADEYQGKLTVAKLNIDQNP
GTAPKYGIRGIPTLLLFKNGEVAATKVGALSKGQLKEFLDANLAGTENLYFQSMASVPVYCLCRLPYDVTRFMIE
CDMCQDWFHGSCVGVEEEKAADIDLYHCPNCEVLHGPSIMKKRR

KDM7B PHD+JmjC. Expression Plasmid: pNH-TrxT. AA: 1-447 HS(Q9UPP1-2):
MHHHHHHSSGMSDKIIHLTDDSFDTDVLKADGAILVDFWAEWCGPCKMIAPILDEIADEYQGKLTVAKLNIDQNP
GTAPKYGIRGIPTLLLFKNGEVAATKVGALSKGQLKEFLDANLAGTENLYFQSMASVPVYCLCRLPYDVTRFMIE
CDMCQDWFHGSCVGVEEEKAADIDLYHCPNCEVLHGPSIMKKRRGSSKGHDTHKGKPVKTGSPTFVRELRSRTFD
SSDEVILKPTGNQLTVEFLEENSFSVPILVLKKDGLGMTLPSPSFTVRDVEHYVGSDKEIDVIDVTRQADCKMKL
GDFVKYYYSGKREKVLNVISLEFSDTRLSNLVETPKIVRKLSWVENLWPEECVFERPNVQKYCLMSVRDSYTDFH
IDFGGTSVWYHVLKGEKIFYLIRPTNANLTLFECWSSSSNQNEMFFGDQVDKCYKCSVKQGQTLFIPTGWIHAVL
TPVDCLAFGGNFLHSLNIEMQLKAYEIEKRLSTADLFRFPNFETICWYVGKHILDIFRGLRENRRHPASYLVHGG
KALNLAFRAWTRKEALPDHEDEIPETVRTVQLIKDLAREIRLVEDIFQQN

KDM7B PHD+JmjC with AviTag. Expression Plasmid: pNIC-Bio3. AA: 1-447 HS(Q9UPP1-2).
MHHHHHHSSGVDLGTENLYFQSMSDKIIHLTDDSFDTDVLKADGAILVDFWAEWCGPCKMIAPILDEIADEYQGK
LTVAKLNIDQNPGTAPKYGIRGIPTLLLFKNGEVAATKVGALSKGQLKEFLDANLAGTENLYFQSMASVPVYCLC
RLPYDVTRFMIECDMCQDWFHGSCVGVEEEKAADIDLYHCPNCEVLHGPSIMKKRRGSSKGHDTHKGKPVKTGSP
TFVRELRSRTFDSSDEVILKPTGNQLTVEFLEENSFSVPILVLKKDGLGMTLPSPSFTVRDVEHYVGSDKEIDVI
DVTRQADCKMKLGDFVKYYYSGKREKVLNVISLEFSDTRLSNLVETPKIVRKLSWVENLWPEECVFERPNVQKYC
LMSVRDSYTDFHIDFGGTSVWYHVLKGEKIFYLIRPTNANLTLFECWSSSSNQNEMFFGDQVDKCYKCSVKQGQT
LFIPTGWIHAVLTPVDCLAFGGNFLHSLNIEMQLKAYEIEKRLSTADLFRFPNFETICWYVGKHILDIFRGLREN
RRHPASYLVHGGKALNLAFRAWTRKEALPDHEDEIPETVRTVQLIKDLAREIRLVEDIFQQNSSKGGYGLNDIFE
AQKIEWHE

KDM7C PHD. Expression Plasmid: pNH-TrxT. AA: 1-67 HS(O75151):
MHHHHHHSSGMSDKIIHLTDDSFDTDVLKADGAILVDFWAEWCGPCKMIAPILDEIADEYQGKLTVAKLNIDQNP
GTAPKYGIRGIPTLLLFKNGEVAATKVGALSKGQLKEFLDANLAGTENLYFQSMATVPVYCVCRLPYDVTRFMIE
CDACKDWFHGSCVGVEEEEAPDIDIYHCPNCEKTHGKSTLKKKRT

DIDO1 PHD. Expression Plasmid: pNH-TrxT. AA: 260-325 HS(Q9BTC0-4):
MHHHHHHSSGMSDKIIHLTDDSFDTDVLKADGAILVDFWAEWCGPCKMIAPILDEIADEYQGKLTVAKLNIDQNP
GTAPKYGIRGIPTLLLFKNGEVAATKVGALSKGQLKEFLDANLAGTENLYFQSMECEGYDPNALYCICRQPHNNR
FMICCDRCEEWFHGDCVGISEARGRLLERNGEDYICPNCTILQVQQ

Protein Mass Spectrometry

Low resolution positive ion electrospray ionisation mass spectrometry was used to analyse intact protein masses using the instrumental set-ups as below:

1) LCT Premier XE ionisation (Micromass) mass spectrometer connected to an Acquity Ultra Performance liquid chromatography (UPLC, Waters) system using an Acquity UPLCR BEH300 C18 column at 50 °C. The protein (~ 0.1 mg/mL, 0.5 µL) was injected and eluted using a gradient system from Solvent A (95 % water, 0.1 % (v/v) formic acid) to Solvent B (95 % acetonitrile, 0.1 % (v/v) formic acid): [Time/min, %A, %B] - [0, 95, 5], [8, 5, 95], [8.1, 95, 5], [10, 95, 5].

Parameters: Capillary 3,000 V; Sample cone 35 V; Desolvation 250 °C; Source 80 °C; Cone gas flow 100 L/h; Desolvation gas flow (N2) 400 L/h. Sodium formate was used as the internal calibrant.

2) Xevo GS-XS QToF mass spectrometer connected to an Acquity Ultra Performance liquid chromatography (UPLC, Waters) system using a ProSwift RP-1S Analytical Phenyl column (Thermo Fisher). The protein (~ 1 µM, 5 µL) was injected and eluted using a gradient system from Solvent A (100 % water, 0.1 % (v/v) formic acid) to Solvent B (100 % acetonitrile, 0.1 % (v/v) formic acid): [Time/min, %A, %B] - [0, 95, 5], [1, 95, 5], [7, 5, 95], [8, 5, 95], [8.1, 95, 5], [10, 95, 5].

Parameters: Source 3,000 V; Cone 30 V; Source 100 °C; Desolvation 350 °C; Cone gas flow 30 L/h; Desolvation gas flow (N2) 600 L/h. A standard solution of Lecuine EnK (4 µg/mL) was used as the internal lock-spray calibrant.

3) Waters Xevo G2-XS Quadrupole Time-of Flight MS connected to a Waters Acquity UPLC i-Class, using a Protein BEH C_4 (2.1 x 150 mm; 1.7 µm particle size) column. The protein (~ 0.5 mg/mL, 3 µL) was injected and eluted using a gradient system from Solvent A (100 % water, 0.1 % (v/v) formic acid) to Solvent B (100 % methanol, 0.1 % (v/v) formic acid): [Time/min, %A, %B] - [0, 95, 5], [0.5, 95, 5], [5, 5, 95], [8, 5, 95], [10, 95, 5], [15, 95, 5] at a flow rate of 0.2 mL/min.

Parameters: Positive ESI; Mass-range 100-2000 Da; Scan rate 1/s; Capillary voltage 3.0 kV; Cone voltage 40 V; Source Temp 110 °C; Desolvation gas temp 300 °C; Cone gas 50 L/h; Desolvation gas 60 L/h; Collision energy 15 eV. Calibration with NaI and lock-mass reference of Leu-Enk.

Spectra were processed using MassLynx v4.0 and v4.1 (Waters) with the Maximum Entropy method (MaxEnt1), or alternatively UniDec was used for deconvolution.

**Protein**	**Calculated Mw**	**Observed Deconvolution**
KDM7A PHD	21273	21272
KDM7A PHD (cleaved)	7347	7346
KDM7B PHD	21655	21654
KDM7B PHD (cleaved)	7728	7727
KDM7C PHD	21713	21712
KDM7C PHD (cleaved)	7786	7785
KDM7A PHD+JmjC	65533	65532
KDM7B PHD+JmjC	65547	65545
KDM7B PHD+JmjC+biotin	54237	54240
KDM7B PHD (15N, 13C) (cleaved)	8149<br>(fully labelled)	8134<br>(>96 % labelled)
KDM7B PHD (15N) (cleaved)	7818<br>(fully labelled)	7816<br>(>98 % labelled)
DIDO PHD	21975	21975

Table S1. Protein mass spectrometry of expressed and purified recombinant proteins. All plasmids used in production of these proteins were verified by DNA sanger sequencing.

Protein NMR

Nuclear Magnetic Resonance (NMR) spectra were recorded using a Bruker AVIII 700 MHz NMR spectrometer equipped with a 5-mm inverse TCI cryoprobe using 3 mm MATCH NMR tubes (Cortectnet, or Hilgenberg, #2001724). Buffering was with 25 mM phosphate buffer, 100 mM NaCl, in 90% H_2O, 10% (v/v) D_2O, pH 6.3 and sample temperatures were regulated at 308 K, except where otherwise specified (e.g. for variable temperature studies). Compounds were added from a concentrated stock in DMSO-d_6, such that final DMSO ≤ 5% (v/v), with ratios noted in respective figure legends. Data were processed with Bruker Topspin 4.0.8 software.

Backbone Assignments:

Data was collected using ^{15}N,^{13}C-labelled KDM7B PHD: protein only sample used 90 μM protein, and the KDM7B PHD with OC9 used 200 μM protein with OC9 at 1 mM. The following experiments were acquired to assign the backbone amide NH peaks in the protein sequence as well as the C', Cα and Cβ peaks: ^1H-^{15}N HSQC, HNCO, HNCA, HN(CO)CA, HN(CA)CO and CbCaCONH. For HSQC, the Bruker pulse sequence *hsqcetfpf3gpsi* was used, with a free induction decay (FID) size of 2048 points (^1H) and 128 points (^{15}N), a spectral width of 16 ppm (^1H) and 35 ppm (^{15}N), and 8 scans. For HNCO, the Bruker pulse sequence *hncogp3d* was used, with an FID size of 2048 points (^1H), 256 points (^{15}N) and 512 points (^{13}C), a spectral width of 14 ppm (^1H), 35 ppm (^{15}N) and 14 ppm (^{13}C), and 8 scans. For HNCA, the Bruker pulse sequence *hncagp3d* was used, with an FID size of 2048 points (^1H), 40 points (^{15}N) and 128 points (^{13}C), a spectral width of 16 ppm (^1H), 35 ppm (^{15}N) and 30 ppm (^{13}C), and 8 scans. For HN(CA)CO, the Bruker pulse sequence *hncacogp3d* was used, with an FID size of 2048 points (^1H), 40 points (^{15}N) and 128 points (^{13}C), a spectral width of 14 ppm (^1H), 35 ppm (^{15}N) and 14 ppm (^{13}C), and 8 scans. For HN(CO)CA, the Bruker pulse sequence *hncocagp3d* was used, with an FID size of 2048 points (^1H), 40 points (^{15}N) and 128 points (^{13}C), a spectral width of 16 ppm (^1H), 35 ppm (^{15}N) and 30 ppm (^{13}C), and 8 scans. For CACB(CO)NH, the Bruker pulse sequence *cbcaconhgp3d* was used, with an FID size of 2048 points (^1H), 40 points (^{15}N) and 128 points (^{13}C), a spectral width of 14 ppm (^1H), 35 ppm (^{15}N) and 80 ppm (^{13}C), and 16 scans. Data analyses were performed using the Analysis programme of the *Collaborative Computing Project for NMR* (CCPNMR) v2[6] running on the *NMRBox* virtual machine software[7].

Variable Temperature Studies:

3-(Trimethylsilyl)propionic-2,2,3,3-d4 acid sodium salt (TSP) was used as a reference point for ^1H chemical shifts and is assumed to be temperature independent. The KDM7B PHD sample used 90 μM ^{15}N-labelled protein with 10 μM TSP. OC9 was added at 450 μM. For each, eleven ^1H-^{15}N HSQC spectra were collected from 288 K to 308 K at 2 K increments. The Bruker pulse sequence *hsqcetfpf3gpsi* was used with an FID size of 2048 points (^1H) and 256 points (^{15}N), a spectral width of 16 ppm (^1H) and 80 ppm (^{15}N), and 8 scans.

Chemical shift perturbations:

CSP Δδ values between free and complexed protein were calculated for amide proton and nitrogen shifts according to:

$$CSP\ \Delta\delta = \sqrt{\frac{1}{2}[(\Delta\delta_H)^2 + (0.14 \cdot \Delta\delta_N)^2]}$$

Where $\Delta\delta_H$ and $\Delta\delta_N$ are differences in chemical shifts between the free and complexed forms for ^1H and ^{15}N respectively.

Peptide variant titrations:

KDM7B PHD sample used 60-90 µM ^{15}N labelled protein with 1 µM DSS (sodium 2,2-dimethyl-2-silapentane-5-sulfonate) and peptide stock solutions of 17 mM in d_6-DMSO were diluted to 6 mM using phosphate buffer for use in titrations.

2D NMR for protein and peptide sidechain studies:

Protein samples (240 µM final concentration) were in phosphate buffer and OC9 (240 µM final concentration) was added. $^{1}H,^{1}H$-TOCSY experiments were collected using a modified version of the Bruker pulse sequence *dipsi2esgpph* to include ^{15}N decoupling in both dimensions for use with the ^{15}N labelled protein samples. This was achieved through incorporating a ^{15}N 180° pulse at the midpoint of t_1 and by using ^{15}N GARP decoupling during ^{1}H acquisition. A data size of 2048 x 256 points and a spectral width of 13 ppm for both dimensions were used, with 32 scans per increment. Mixing times were 45 or 90 ms. For ^{1}H-^{15}N HSQC-TOCSY experiments, the Bruker pulse sequence *hsqcdietf3gpsi* was used, with a data size of 2048 (^{1}H) x 160 points (^{15}N), and spectral widths of 16 ppm (^{1}H) and 26 ppm (^{15}N). 128 scans were collected per increment and mixing times were 30 and 90 ms. $^{1}H,^{1}H$-NOESY experiments used the Bruker pulse sequence *noesyesgpph* again modified to include ^{15}N decoupling in both dimensions, as for TOCSY above. A data size of 2048 x 400 points and a spectral width of 13 ppm for both dimensions were used. 80 scans were acquired per increment with a mixing time of 150 ms. For ^{1}H-^{15}N HSQC-NOESY experiments, the Bruker pulse sequence *hsqcetf3gpno* was used, with a data size of 2048 points (^{1}H) x 256 points (^{15}N), spectral widths of 16 ppm (^{1}H) and 35ppm (^{15}N) with 64 scans per increment. A mixing time of 150 ms was employed. All spectra were referenced to internal DSS at 0.00 ppm.

2D NMR for bound OC9 assignments:

1H Chemical shifts assignments for bound OC9 were obtained from 2D TOCSY and NOESY spectra collected using double F1 and F2 ^{13}C and ^{15}N filtered sequences (A.L. Breeze, Prog. NMR Spectrosc. 2000, 36, 323-372) (Bruker pulse programs: dipsi2gpphwgxf and noesygpphwgxf.2). In these, protons bound to ^{13}C or ^{15}N nuclei are filtered (removed) from the resulting spectra, such that only resonances derived from components that are lacking these isotope labels remain visible. These 2D experiments were therefore collected using uniformly ^{13}C and ^{15}N labelled KDM7B PHD-finger (240 µM) complexed with ~0.9 equiv. unlabelled OC9 (200 µM) in 25 mM phosphate, 10 mM NaCl at pH 6.3 with 10 % D_2O at 308 K, prepared in 180 µL (3 mm tube). Filtered TOCSY and NOESY spectra were collected with data sizes of 2048 x 256 points and spectral widths of 14 ppm for both ^{1}H dimensions centred at 4.7 ppm, and ^{13}C and ^{15}N offsets of 43 ppm (O2P) and 117 ppm (O3P) respectively. Mixing times were 80 ms and 150 ms for TOCSY and NOESY respectively, with 32 (TOCSY) or 64 (NOESY) transients per increment. Data were collected on a Bruker AVIII 700 equipped with a 5 mm TCI cryoprobe.

Peptide Synthesis

General Procedures

All histone $H3_{1-21}$ peptides were purchased from GL Biochem (Shanghai) (>95% purity), received as solid and dissolved to stock solutions into H_2O or $DMSO_{d6}$, with mass identity verified by MALDI-TOF-MS. Biotinylated histone peptides were purchased from AnaSpec, with a GGK-biotin unit at the *C*-terminus of the sequence.

All other peptides were synthesised from the C-terminus, using solid phase peptide synthesis (SPPS) on either Liberty Blue (CEM, microwave assisted synthesis) or Chorus (Gyros Protein Technologies, induction heating) on 0.1 or 0.05 mmol scale. Rink Amide MBHA resin (100-200 mesh) was purchased from AGTC Bioproducts or Sigma Aldrich. All N-fluorenylmethyloxycarbonyl (Fmoc) protected L-amino acids and non-canonical amino acids were purchased from CEM, Sigma Aldrich, Novabiochem or Fluorochem with protected side-chains for the following amino acids: Trt (Asn, Cys, Gln, His), tBu (Asp, Glu, Ser, Thr, Tyr), Boc (Lys, Trp), Pbf (Arg). *N,N*-dimethylformamide (DMF, Ultra Pure, Cambridge Reagents or peptide grade, Sigma Aldrich, Alfa Aesar) was used throughout.

<u>Rink amide resin loading:</u> Rink Amide MBHA resin (1.0 equiv) was pre-swelled in DMF or DMF/DCM for at least 15 min, then washed with DMF. Fmoc-deprotection of resin was performed using 20 % (v/v) piperidine in DMF (3 mL) at 50 °C for 2-3 minutes, and repeated once more. The resin was washed with DMF (3 x 3 mL).

<u>Iterative Fmoc amino acid coupling and deprotection:</u> The activating agent diisopropylcarbodiimide (DIC, Fluorochem) and activating base Oxyma (Novabiochem) (sometimes with N,N-diisopropylethylamine additive (DIPEA, Sigma Aldrich)) were used to couple Fmoc-amino acids (AAs) in 1:1:1 ratio in DMF, with a final concentration of amino acids in the reaction typically ~83 mM. Synthesis was typically performed with 5-fold excess of reagents over the molar amount of resin, using a single coupling cycle at 90 °C for 2 minutes as standard, but 50 °C and 10 minutes for cysteine or histidine, and a 75 °C double coupling for arginine. The resin was washed with DMF (3 x 3 mL) and Fmoc deprotected using 20 % (v/v) piperidine in DMF, typically at 50 °C for 2 minutes, performed twice for each coupling. The resin was further washed with DMF (3 x 3 mL) prior to the next coupling step.

<u>Chloroacetylation of N-terminus of peptides:</u> For peptides synthesised on Liberty Blue, peptide-on-resin was manually suspended in ~5 mL of DMF and treated with 10 equivalents of either chloroacetic anhydride (Sigma Aldrich) or acetic anhydride for 2 hours at 20 °C with manual agitation every 30 minutes and a further 5 equivalents added for 1 hour. The resin was subsequently washed in a phase separator (TELOS, 25 mL) under reduced pressure with DMF (10 mL), DCM (3x 20 mL) and dried to a free-flowing texture under reduced pressure. For peptides synthesised on Gyros, chloroacetic anhydride treatment with 10-fold excess in DMF was performed on-machine for 30 minutes at 60 °C.

<u>Cleavage and workup / purification:</u> Cleavage and removal of side-chain protection groups was performed with ~4 mL of a cleavage cocktail TFA mix (trifluoroacetic acid (TFA, 92.5% v/v), triisopropylsilane (TIPS, 2.5% v/v), 1,3-dimethoxybenzene (2.5% v/v) and H_2O (2.5% v/v)) at room temperature (~20°C) for 3 hours. Peptides were precipitated by addition of ~40 mL ice cold diethyl ether (Et_2O), followed by centrifugation at 3000 g for 5 minutes and repeating the ether wash a further four times.

<u>Peptide cyclisation:</u> Thioether cyclisation was performed either in: i) DMSO with triethylamine added to pH ~8/9 and incubation at 37°C for 60 minutes, or ii) in MeCN/H_2O with 0.1 M NaOH added dropwise to pH ~8/9 and incubation at 60°C for 60 minutes, or iii) in 20 mM ammonium bicarbonate in MeCN/H_2O at pH 8 for 60 minutes at 40 °C with sonicator agitation. MALDI-TOF-MS was used to confirm cyclisation. Reaction mixtures were then either diluted with MeCN/H_2O (0.1 % TFA) for purification by HPLC, or in the case of bicarbonate solution cyclisations they were freeze dried before re-dissolution in appropriate solvent. All cyclisation conditions gave the same product, with the same activity (as confirmed by MS and NMR, and different batches of OC9 yielding same binding response (K_D) against KDM7B PHD).

General Peptide Purification and Characterisation

Peptides were purified using a JASCO or Agilent 1260 HPLC system with a Phenomenex Gemini-NX 5 µm C18 110 Å 250x30 mm column, UV Detection at 220 nm. Typically, a solvent system of A = H_2O, 0.1 % (v/v) TFA and B = MeCN, 0.1 % (v/v) TFA, was used with increasing B gradient elution, customised for each peptide, over 45-60 minutes. Product containing fractions were identified by MALDI-TOF-MS, with those corresponding to a single peak combined and lyophilized to a white powder, and identity confirmed by high resolution ESI-MS. Analytical HPLC of purified samples was run on an Agilent 1220 system with a Phenomenex BioZen Peptide XB-C18 2.6 µm, 150 x 4.6 mm column or Phenomenex BioZen Peptide PS-C18 3 µm, 150 x 4.6 mm column, using a 2-98 % gradient of MeCN (analytical grade) in MilliQ water, with 0.1 % TFA (analytical grade).

Peptide concentrations were calibrated by ^1H-NMR in D_2O or DMSO-d_6 comparing integrals of characteristic peaks to an internal standard of TSP (3-(Trimethylsilyl)propionic-2,2,3,3-d4 acid sodium salt) (Sigma Aldrich), using a Bruker 500 MHz Avance II with BBO Probe or Avance III with TBO probe.

Detailed Synthesis Procedure for OC9 on Chorus

A 50 µmol scale synthesis of linear OC9 (ClAc-YYLTHPYPSVFRTC-NH_2) was performed on rink amide resin (1.0 equiv, 96 mg, ~0.52 mmol/g loading, 100-200 mesh, Sigma Aldrich) pre-swelled in DMF on-machine in 10 mL glass reaction vials. Amino acid loading / coupling reactions used DIC (5 equiv), Oxyma (5 equiv) and Fmoc-amino acid (5 equiv) in DMF (3 mL); the coupling reagents were prepared at 250 mM stock in DMF. Deprotection reactions for Fmoc removal were performed using 20 % (v/v) piperidine in DMF (3 mL) and repeated twice for each coupling (method below). The Chorus settings for a 'standard' coupling at 50 µmol scale were as below.

Step	Operation	Solvent	Volume (mL)	Mix Time (MM:SS)	Drain	Reps	N_2	Heat (°C)	Shake (RPM)	Comment
1	Bottom Delivery	20 % piperidine in DMF	3	02:45	Y	2	Y	50	350	Deprotection
2	Top Delivery	DMF	3	00:30	Y	3	Y	-	350	Wash
3	Amino Acid	AA	1	00:00	N	1	N	-	-	AA Addition
4	Bottom Delivery	Oxyma	1	00:00	N	1	N	-	-	Oxyma addition
5	Bottom Delivery	DIC	1	02:45	Y	1	Y	90	350	DIC addition and coupling
6	Top Delivery	DMF	3	00:30	Y	3	Y	-	350	Wash

Note: for a cysteine or histidine coupling, step 5 was altered (time and temp):

Step	Operation	Solvent	Volume (mL)	Mix Time (MM:SS)	Drain	Reps	N_2	Heat (°C)	Shake (RPM)	Comment
5	Bottom Delivery	DIC	1	05:00	Y	1	Y	50	350	DIC addition and coupling

Note: for an arginine coupling, step 5 was altered (time and temp and repetition):

Step	Operation	Solvent	Volume (mL)	Mix Time (MM:SS)	Drain	Reps	N_2	Heat (°C)	Shake (RPM)	Comment
5	Bottom Delivery	DIC	1	04:45	Y	1	Y	75	350	DIC addition and coupling

followed by step 6, followed by steps 3-6 copied and addended (steps 7-10) (double coupling).

Note: the final amino acid coupling had additional end-steps (final deprotection and washes):

Step	Operation	Solvent	Volume (mL)	Mix Time (MM:SS)	Drain	Reps	N_2	Heat (°C)	Shake (RPM)	Comment
7	Bottom Delivery	20 % piperidine in DMF	3	02:45	Y	2	Y	50	350	Deprotection
8	Top Delivery	DMF	3	00:30	Y	3	Y	-	350	Wash

Following final Fmoc-removal on the *N*-terminus, chloroacetic anhydride (ClAc) (~10-fold molar excess to resin, 166 mM stock) was added on the Chorus under the following settings:

Step	Operation	Solvent	Volume (mL)	Mix Time (MM:SS)	Drain	Reps	N_2	Heat (°C)	Shake (RPM)	Comment
1	Amino Acid	ClAc in DMF	3	30:00	Y	1	Y	60	350	Deprotection
2	Top Delivery	DMF	3	00:30	Y	3	Y	-	350	Wash
3	Top Delivery	DCM	3	00:30	Y	5	Y	-	350	Wash

Amino acid protecting group removal and simultaneous cleavage from the resin was performed on the Chorus, using a cleavage cocktail: trifluoroacetic acid (TFA, 92.5% v/v), triisopropylsilane (TIPS, 2.5% v/v), 1,3-dimethoxybenzene (2.5% v/v) and H_2O (2.5% v/v)), with the following program:

Step	Operation	Solvent	Volume (mL)	Mix Time (HH:MM:SS)	Drain	Reps	N_2	Heat (°C)	Shake (RPM)	Comment
1	Top Delivery	DCM	3	00:00:30	Y	5	N	-	350	Wash
2	Drain / Dry	-	-	00:10:00	Y	1	N	-	-	Drying
3	Bottom Delivery	TFA mix	3	03:00:00	Y	1	N	-	350	Deprotection and cleavage
4	Collect	-	-	-	N	1	N	-	-	Transfer to collection tube
5	Bottom Delivery	TFA mix	3	00:00:30	Y	1	N	-	350	TFA mix wash transfer
6	Collect	-	-	-	N	1	N	-	-	Transfer to collection tube
7	Top Delivery	DCM	3	00:00:30	Y	5	N	-	350	Wash
8	Drain / Dry	-	-	00:10:00	Y	1	N	-	-	Drying

OC9 was then precipitated off-machine by manual addition of diethyl ether (Sigma Aldrich) (40 mL, total volume ~45 mL in 50 mL falcon collection tube). The solution was mixed and white precipitate was observed. The suspension was pelleted by centrifugation, the supernatant removed to waste and OC9 crude pellet re-suspended in 40 mL of diethyl ether by vigorous vortexing and centrifuged. The pellet washing process was performed five times. The final pellet was air-dried within a fumehood for at least 2 hours, then the pellet was re-dissolved in 10 mL of 1:1 MeCN:H_2O, frozen in liquid nitrogen and lyophilised overnight. Cyclisation of OC9 was performed by dissolution of the freeze-dried pellet in 10 mL of 1:1 MeCN : aqueous ammonium bicarbonate (20 mM final) in a 50 mL falcon tube, at pH 8 (by tri-colour pH paper test), with pH adjusted by dropwise addition of 0.1 M NaOH (aqueous). The suspension was incubated at 40 °C for 60 minutes with sonication in a heated sonicator water bath. Completion of the cyclisation reaction was confirmed by MALDI-TOF-MS analysis. The solution was frozen in liquid nitrogen and lyophilised overnight. The pellet was prepared for purification by dissolution in 5 mL of 15 % MeCN in water with 0.1 % TFA, ensuring pH 1-2, and passed through a syringe filter (0.22 µm). Preparative HPLC (Agilent 1260), was performed with a Gemini 5 µm, NXC18, 110 Å, 250 x 21 mm column with UV monitoring at 220 nm, and automated sampler injection of filtered (0.22 µm syringe filter) peptide solution (900 µL per run). The following gradient was run on the column (pre-equilibrated at the starting % solvent B) with a 20 mL/min flow-rate:

Solvent A: MilliQ H_2O with 0.1 % v/v TFA (HPLC grade), filtered (0.22 µm and de-gassed under vacuum). Solvent B: MeCN (HPLC grade, ThermoFisher) with 0.1 % TFA v/v (HPLC grade). % B at: 0 min = 15; 5 min = 15; 10 min = 25; 30 min = 40; 40 min = 60; 45 min = 95; 50 min = 95; 55 min = 15; 60 min = 15.

The OC9 containing fractions were identified by MALDI-TOF-MS (eluting at 17.2 min), and those of suitable purity from each injection were combined and lyophilised to dryness. Resulting purified pellets were combined by re-dissolution into a total of 500 µL of 1:1 MeCN:H_2O, and transferred to a pre-weighed 1.5 mL Eppendorf, then re-frozen in liquid nitrogen and lyophilised to dryness. This process gave 17.65 mg of a white powder solid (20 % yield, >99 % purity by analytical HPLC).

Azide-Alkyne 'Click' Reaction for OC9-Biotin Synthesis

To a 1.5 mL Eppendorf tube was added 100 µL of 10X Cu(II)SO_4 aqueous solution (0.13 mg, 2 eqv) and 100 µL of 10X tris-hydroxypropyltriazolylmethylamine (THPTA) aqueous solution (1.04 mg, 6 eqv). The mixture was allowed to incubate at 20 °C for several minutes until a light blue colour emerged. Separately, to a 1.5 mL Eppendorf tube was added OC9-Azide (0.95 mg, 1 eqv) in 100 µL of H_2O/MeCN (80/20), followed by 100 µL of Biotin-PEG(4)-Alkyne aqueous solution (0.20 mg, 1.1 eqv) (15-[D(+)-Biotinylamino]-4,7,10,13-tetraoxapentadec-1-yne, CAS# 1262681-31-1) (Source: PEG4950.0250, Iris Biotech GmbH).

The Cu(II)SO_4/THPTA mixture was then added to the OC9-azide/alkyne solution, followed by 100 µL of sodium ascorbate aqueous solution (0.79 mg, 10 eqv) and mixed gently with a pipette whereupon the solution turned clear. The reaction mixture was then buffered by addition of 500 µL of 20 mM sodium phosphate (pH 6.5), the lid sealed and the reaction incubated at 37 °C, 600 rpm on a Thermomixer C (Eppendorf). Concentrations in final reaction were: OC9-Azide (0.4 mM); Biotin-PEG(4)-Alkyne (0.44 mM); Cu(II)SO4 (0.8 mM); THPTA (2.4 mM); Sodium Ascorbate (4 mM). The reaction was monitored by sampling of 1 µL aliquots into 50 µL of H_2O/MeCN (50/50) and spotting of 1 µL for MALDI-TOF-MS analysis. After 6 hours no starting material was detectable by MALDI-TOF-MS, the reaction was deemed complete, and the reaction mixture was injected directly to HPLC for purification (see general HPLC protocol for peptides).

Peptide High-Resolution Mass Spectrometry Method

Samples were prepared by dilution in H_2O/MeCN to < 0.1 mg/mL and run on a Waters Acquity Ultraperformance LC system connected to a Thermo Orbitrap Exactive MS for electrospray in the regime of direct infusion. ESI capillary voltage 3000 V; ion source 200 °C; transfer capillary 350 °C. Elevated temperatures were used during the electrospray for desolvation of the ions and prevention of non-volatile neutral molecules contaminating the ion source.

OR

Waters Xevo G2-XS Quadrupole Time-of Flight MS connected to a Waters Acquity UPLC i-Class, using a Waters - Acquity BEH-C18 (50 x 2.1 mm; 1.7 µm particle size) column. The peptide (~ 0.1 mg/mL, 3 µL) was injected and eluted using a gradient system from Solvent A (100 % water, 0.1 % (v/v) formic acid) to Solvent B (100 % methanol, 0.1 % (v/v) formic acid): [Time/min, %A, %B] - [0, 95, 5], [0.5, 95, 5], [5, 5, 95], [8, 5, 95], [10, 95, 5], [15, 95, 5] at a flow rate of 0.2 mL/min.

Parameters used: Positive ESI; Mass-range 100-2000 Da; Scan rate 1/s; Capillary voltage 3.0 kV; Cone voltage 40 V; Source Temp 110 °C; Desolvation gas temp 300 °C; Cone gas 50 L/h; Desolvation gas 60 L/h; Collision energy 15 eV. Calibration with NaI and lock-mass reference of Leu-Enk.

Peptide High-Resolution Mass Spectrometry Values

Peptide Name		Formula	Mass m/z		Mass error ppm
			Calculated	Observed	
OC3	$[M+H]^+$	C79 H127 O16 N20 S1	1643.94541	1643.93909	-3.85
OC4	$[M+H]^+$	C83 H107 N15 O20 S1	1666.7610	1666.7640	-1.78
OC9	$[M+H]^+$	C85 H117 O21 N20 S1	1785.84174	1785.83655	-2.91
OC9_Biotin	$[M+2H]^{2+}$	C127 H187 O37 N33 S2	1415.15980	1415.15894	-0.61
OC9_Linear	$[M+2H]^{2+}$	C85 H120 O21 N20 S1	894.43233	894.43201	-0.36
OC9_Y2A	$[M+H]^+$	C79 H113 O20 N20 S1	1693.81552	1693.81274	-1.64
OC9_L3A	$[M+H]^+$	C82 H111 O21 N20 S1	1743.79479	1743.79248	-1.32
OC9_T4A	$[M+H]^+$	C84 H115 O20 N20 S1	1755.83117	1755.82849	-1.53
OC9_H5A	$[M+H]^+$	C82 H115 O21 N18 S1	1719.81994	1719.81628	-2.13
OC9_P6A	$[M+H]^+$	C83 H115 O21 N20 S1	1759.82609	1759.82373	-1.34
OC9_Y7A	$[M+H]^+$	C79 H113 O20 N20 S1	1693.81552	1693.81213	-2.00
OC9_P8A	$[M+2H]^{2+}$	C83 H116 O21 N20 S1	880.41668	880.41522	-1.66
OC9_S9A	$[M+H]^+$	C85 H117 O20 N20 S1	1769.84682	1769.84595	-0.49
OC9_V10A	$[M+H]^+$	C83 H113 O21 N20 S1	1757.81044	1757.80896	-0.84
OC9_F11A	$[M+2H]^{2+}$	C79 H114 O21 N20 S1	855.40886	855.40839	-0.55
OC9_R12A	$[M+2H]^{2+}$	C82 H111 O21 N17 S1	850.89251	850.89185	-0.78
OC9_T13A	$[M+H]^+$	C84 H115 O20 N20 S1	1755.83117	1755.82825	-1.67
OC9_Y1F	$[M+2H]^{2+}$	C85 H118 O20 N20 S1	885.42705	885.42664	-0.47
OC9_Y2F	$[M+2H]^{2+}$	C85 H118 O20 N20 S1	885.42705	885.42566	-1.57
OC9_Y7F	$[M+2H]^{2+}$	C85 H118 O20 N20 S1	885.42705	885.42511	-2.19
OC9_Y1F Y2F Y7F	$[M+2H]^{2+}$	C85 H118 O18 N20 S1	869.43214	869.43176	-0.43
OC9_T4A H5A P8A	$[M+2H]^{2+}$	C79 H112 O20 N28 S1	832.40050	832.40143	1.11
OC9_Y1F Y2F T4A H5A Y7F	$[M+2H]^{2+}$	C81 H114 O17 N18 S1	821.41595	821.41614	0.22
OC9_T4-dA	$[M+2H]^{2+}$	C84 H116 O20 N20 S1	878.41922	878.41864	-0.67
OC9_H5-dA	$[M+2H]^{2+}$	C82 H116 O21 N18 S1	860.41361	860.41486	1.45
OC9_P8-dA	$[M+2H]^{2+}$	C83 H116 O21 N20 S1	880.41668	880.41528	-1.59
OC9_P6trans-4F-P	$[M+2H]^{2+}$	C85 H117 O21 N20 F1 S1	902.41980	902.41925	-0.60
OC9_P6cis-4F-P	$[M+2H]^{2+}$	C85 H117 O21 N20 F1 S1	902.41980	902.41882	-1.08
OC9 V10DV	$[M+H]^+$	C85 H117 O21 N20 S1	1785.84174	1785.8352	-3.66
OC9 V10L	$[M+H]^+$	C86 H119 O21 N20 S1	1799.8574	1799.8567	-0.39
OC9 V10K	$[M+2H]^{2+}$	C86 H121 O21 N21 S1	907.9305	907.9389	1.19
OC9 V10Kme3	$[M+2H]^{2+}$	C89 H127 O21 N21 S1	928.9613	928.9637	2.63

Table S2. High-resolution mass spectrometry characterisation data for synthesised peptides

Peptide MALDI-TOF-MS Spectra

OC9

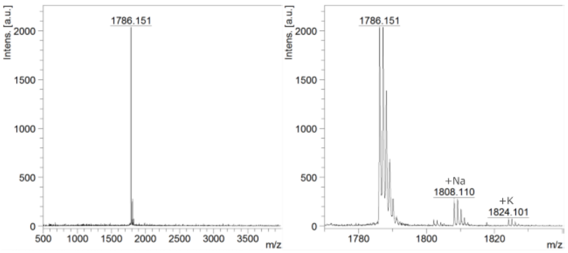

OC9 Linear

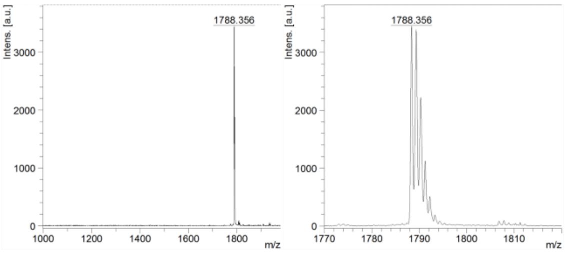

OC9 Y2A

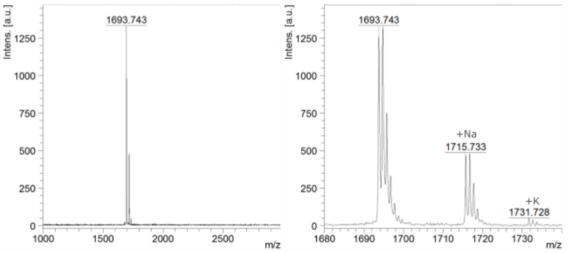

OC9 L3A

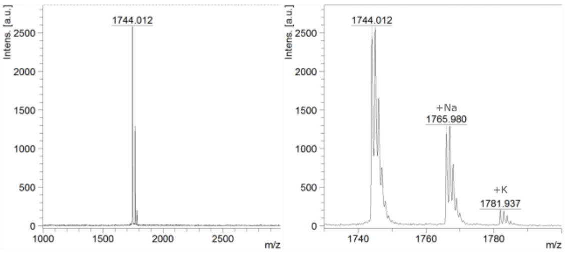

OC9 T4A

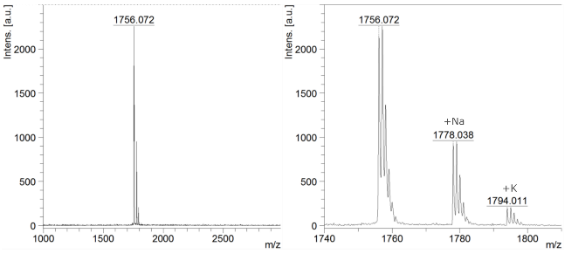

OC9 H5A

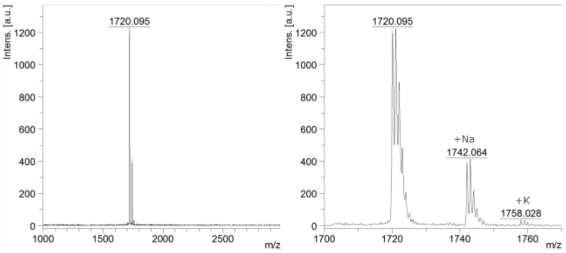

OC9 P6A

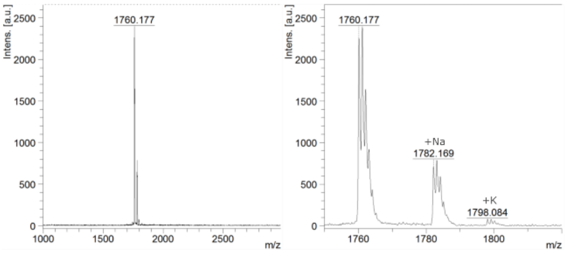

OC9 Y7A

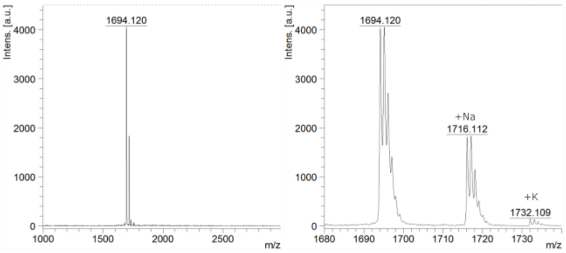

OC9 P8A

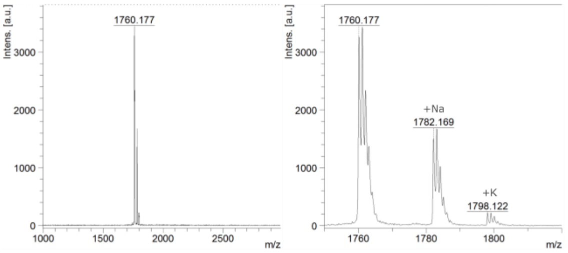

OC9 S9A

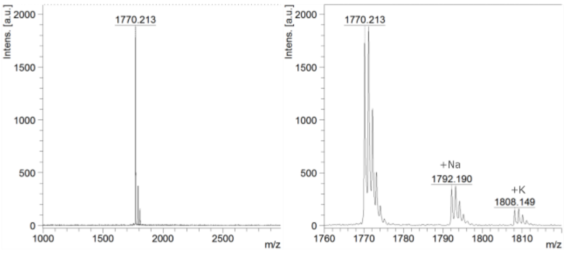

OC9 V10A

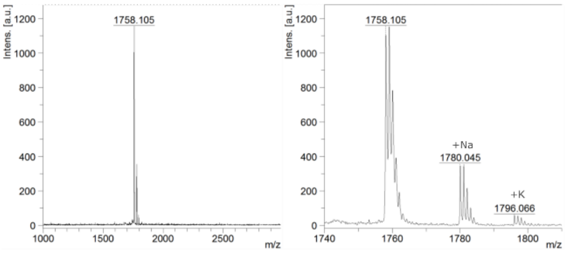

OC9 F11A

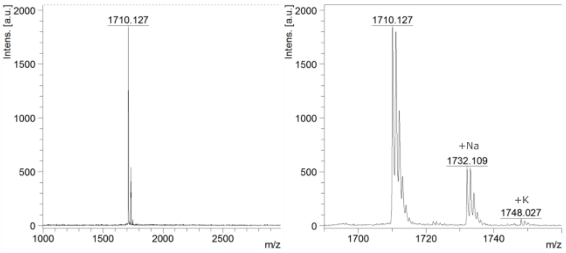

OC9 R12A

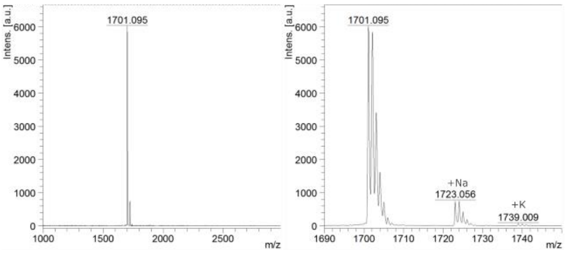

OC9 T13A

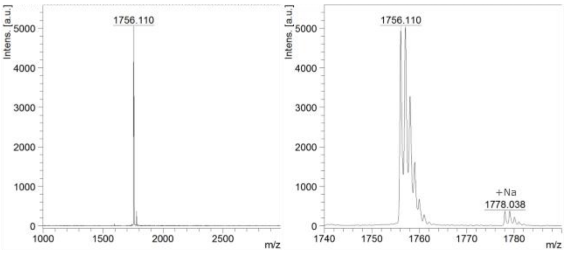

OC9 Y1F

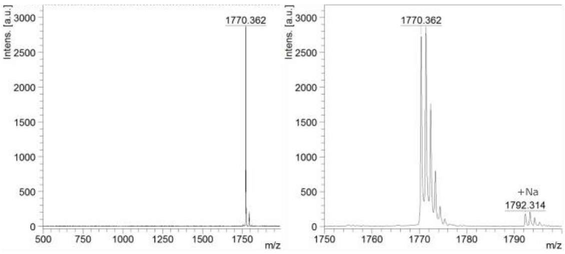

OC9 Y2F

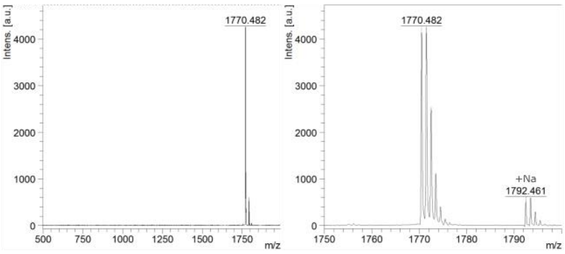

OC9 Y7F

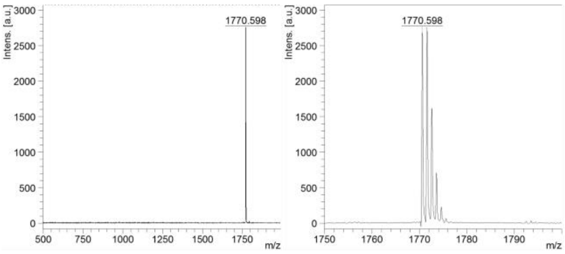

OC9 Y1F Y2F Y7F

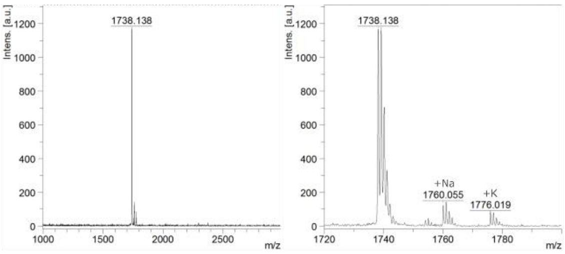

OC9 T4A H5A P8A

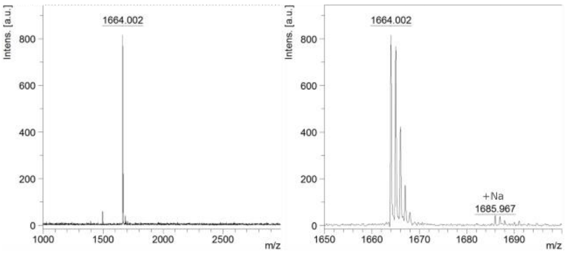

OC9 Y1F Y2F T4A H5A Y7F

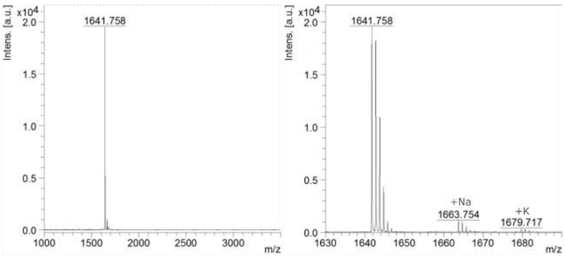

OC9 T4a

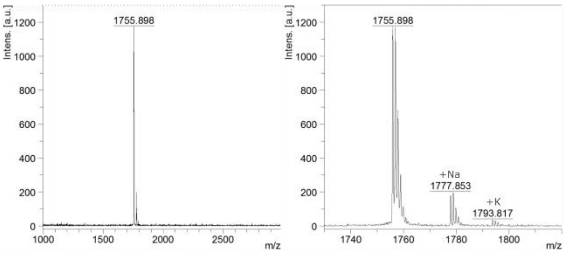

OC9 H5a

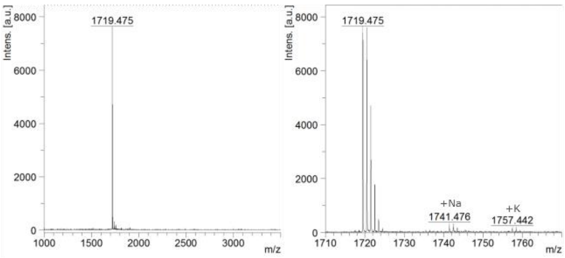

OC9 P8a

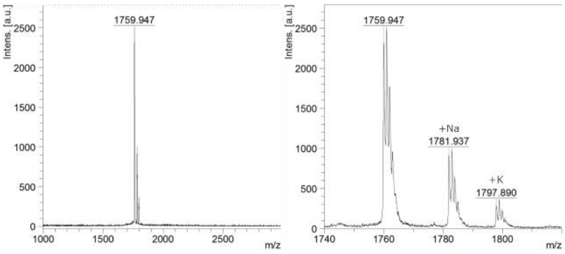

OC9 P6(trans-4-fluoro)P

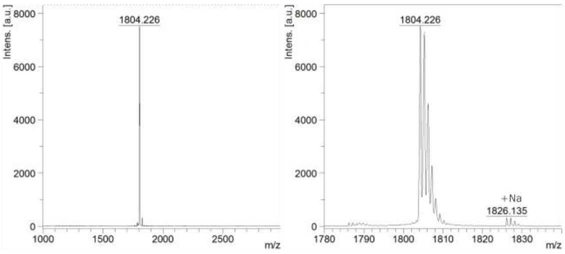

OC9 P6(cis-4-fluoro)P

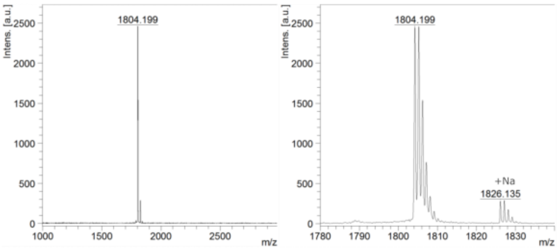

OC9 Azide

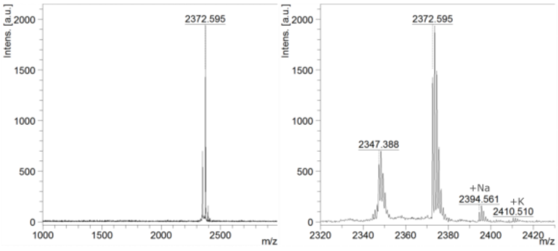

OC9 Biotin

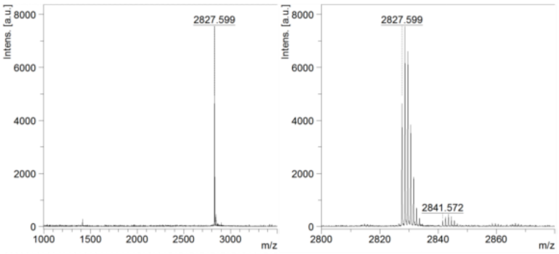

OC9 V10DV

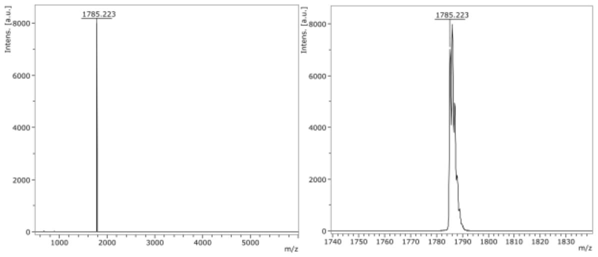

OC3

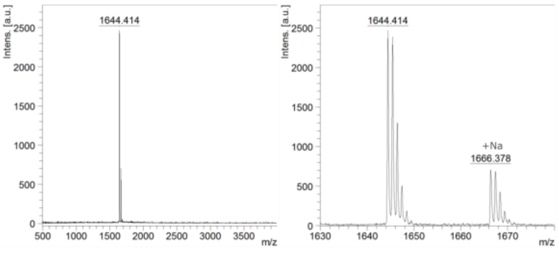

OC4

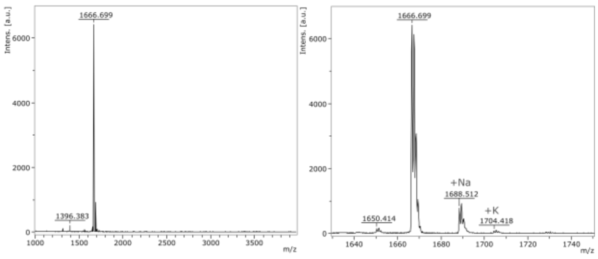

OC9 V10L

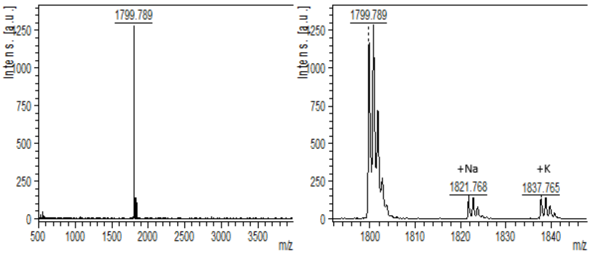

OC9 V10Kme3

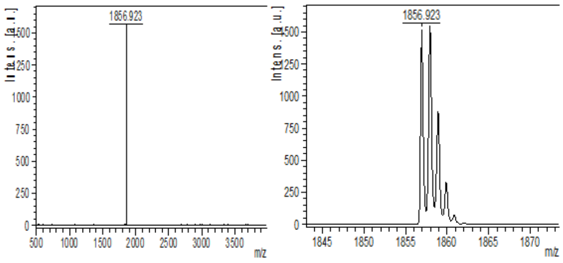

OC9 V10K

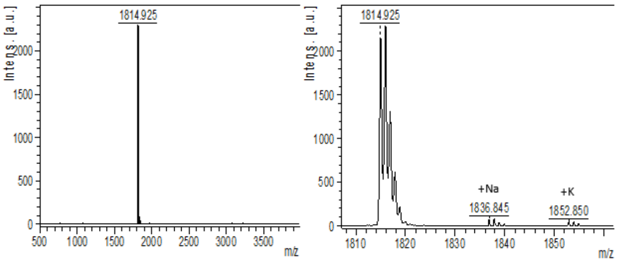

Peptide Analytical HPLC Traces

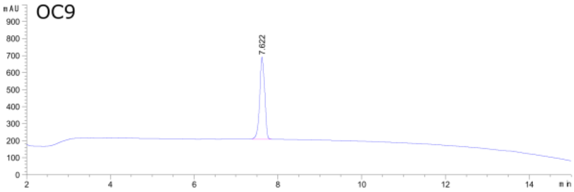

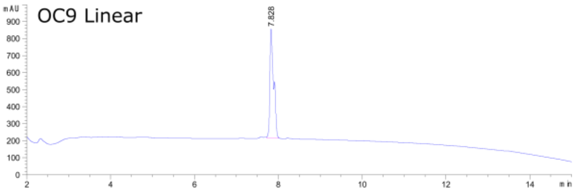

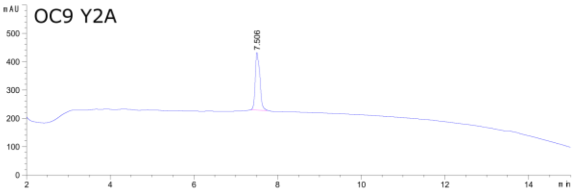

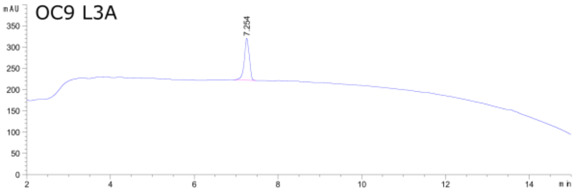

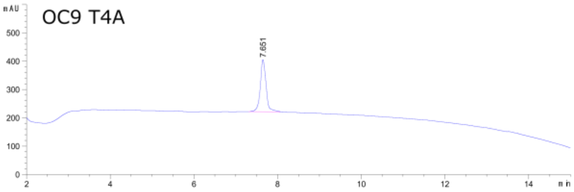

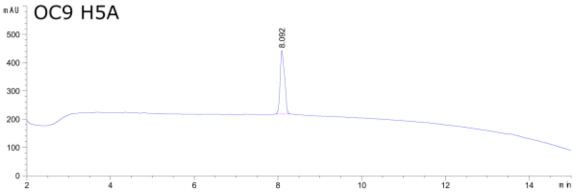

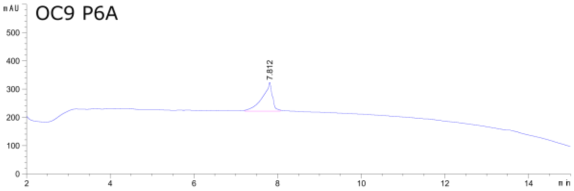

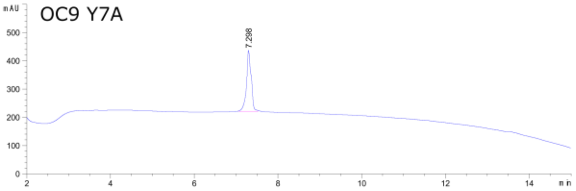

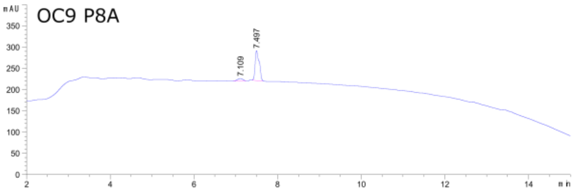

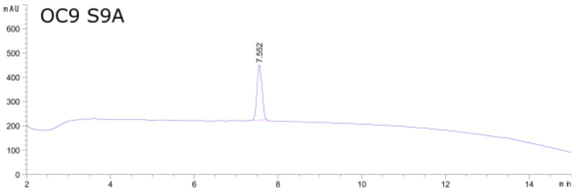

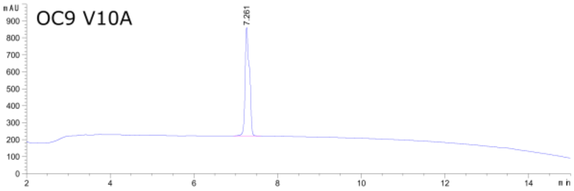

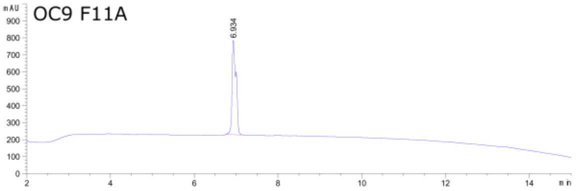

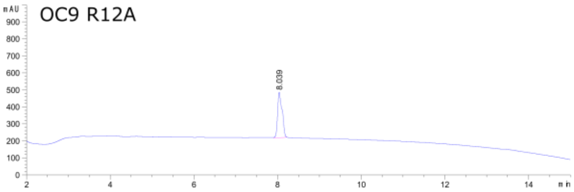

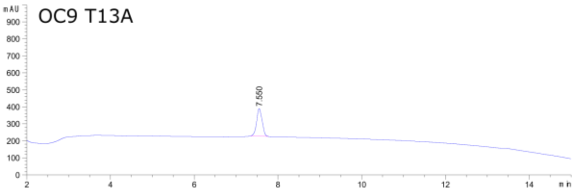

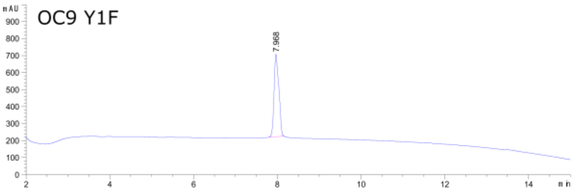
OC9 Y1F
7.968
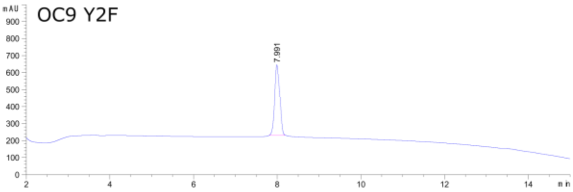
OC9 Y2F
7.991
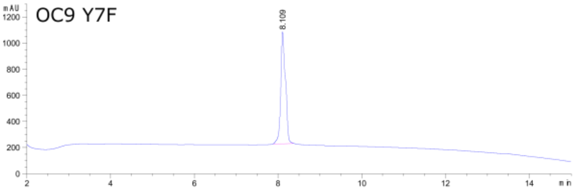
OC9 Y7F
8.109
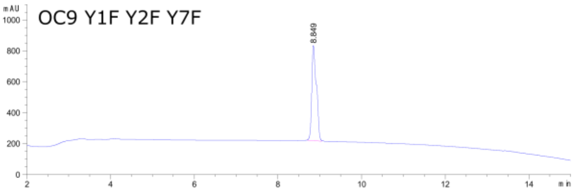
OC9 Y1F Y2F Y7F
8.849
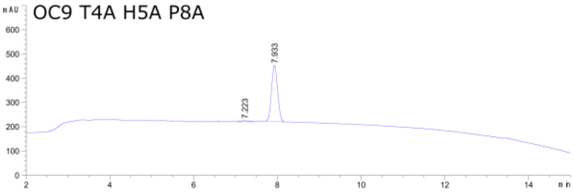
OC9 T4A H5A P8A
7.223
7.933
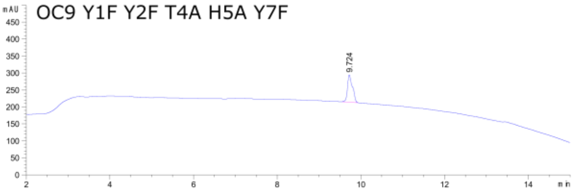
OC9 Y1F Y2F T4A H5A Y7F
9.724
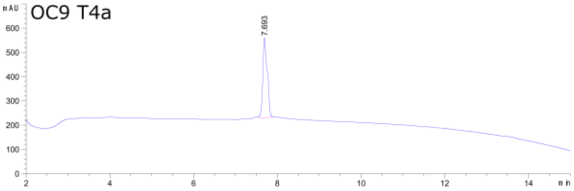
OC9 T4a
7.693
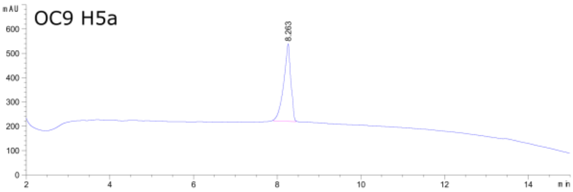
OC9 H5a
8.263
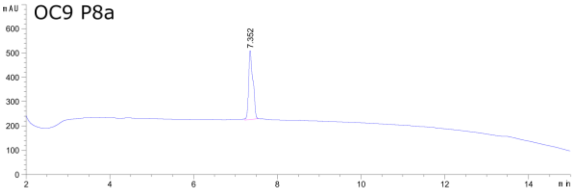
OC9 P8a
7.352
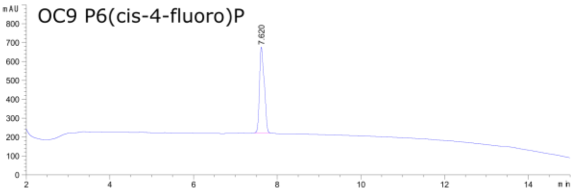
OC9 P6(cis-4-fluoro)P
7.620
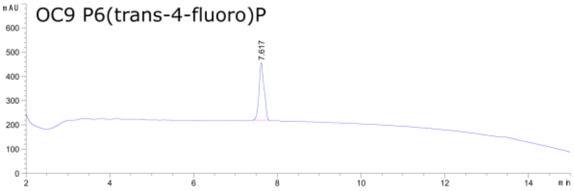
OC9 P6(trans-4-fluoro)P
7.617
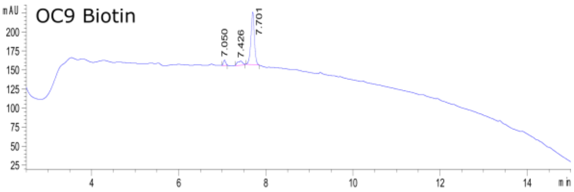
OC9 Biotin
7.050
7.426
7.701
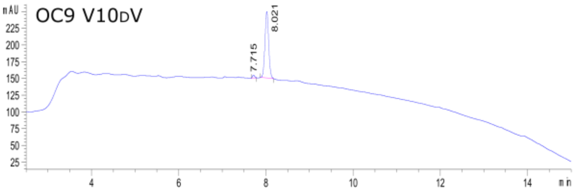
OC9 V10DV
7.715
8.021
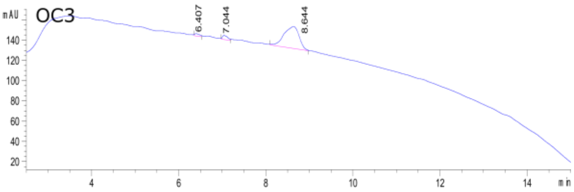
OC3
6.407
7.044
8.644
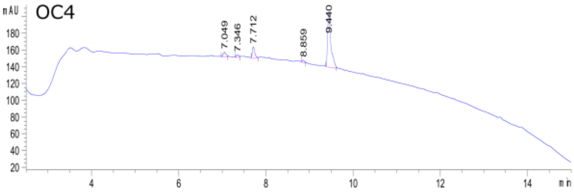
OC4
7.049
7.346
7.712
8.859
9.440
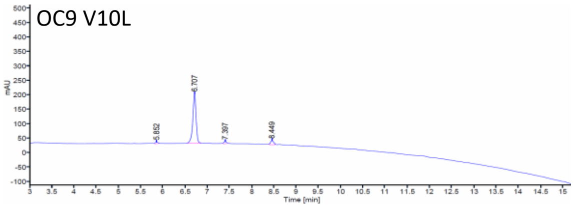
OC9 V10L
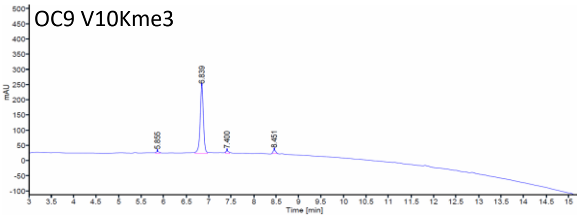
OC9 V10Kme3
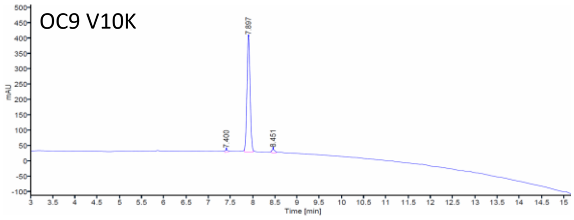
OC9 V10K

BioLayer Interferometry (BLI)

BLI was performed on an OctetRed 384 (Forte Bio) or Octet R16 (Sartorius) system using a 384-well tilted bottom plate (Forte Bio / Sartorius) with Ni-NTA or Streptavidin (SA) biosensors (Forte Bio / Sartorius). Measurements were obtained at 25 °C. The protein of interest with an appropriate immobilisation tag (e.g. His tag, biotin tag) was diluted to ~1 µM in Buffer 1 [1x PBS, 2 mM BME (β-mercaptoethanol)/TCEP (Tris(2-carboxyethyl)phosphine), 0.05 % (v/v) Tween20, 1 or 0.1 % BSA] and kept at 4 °C until use. Compounds were diluted into Buffer 1 from a concentrated stock solution, and the % of DMSO kept constant for further dilutions and dissociation steps. Compounds were typically tested at 7 points of 2-fold dilutions, with a reference 0-peptide well for subtraction of sensor drift as recommended by Forte Bio Technical Note 31. Protein loading was optimised for each protein, typically to 1-3 nm. For Ni-NTA, the biosensors were stripped between compounds by immersion in glycine (0.5 M, pH 1) and regenerated with 10 mM $NiCl_2$ before re-equilibration in buffer and protein re-loading. Data were processed using ForteBio DataAnalysis v9 to apply baseline alignment, inter-step correction if necessary and Savitzky-Golay filtering, then processed data were exported and fitted with an 'Association then Dissociation' 1:1 binding model using GraphPad Prism v8/9 to determine K_D. For equilibrium steady-state analysis, the equilibrium response (R_{obs}) was fitted using 'One site – Total and non-specific binding' model to determine K_D. For SA biosensors, non-specific data were obtained using non-loaded biosensors with compounds.

Isothermal Titration Calorimetry (ITC)

Malvern Panalytical MicroCal PEAQ-ITC Automated was used for isothermal titration calorimetry measurements (cell volume = 200 µL containing protein, cell material = Hastelloy, injection syringe volume = 40 µL containing compound). Samples were prepared in ITC buffer (50 mM HEPES, 100 mM NaCl, pH 7.4, 0.2 µm filtered). Proteins were buffer exchanged immediately prior to use with a PD-MiniTrap G-25 (GE Healthcare). Protein concentrations were measured by taking an average of three Nanodrop readings, with extinction coefficients and mass predicted by ExPASy ProtParam based on the amino acid sequence. Peptides were dissolved from solid powder directly into ITC buffer, with volume of dissolution determined by previous ^{1}H-NMR concentration calibrations. Titration consisted of 19 injections with 1x 0.4 µL (0.8 s), 18x 2 µL (4.0 s), spaced 150 s apart, stirring at 750 rpm and 25 °C, with reference power set between 40-42 µW. Data were analysed using Malvern Panalytical MicroCal PEAQ-ITC Analysis Software 1.1.0.1262, fitted using a one-set-of-sites binding model. Typically, 10 µM protein was used with 100 µM compound.

Cell (Protein) / Syringe (Compound) Concentrations (µM)			
**Protein** (AAs)	**Compound**		
	**OC9**	**$H3_{1\text{-}21}$K4me3**	**OC3**
**KDM7A PHD** (38-98)	10 / 113	10 / 100	
**KDM7A PHD+JmjC** (38-480)	10 / 100	10 / 100	
**KDM7B PHD** (1-66)	10 / 113	10 / 100	
**KDM7B** His+Trx+**PHD** (1-66)	10 / 100	10 / 100	
**KDM7B PHD+JmjC** (1-447)	5.9 / 113	7.5 / 100	7.5 / 100
**KDM7C PHD** (1-67)	10 / 113	10 / 100	
**KDM7C PHD**+JmjC+ZB (1-449)	10 / 100	10 / 100	
**DIDO1** His+Trx+**PHD** (260-325)	10 / 100	10 / 100	
**KDM5A PHD3**+GST (1542-1660)	4.6 / 50	4.0 / 50	
**Buffer only**	0 / 50		

Table S3. Concentration conditions used for each ITC experiment. **Domains**: PHD = plant homeodomain, JmjC = Jumonji-C domain. Tags: His = Histidine x6, Trx = thioredoxin, ZB = Z-basic, GST = Glutathione S-transferase.

Hydrogen Deuterium Exchange Mass Spectrometry (HDX-MS)

LEAP PAL RTC system (LEAP Technologies) was used for HDX-MS measurements and all conditions were performed in technical triplicate. Peptides were identified that covered 79% and 83% of the protein sequence for the KDM7B PHD-finger and Dual Domain constructs, respectively. Protein ± ligand samples for HDX-MS were prepared in 20 mM HEPES-Na pH 7.5, 100 mM NaCl and 1 mM TCEP and kept at 4 °C, unless otherwise stated. Complexes were pre-formed and incubated for 30 minutes, using 10 µM protein and 20 µM compound, with DMSO constant at 1 % (v/v).

Deuterium exchange reactions were initiated by diluting the protein/ligand stock solutions 20-fold (final 0.5 µM protein and 1 µM compound) in 20 mM HEPES-Na pH 7.5, 100 mM NaCl, 1 mM TCEP prepared in D_2O (99.9% atom D, Sigma Aldrich; labelling buffer 96% (v/v) D_2O; labelling reaction 91.2% (v/v) D_2O), and incubating at 20 °C for 60 s, 300 s, 900 s, 1800 s and 3600 s. Undeuterated controls were prepared by performing the same dilution but in buffer prepared in H_2O. The labelling reactions were quenched by transferring 50 µL of the reaction mixture to 50 µL of pre-chilled quench solution (3.0 M urea, 1.6% (v/v) formic acid in water; 2 °C). Quenched samples were injected directly onto an Enzymate BEH immobilised pepsin column (2.1 x 300 mm, 3 µm; Waters) at 100 µL/min at 20 °C for 3 min at 10,000 psi. Peptic peptides were trapped and desalted on an Acquity BEH C18 VanGuard pre-column (130 Å, 2.1 x 5 mm, 1.7 µm; Waters) kept at 0.1 °C. The trapped peptides were eluted using a 6 min gradient of 5-35% acetonitrile in 0.1% (v/v) formic acid at 40 µL/min on an Acquity UPLC BEH C18 column (130 Å, 1.7 µm, 100 mm x 1 mm; Waters) at 0.1 °C. Peptides were detected on a SYNAPT G2-Si HDMS mass spectrometer (Waters) acquiring over an m/z range of 50–2000 with an electrospray source and lock mass calibration (Leucine Enkephalin, 200 pg/µL; Waters). The mass spectrometer was operated at a source temperature of 80 °C and a spray voltage of 2.5 kV. Spectra were collected in positive ion resolution mode. Peptide identification was performed in Protein Lynx Global Server (Waters) using MS^E data collected for the undeuterated control samples. The resultant peptide lists were imported into DynamX (Waters) where peptides were filtered: minimum intensity of 5000, minimum of 0.2 products per amino acid, a maximum MH^+ error of 5 ppm, and found in all of the undeuterated datasets. The automatic peptide assignment in DynamX was performed using the standard parameters, but charge state assignment and retention times were verified manually for all peptides. Data were not corrected for back-exchange, so are relative rather than absolute deuterium uptake values. The first two residues of each peptide are excluded from the analyses due to rapid back-exchange.

A Student's t-test was performed on the peptide-level data using the pooled standard deviation of all peptides across all samples and time-points (Pooled SD(DualDomain) = 0.08 Da; Pooled SD(PHD) = 0.05 Da). Peptides with a p-value <0.01 were considered significant and taken forward in the analyses. A custom MATLAB script was used to combine data from redundant peptides to generate a residue-level view of the differences in deuterium uptake for each time point.

Ligand	Stock Solution		Labelling Reaction		Replicates
	Protein (µM)	Ligand (µM)	Protein (µM)	Ligand (µM)	
DMSO	10	-	0.5	-	3
$H3_{1-21}$ K4me3	10	20	0.5	1	3
OC3	10	20	0.5	1	3
OC9	10	20	0.5	1	3

Table S4. Conditions used for each hydrogen-deuterium exchange mass spectrometry (HDX-MS) experiments. Protein refers to either the Dual Domain or PHD alone.

The pooled variance was calculated by:

$$\sigma_p^2 = \frac{\sum_{i=1}^{k}(n_i - 1)\sigma_i^2}{\sum_{i=1}^{k}(n_i - 1)}$$

The two-tailed Student's t-test was calculated by:

$$t = \frac{|\mu_1 - \mu_2|}{\sqrt{\frac{\sigma_p^2}{n_1} + \frac{\sigma_p^2}{n_2}}}$$

AlphaScreen Displacement Assay

Displacement assays were performed using either an AlphaPlate 384 SW or white ProxiPlate 384 Plus (PerkinElmer) with buffer [50 mM HEPES pH 7.5, 150 mM NaCl, 0.01 % (v/v) Tween, 0.1 % (w/v) BSA]. $H3_{1-21}$K4me3-GGK-biotin was purchased from AnaSpec. The linear range of signal was determined for each His-tagged protein/biotinylated peptide combination to select suitable assay conditions. Concentrations are reported as those in the final 20 µL assay volume. All KDM7 and DIDO proteins were tested with OC9 and $H3_{1-21}$K4me3 twice in technical triplicate, and with OC9 derivatives at least twice in technical duplicate, whilst other proteins were tested at least once in technical duplicate. Compound dilution series were typically prepared in aqueous 0.4 % DMSO (0.1 % DMSO final) and all other components in buffer. Compound (5 µL) was incubated with protein (5 µL) for 5 minutes at 20 °C, followed by addition of 5 µL of biotinylated peptide for 20 minutes and then addition of 5 µL of pre-mixed AlphaScreen beads (Nickel Chelate kit, PerkinElmer, 6760619M, 250-fold final dilution), then incubated at 25 °C for 90 minutes. The plate was sealed with opaque foil and pulse centrifuged briefly after each addition. The plate was read with a BMG Pherastar FS/FSX with AlphaScreen detection module (680, 570). Data were analysed using GraphPad Prism v9 and raw counts were fitted with the model: 'log(inhibitor) vs. response, variable slope'. Graphed data are also presented as '% Displacement', where each compound set was normalised to respective minima and maxima where reasonably fitted, or DMSO control where it could not be, then '% Displacement' calculated as: 100 – normalised % and plotted with the model: log(inhibitor) vs. normalized response, variable slope.

Protein (nM)	Amino Acids, Uniprot ID	Protein Tags	$H3_{1-21}$K4me3-GGK-Biotin (nM)
KDM7A PHD (15)	(38-98), (Q6ZMT4-1)	*N*-His_6+Trx	5
KDM7B PHD (10)	(1-66), (Q9UPP1-2)	*N*-His_6+Trx	5
KDM7C PHD (10)	(1-67), (O75151)	*N*-His_6+Trx	5
KDM7A PHD+JmjC (20)	(38-480), (Q6ZMT4-1)	*N*-His_6+Trx	8
KDM7B PHD+JmjC (20)	(1-447), (Q9UPP1-2)	*N*-His_6+Trx	3.2
DIDO PHD (3.2)	(260-325), (Q9BTC0)	*N*-His_6+Trx	1.28
TAF3 PHD (12.5)	(857-924), (Q5VWG9)	*N*-His_6+GST	3
KDM5A PHD3 (50)	(1542-1660), (P29375)	*N*-His_6+GST	25
ING2 PHD (25)	(171-226), (Q9H160)	*N*-His_6+GST	3
SPIN1 TTD (25)	(26-262), (Q9Y657)	*C*-His_6	6

Table S5. Conditions used for each AlphaScreen displacement assay. Trx = thioredoxin. GST = glutathione *S*-transferase.

AlphaScreen Demethylation Assay

KDM AlphaScreen activity assays were carried out similarly to those described by Hopkinson et al[8] and Rose et al[9] with the following modifications. Compound titration series were ECHO dispensed at least in duplicate (100 nL in DMSO) to a ProxiPlate (384-Plus, White, PerkinElmer) for a 10 µL total volume assay (1 % DMSO final) in assay buffer. The AlphaScreen beads (IgG-Protein A Detection Kit, Perkin Elmer, 6760617) were incubated with primary antibody for at least 30 minutes at 20 °C prior to use. Compounds were pre-incubated with 7.5 µL of enzyme for 10 minutes at 20 °C, then the demethylation initiated by addition of 2.5 µL substrate mix (2OG, ammonium iron(II) sulfate hexahydrate (Fe^{II}), sodium ascorbate (Asc) and biotinylated H3 peptide substrate in buffer). Concentrations are reported as those present in the 10 µL volume. All components of the substrate mix were prepared in assay buffer, except for Fe(II) which was initially diluted in 20 mM HCl, then MilliQ prior to mixing. The reaction was incubated at 20 °C under a foil seal, then quenched at an endpoint within the linear range of signal by addition of 5 µL of quench solution (30 mM EDTA, 1.5 M NaCl), followed by addition of 5 µL of IgG-antibody bead mix. The plate was re-sealed and further incubated at 20 °C for 1 hour, then read with a BMG Pherastar FS with AlphaScreen detection module (680, 570). Data were calculated as a % of maximum signal and analysed using GraphPad Prism v9 with the model: 'log(inhibitor) vs. normalized response, variable slope'.

Protein (nM)	Biotinylated Peptide Substrate (nM)	Co-Factor (µM)			Assay Buffer	Time (min)	Antibody (Dilution)
		Fe(II)	Asc	2-OG			
KDM7B PHD+JmjC (20)	$H3_{1-21}$ K4me3K9me2-GGK-biotin (150)	10	100	20	50 mM HEPES, pH 7.05, 50 mM NaCl, 0.1 % (w/v) BSA, 0.01 % (v/v) Tween20	10	Anti-H3K9me1, Ab8896 (10,000)

Table S6. Conditions used for the KDM7B AlphaScreen demethylation assay.

MALDI-TOF-MS KDM7 Activity Assay

MALDI-TOF mass spectra were obtained using a Bruker MALDI-TOF Microflex LRF (positive, reflectron mode) by spotting 0.8 µL with 0.8 µL of α-cyano-4-hydroxycinnamic acid (CHCA) matrix (dissolved in 50 % acetonitrile/50 % H_2O/0.1 % TFA v/v). Dose-response IC/EC_{50} assays with KDM7A and KDM7B used 96-well PCR plates (E1403-5200, Starlab) on a ThermoMixer C (Eppendorf). Compounds were tested twice in technical triplicate and their dilution series prepared in aqueous 1 % DMSO (0.2 % final). 5 µL of protein in assay buffer (50 mM HEPES pH 7.05, 100 mM NaCl, 1 mM TCEP) was added to 2 µL of compound by pulse centrifugation and pre-incubated at the respective assay temperature for 5 minutes. Initiation was performed by simultaneous addition to every well by pulse centrifugation of 3 µL substrate mix (final concentrations of 100 µM 2OG, 100 µM Fe^{II}, 200 µM Asc, and 2.5 µM $H3_{1-21}$K4me3K9me2 or $H3_{1-21}$K9me2 (GL Biochem)). All components of the substrate mix were prepared in assay buffer, except for Fe(II) initially diluted in 20 mM HCl, then MilliQ before mixing. All wells were simultaneously quenched (3 µL of 6 % (v/v) formic acid) at a timepoint within the linear range of activity (time-courses were run under the same conditions prior to compound testing). The assay mix was analysed directly by MALDI-TOF-MS (Bruker flexAnalysis v3.4). The '% demethylation' was determined by comparison of the relative intensities of monoisotopic peaks corresponding to the K9me2/1/0 states, then fitted with GraphPad Prism v9, using the model: 'log(inhibitor) vs. response, variable slope'. Individual experiment conditions are given in Supplementary Methods. Assays were carried out twice in triplicate technical replicates.

Enzyme (µM)	Protein Tags?	Substrate Conditions	Co-Factors Fe^{II}, Asc, 2OG (µM)
KDM7A PHD+JmjC (0.55)	*N*-His_6+Trxt	$H3_{1-21}$K4me3K9me2 (2.5 µM, 30 °C, 10.5 min) and $H3_{1-21}$K9me2 (2.5 µM, 30 °C, 10.5 min)	100, 200, 100
KDM7B PHD+JmjC (0.40)	*N*-His_6+Trxt	$H3_{1-21}$K4me3K9me2 (2.5 µM, 25 °C, 6 min) and $H3_{1-21}$K9me2 (2.5 µM, 30 °C, 9.5 min)	100, 200, 100

Table S7. Summary of assay conditions used in KDM7 MALDI-TOF-MS dose-response demethylation assays.

KDM Selectivity Screening

Compound screening at single concentrations was performed in a 96-well PCR plate (4ti-0740, 4titude) on a ThermoMixer C (Eppendorf). Compounds were tested once in either technical duplicate or triplicate and typically diluted in aqueous 5 % DMSO (1 % final). Protein in assay buffer (4 µL) was added to 2 µL of compound and pre-incubated at 20 °C for 10 minutes. The reaction was initiated by addition of 4 µL substrate mix (2OG, Fe^{II}, Asc, and histone peptide substrate (GL Biochem)). All components of the substrate mix were prepared in assay buffer, except for Fe^{II} which was initially diluted in 20 mM HCl, then MilliQ prior to mixing. All wells were quenched (4 µL of 6 % (v/v) formic acid) within a time-course assay determined linear range of activity. The assay mix was analysed directly by MALDI-TOF-MS. The % demethylation was determined by analysis (Bruker flexAnalysis v3.4) of the monoisotopic peak intensities corresponding to affected methylation states. Data were further analysed with GraphPad Prism v9, using an ordinary one-way ANOVA test, assuming a normal distribution and common variance, followed by Dunnett's multiple comparison test (comparing the mean value for each compound result with the mean value of the DMSO-control). The significance thresholds of the resulting multiplicity adjusted P-values were used to label the data (noted in the figure legends).

Enzyme (µM)	Protein Tags	Substrate and Buffer Conditions	Co-Factors: Fe^{II}, Asc, 2OG (µM)	Replicates per Point
KDM2A (0.3)	*N*-FLAG	$H3_{30-41}$K36me2 (10 µM, 37 °C, 25 min), 50 mM HEPES, pH 7.05, 50 mM NaCl, 1 mM TCEP	50, 100, 50	2
KDM2B (0.2)	*N*-FLAG	$H3_{30-41}$K36me2 (10 µM, 37 °C, 30 min), 50 mM HEPES, pH 7.05, 50 mM NaCl, 1 mM TCEP	50, 100, 50	3
KDM3A (0.10)	*N*-His_{10}	$H3_{1-21}$K9me2 (10 µM, 20 °C, 7 min), 50 mM HEPES, pH 7.05, 50 mM NaCl, 1 mM TCEP	50, 100, 50	3
KDM4A (1.0)	*N*-His_6	$H3_{1-21}$K9me3 (10 µM, 37 °C, 10 min), 50 mM HEPES, pH 7.05, 50 mM NaCl, 1 mM TCEP	50, 100, 50	3
KDM5C (0.25)	*C*-His_{10}	$H3_{1-21}$K4me3 (10 µM, 37 °C, 10 min), 50 mM HEPES, pH 7.05, 50 mM NaCl, 1 mM TCEP	50, 100, 50	3
KDM6B (0.50)	*N*-His_6+ Trx	$H3_{21-44}$K27me3 (10 µM, 25 °C, 7 min), 50 mM HEPES, pH 7.50, 150 mM NaCl	10, 100, 50	3
KDM7B (2.0)	*N*-His_6+ Trx	$H3_{1-21}$K4me3K9me2 (5 µM, 30 °C, 6 min), 50 mM HEPES, pH 7.05, 50 mM NaCl, 1 mM TCEP	50, 100, 50	3

Table S8. Conditions used for MALDI-TOF-MS demethylation assay selectivity screening.

Name (Amino Acids, Uniprot ID)	Domains Present
KDM2A (1-1162, Q9Y2K7)	JmjC, PHD, ZF, F-box
KDM2B (1-1336, Q8NHM5)	JmjC, PHD, ZF, F-box
KDM3A (515-1317, Q9Y4C1)	JmjC, ZF
KDM4A (1-359, O75164)	JmjC, JmjN
KDM5C (1-765, P41229)	JmjC, PHD, ZF, JmjN, ARID
KDM6B (1141-1641, O15504)	JmjC
KDM7B (1-447, Q9UPP1-2)	JmjC, PHD

Table S9. Details of the proteins used for MALDI-TOF-MS demethylation assay screening.

Calf Histone Demethylation Assay

8 µL of enzyme, either KDM7B (1 µM final) or KDM7A (0.5 µM final) dual domain, in buffer (50 mM HEPES, 50 mM NaCl, 1 mM TCEP, pH 7.4.) was pre-incubated with 4 µL of compound (20 µM) in aqueous DMSO (0.4 % in final assay volume), for 5 minutes at 37 °C, followed by addition of 8 µL of substrate mix containing Asc (1 mM final), 2OG (1 mM final), Fe^{II} (0.1 mM final), and calf thymus histone extracts (50 µg/mL final, Type IIA, H9250, Sigma Aldrich). Samples were quenched at different time intervals by addition of 2 µL aqueous 10 % (v/v) formic acid. Each timepoint sample (12 µL) was analysed by Western Blot - 12 µL of assay mixture was mixed with 4 µL 4X NuPAGE LDS loading buffer with 10 mM DTT in a PCR tube at 95 °C for 6 minutes, then loaded to a 12 % Bis-Tris gel (15-well, NuPAGE, 1.0 mm) for 50 mins at 180 V in MES buffer, then transfer by iBlot (20 V for 6 mins) and imaging, see Table S10 for antibodies. Relative H3K9me2 band intensities were calculated using BioRad ImageLab relative to their respective H4 intensities and normalised to their respective compound zero timepoint. Data were fitted using GraphPad Prism v9 with an exponential decay model (Y=(Y0 - Plateau)*exp(-K*X) + Plateau) to estimate the relative rate constants, (Y0 = 1 and shared plateau). Assays were conducted three times in a 96-well PCR plate (4ti-0740, 4titude) on a ThermoMixer C 96-block (Eppendorf).

Cell permeability measurement for OC9

Caco-2 cells (American Type Culture Collection number HTB-37) were grown on Transwell inserts (24 well HTS Transwell microplate, Corning Inc.) for 14 d until a confluent monolayer of differentiated cells was achieved. Test compounds were dissolved in DMSO to a concentration of 2 mM and then further diluted in Hanks Balanced Salt Solution (HBSS) (containing 25 mM 4-morpholineethanesulfonic acid (MES), pH 6.5, 50 µM quinidine, 30 µM benzbromarone and 20 µM sulfasalazine) to give a final concentration of 5 µM. The culture medium was removed from the Transwell and the test compound solution (210 µL) was added to the Transwell insert (apical compartment) and 800 µL of HBSS receiver buffer (containing 25 mM *N*-(2-hydroxyethyl)piperazine-*N′*-(2-ethanesulfonic acid) (HEPES), pH 7.4, 50 µM quinidine, 30 µM benzbromarone, 20 µM sulfasalazine) added to the wells in the receiver plate (basolateral compartment). The plate was incubated at 37 °C with rotary shaking at 480 rpm. Samples (10 µL) were taken from the apical (donor) side immediately after test compound solution addition, and following 45 and 120 min of incubation. These were diluted in HBSS receiver buffer (90 µL). Samples (100 µL) were taken from the basolateral (receiver) side following 45 and 120 min of incubation. Fresh HBSS receiver buffer (100 µL) was added to replace the volume taken. The samples were protein precipitated with acetonitrile (300 µL) containing internal standards (alprazolam 100 nM, caffeine 200 nM and tolbutamide 100 nM), vortexed for 5 min, and centrifuged (4000 rpm for 20 min). The supernatant was analysed for test compound concentration against a standard curve from 2.1 to 1500 nM using LC/MS/MS. The apparent permeability (P_{app}) in units of centimetres per second, was calculated using equation where C_R is the concentration of the receiver side, C_D is the concentration of the donor side, at the respective time points, *area* is the is the surface area of the Transwell insert membrane (0.33 cm^2), and *time* is the time between difference between the two samplings (4500 s).

$$P_{app} = \frac{C_R^{120} \times 0.8 - C_R^{45} \times 0.7}{(C_D^{45} + C_D^{120})/2 \times area \times time}$$

Western Blot

Wet Transfer:
The NuPAGE gel was equilibrated in transfer buffer for 15 minutes (100 mL MeOH, 100 mL 10X TG, 800 mL H_2O, chilled to 4 °C). Nitrocellulose membrane (0.45 µm, 10600003, Amersham Protran) was pre-wet in MilliQ H_2O for 5 minutes. The membrane, filter paper (3 mm, Chr) and gel were assembled into the blot-stack (Mini Trans-Blot Electrophoretic Transfer Cell, BioRad) according to manufacturer's instructions. Transfer was performed at 100 V for 60 minutes, at 4 °C.

Imaging:
Membranes were blocked in PBS-T with 5 % milk (skim milk powder 70166, Sigma Aldrich) for 60-90 minutes, then washed (3x PBS-T for 5 minutes). Primary antibody (in PBS with 3 % BSA and 0.02 % NaN_3) was added and incubated for 16-24 hours at 4 °C with gentle rocking. The primary antibody solution was recovered, and the membrane washed (3x PBS-T) before addition of secondary antibody (in PBS-T with 5 % milk) for 1 hour at 20 °C with gentle rocking. Membranes were then washed thoroughly (5x PBS-T for 5 minutes) and imaged by electrochemiluminescence (ECL) using SuperSignal West Pico PLUS Chemiluminescent Substrate (ThermoFisher), according to the manufacturer's instructions, on a ChemiDoc MP (BioRad).

Antibodies

**Class**	**Target**	**Name**	**Source**	**Dilution**	**Species**	**Type**
Primary	H3K9me2	Ab1220	Abcam	1000	Mouse	mAb
Primary	H4	Ab177840	Abcam	1000	Rabbit	r-mAb
Primary	KDM7B/PHF8 (AAs 800-900)	Ab36068	Abcam	500	Rabbit	pAb
Primary	KDM7C/PHF2	D45A2	CST	1000	Rabbit	mAb
Secondary	HRP-Anti-Mouse IgG	PI-2000	Vector Laboratories	5000	Horse	
Secondary	HRP-Anti-Rabbit IgG	PI-1000	Vector Laboratories	5000	Goat	

Table S10. Antibodies used for Western Blotting and their respective dilutions.

Pull-down and Proteomics from SUP-T1 cells

SUP-T1 cells were cultured in RPMI 1640 supplemented with 10 % FBS (heat inactivated), 1 % penicillin/streptomcyin and 1 % L-glutamine. Cell pellets ($3x10^7$ cells) were processed using a Nuclear Complex Co-Ip isolation kit (ActiveMotif, 54001) on ice according to the manufacturer's instructions, but omitting EDTA. The extracted nuclear lysates (700 µL) were measured by bicinchoninic acid (BCA) assay (Pierce BCA Protein Assay Kit 23227 and Albumin Standards 23209, Thermo Scientific) (used at 0.84 mg/mL). Low bind tubes were used throughout. Lysate (650 µL) was pre-cleared with 140 µL of magnetic streptavidin beads (Dynabeads M-280 Streptavidin 11206D, Invitrogen) (prepared by washing once in PBS). OC9 (1 µL, 20, 5, 1, 0.1, 0 µM final) was added to 50 µL of pre-cleared lysate in duplicate on ice for 15 mins (0.1 % v/v DMSO final in lysate). OC9-Biotin (1.7 µL of 30 µM, 1 µM final) was added to each, and Biotin was added (1 µM final) to an additional duplicate DMSO control, on ice for 30 mins. Solutions were then used to re-suspend 30 µL worth of pre-washed magnetic streptavidin beads in fresh tubes rotated at 4 °C for 20 mins. Unbound supernatants were then removed, and beads re-suspended in 50 µL of ice-cold PBS then immediately transferred to a new tube and supernatant again removed. Finally, beads were re-suspended in 50 µL of elution buffer (250 µL 4X lithium dodecyl sulfate (LDS), 750 µL water, 20 µL 1 M dithiothreitol (DTT) (20 mM final)) and heated at 95 °C for 5 mins, with elute recovered immediately whilst hot to a fresh tube. Pre-cleared input (15 µL) was added to 5 µL of 4X LDS and 1 µL 1 M DTT. All samples were stored for short-term at -20 °C. Each elute sample (5 µL) and input was analysed by Western blot for KDM7B and KDM7C (see Table S10 for antibodies).

MS sample preparation: Eluted samples (25 µL) were diluted to 200 µL in 0.1 M Tris buffer (pH 7.8), reduced with DTT (5 mM final concentration) for 1 h at room temperature, and alkylated with iodoacetamide (20 mM final concentration) for 30 mins at room temperature in the dark. Protein was precipitated by sequential addition of MeOH (600 µL), $CHCl_3$ (150 µL), and H_2O (450 µL), vortexing between each addition. The mixture was spun (17,000 *g*, 5 min) and the aqueous layer removed without disturbing the peptide precipitate at the interface. Further MeOH was added (450 µL), the protein precipitate pelleted by centrifugation (17,000 *g*, 5 min), and the supernatant removed. The pellet was washed with MeOH (600 µL), re-pelleted, and air dried for 10 minutes at room temperature before resuspension in 6 M urea (pH 7.8, 50 µL) by vortexing. The peptide solution was diluted with 250 µL H_2O and incubated with trypsin (0.8 µg) overnight at 37°C. The digests were acidified with formic acid (FA) (1% v/v final concentration), desalted using SOLA HRP SPE Cartridges (Thermo Fisher), eluting with 69% v/v MeCN, 0.1% v/v FA in H_2O (600 µL) and dried in vacuo. Dried peptides were stored at -20°C before resuspension in (2% v/v MeCN, 0.1% v/v FA in H_2O) (20 µL) for LC-MS/MS analysis.

LC-MS/MS data acquisition: Mass spectrometry data were acquired at the Discovery Proteomics Facility (University of Oxford). Digested samples were analysed by nano-UPLC-MS/MS using a Dionex Ultimate 3000 nano UPLC fitted with an EASY spray column (75 µm × 500 mm, 2 µm particle size, Thermo Scientific), coupled to an Orbitrap Q Exactive instrument. A 60 min gradient of 0.1% (v/v) formic acid in 5% (v/v) DMSO to 0.1% (v/v) formic acid with 35% (v/v) acetonitrile in 5% (v/v) DMSO at a flow rate of 250 nL min^{-1} was used. The instrument was operated in a data-dependant manner, with survey scans acquired at a resolution of 70,000 at 200 m/z and the 15 most abundant precursors selected for HCD fragmentation with an AGC target of 1×10^5 ions.

Data analysis: Raw data was analysed as previously described[10]. Briefly, data was processed using MaxQuant version 1.6.5.0 and the reference complete human proteome FASTA file (Uniprot). Label Free Quantification (LFQ) and Match Between Runs were selected; replicates were collated into parameter groups to ensure matching between replicates only. Cysteine carbamidomethylation was selected as a fixed modification, and methionine oxidation as a variable modification. Default settings for identification and quantification were used. Specifically, a minimum peptide length of 7, a maximum of 2 missed cleavage sites, and a maximum of 3 labelled amino acids per peptide were employed. Peptides and proteins were identified utilising a 0.01 false discovery rate, with "Unique and razor peptides" mode selected for both identification and quantification of proteins (razor peptides are uniquely assigned to protein groups and not to individual proteins). At least 2 razor + unique peptides were required for valid quantification. Processed data was further analysed using Perseus version 1.6.5.0. Peptides categorised by MaxQuant as 'potential contaminants', 'only identified by site' or 'reverse' were filtered, and the LFQ intensities transformed by Log2. Experimental replicates were

grouped, and the data filtered such that 2 valid LFQ values were required for the condition without any OC9 competition, and at least one valid LFQ value in each of the remaining experimental groups. Missing values were imputed using default settings, and the data distribution visually inspected to ensure that a normal distribution was maintained. Statistically significant competition was determined through the application of P2 tests, using a permutation-based FDR of 0.05 and volcano plot visualisation.

Data deposition: The mass spectrometry proteomics data have been deposited to the ProteomeXchange Consortium via the PRIDE partner repository[11] with the data set identifier PXD027151.

Pull-down from Transfected HEK293T Cells

HEK293T cells were cultured in DMEM supplemented with 10% FBS, 1 % penicillin/streptomcyin and 1 % L-glutamine. Cells were transiently transfected (6-well plate, 800k cells per well, with 1 µg DNA, 8 µL FuGene, 100 µL Opti-MEM per well) using 3 wells for each plasmid (plasmid backbone in pFC32K containing KDM7B.v.1 and a C-terminal nano luciferase tag; either KDM7B(WT, 1-1060)-NLuc, or KDM7B(PHD_Mutant[Y18A, W29A])-NLuc). Cells were incubated for 18 hours at 37 °C with 5% CO_2 and harvested by centrifugation at 100 g. Cells were exchanged into OptiMEM media immediately prior to final centrifugation aliquotting. Low-binding tubes were used for all following steps. $2x10^6$ cells were processed with the Nuclear Extraction kit (Ab219177, Abcam) according the manufacturer's instructions which enabled separation of cytoplasmic, soluble nuclear and insoluble nuclear fractions.

The soluble nuclear lysate was used for the pull-down experiment and the total amount of protein present within the sample was quantified by bicinchoninic acid (BCA) assay (Pierce BCA Protein Assay Kit 23227 and Albumin Standards 23209, Thermo Scientific). The soluble nuclear lysates from each cell pellet were then diluted in PBS-T to a 0.5 mg/mL of 500 µL. Magnetic streptavidin beads (Dynabeads M-280 Streptavidin 11206D, Invitrogen) were prepared by washing three times in equal volumes of PBS-T before final re-suspension to their original volume. A pre-clear step was performed by incubating 0.25 lysate-volumes' worth of beads alone with the lysate with rotation for 15 minutes at 4 °C. Magnetic beads used for the pre-clear were removed by magnetic separation. The pre-cleared soluble nuclear lysate of each WT and MUT KDM7B was aliquoted (3x 100 µL), with an additional and 2x 50 µL for WT. The 3x 100 µL aliquots were used for the primary pull-downs (OC9-Bt, H3K4me3-Bt, Biotin control), and the 2x 50 µL for competition pull-downs (OC9-Bt plus unlabelled OC9/OC9-V10A). Pull-down probes were added (< 5 µL additional solution, 1 µM final) and incubated with rotation for 30 minutes at 4 °C. For competition experiments, the unlabelled competitor was also added at this stage from a concentrated DMSO stock to a final concentration of 100 µM (final DMSO < 2 % for all pull-downs). 30 µL of magnetic streptavidin beads were then added and incubated with rotation for 15 minutes at 4 °C. Beads were recovered by magnetic separation and washed (3x 30 µL PBS-T), transferring to a new low-binding tube between each wash, before final re-suspension in 30 µL of loading buffer (PBS-T and NuPAGE 4X LDS Sample Buffer NP007, ThermoFisher, with 1 mM DTT). The suspended beads were heated at 95 °C for 5 minutes and the supernatant recovered to a new low-binding tube as the final pull-down sample. 3 µL of each direct pull-down sample, 6 µL of competition samples and 10 µL of input (pre-cleared soluble nuclear lysate) were loaded to SDS-PAGE gel (1 mm 4-12% Bis-Tris NuPAGE gel in MES buffer at 200 V for 45 mins). Further analysis of the gel was performed by Western Blot.

Full-Length KDM7B Plasmids and Mutagenesis
Full-length KDM7B.v.1 protein sequence:

MNRSRAIVQRGRVLPPPAPLDTTNLAGRRTLQGRAKMASVPVYCLCRLPYDVTRFMIECDMCQDWFHGSCVGVEE
EKAADIDLYHCPNCEVLHGPSIMKKRRGSSKGHDTHKGKPVKTGSPTFVRELRSRTFDSSDEVILKPTGNQLTVE
FLEENSFSVPILVLKKDGLGMTLPSPSFTVRDVEHYVGSDKEIDVIDVTRQADCKMKLGDFVKYYYSGKREKVLN
VISLEFSDTRLSNLVETPKIVRKLSWVENLWPEECVFERPNVQKYCLMSVRDSYTDFHIDFGGTSVWYHVLKGEK
IFYLIRPTNANLTLFECWSSSSNQNEMFFGDQVDKCYKCSVKQGQTLFIPTGWIHAVLTPVDCLAFGGNFLHSLN
IEMQLKAYEIEKRLSTADLFRFPNFETICWYVGKHILDIFRGLRENRRHPASYLVHGGKALNLAFRAWTRKEALP
DHEDEIPETVRTVQLIKDLAREIRLVEDIFQQNVGKTSNIFGLQRIFPAGSIPLTRPAHSTSVSMSRLSLPSKNG
SKKKGLKPKELFKKAERKGKESSALGPAGQLSYNLMDTYSHQALKTGSFQKAKFNITGACLNDSDDDSPDLDLDG
NESPLALLMSNGSTKRVKSLSKSRRTKIAKKVDKARLMAEQVMEDEFDLDSDDELQIDERLGKEKATLIIRPKFP
RKLPRAKPCSDPNRVREPGEVEFDIEEDYTTDEDMVEGVEGKLGNGSGAGGILDLLKASRQVGGPDYAALTEAPA
SPSTQEAIQGMLCMANLQSSSSSPATSSLQAWWTGGQDRSSGSSSSGLGTVSNSPASQRTPGKRPIKRPAYWRTE
SEEEEENASLDEQDSLGACFKDAEYIYPSLESDDDDPALKSRPKKKKNSDDAPWSPKARVTPTLPKQDRPVREGT
RVASIETGLAAAAAKLAQQELQKAQKKKYIKKKPLLKEVEQPRPQDSNLSLTVPAPTVAATPQLVTSSSPLPPPE
PKQEALSGSLADHEYTARPNAFGMAQANRSTTPMAPGVFLTQRRPSVGSQSNQAGQGKRPKKGLATAKQRLGRIL
KIHRNGKLLL

N-terminal HaloTag in pFN21A was purchased from Promega (FHC01149) and verified by Sanger sequencing. The Flexi Cloning System (Promega) was used to sub-clone the KDM7B.v.1 sequence into a *C*-terminal NLuc tag plasmid (pFC32K).

Site directed mutagenesis (SDM) of full-length KDM7B in pFC32K was performed according to standard molecular biology PCR protocols. Mutagenesis primers for the KDM7B PHD finger (for v.1. residue numbering is: Y50A, W65A), (for v.2. residue numbering is: Y14A, W29A):

Forwards SDM Primer:

5’-CTGCCTgcCGATGTGACCCGCTTCATGATCGAGTGTGACATGTGCCAGGACgcGTTTCATGGCAGTTG-3’

Reverse SDM Primer:

5’-GAAACgcGTCCTGGCACATGTCACACTCGATCATGAAGCGGGTCACATCGgcAGGCAGCCGGCAGAG-3’

PCR Reaction: The PCR reaction was assembled in a PCR tube and contained: 100 ng plasmid template (full-length KDM7B wild-type, sequence verified), 0.2 µM forwards primer, 0.2 µM reverse primer, 250 µM dNTPs, 5 µL 10X PfuUltra II reaction buffer, 1 µL PfuUltra II fusion HS DNA polymerase, MilliQ H_2O up to 50 µL. PCR Parameters: 98 °C 2 min; [98 °C 20 sec, 55 °C 20 sec, 72 °C 6 min (60 sec / kb)] x20; 72 °C 3 min. Followed by digestion with 10 U DpnI at 37 °C for 2 h. Then 2 µL of the SDM reaction was transformed into 50 µL of NEB 5-alpha cells and used for overnight culture growth for mini-prep (Macherey Nagel). Successful mutagenesis of target sites was confirmed by Sanger sequencing.

Supplementary References

1. Wishart, D. S., Bigam, C. G., Holm, A., Hodges, R. S. & Sykes, B. D. 1H, 13C and 15N random coil NMR chemical shifts of the common amino acids. I. Investigations of nearest-neighbor effects. *J Biomol NMR* **5**, 67–81 (1995).
2. Goto, Y., Goto, Y., Katoh, T. & Suga, H. Preparation of materials for flexizyme reactions and genetic code reprogramming. *Protocol Exchange* **100**, 1–14 (2011).
3. Hayashi, Y., Morimoto, J. & Suga, H. In vitro selection of anti-Akt2 thioether-macrocyclic peptides leading to isoform-selective inhibitors. *ACS Chemical Biology* **7**, 607–613 (2012).
4. Goto, Y., Katoh, T. & Suga, H. Flexizymes for genetic code reprogramming. *Nat Protoc* **6**, 779–790 (2011).
5. McAllister, T. E. *et al.* Non-competitive cyclic peptides for targeting enzyme–substrate complexes. *Chem Sci* **9**, 4569–4578 (2018).
6. Skinner, S. P. *et al.* CcpNmr AnalysisAssign: a flexible platform for integrated NMR analysis. *Journal of Biomolecular NMR* **66**, 111–124 (2016).
7. Maciejewski, M. W. *et al.* NMRbox: A Resource for Biomolecular NMR Computation. *Biophysical Journal* **112**, 1529–1534 (2017).
8. Hopkinson, R. J. *et al.* 5-Carboxy-8-hydroxyquinoline is a broad spectrum 2-oxoglutarate oxygenase inhibitor which causes iron translocation. *Chemical Science* **4**, 3110 (2013).
9. Rose, N. R. *et al.* Plant Growth Regulator Daminozide Is a Selective Inhibitor of Human KDM2/7 Histone Demethylases. *J Med Chem* **55**, 6639–6643 (2012).
10. Scheer, S. *et al.* A chemical biology toolbox to study protein methyltransferases and epigenetic signaling. *Nature Communications* **10**, 19 (2019).
11. Perez-Riverol, Y. *et al.* The PRIDE database and related tools and resources in 2019: improving support for quantification data. *Nucleic Acids Research* **47**, D442–D450 (2018).
